# Local Well-Posedness of Skew Mean Curvature Flow for Small Data in $d\ge 4$ Dimensions

**DOI:** 10.1007/s00220-021-04303-8

**Published:** 2022-01-15

**Authors:** Jiaxi Huang, Daniel Tataru

**Affiliations:** 1 Beijing International Center for Mathematical Research, Peking University, Beijing, 100871 People’s Republic of China; 2 Department of Mathematics, University of California, Berkeley, Berkeley, CA 94720 USA

## Abstract

The skew mean curvature flow is an evolution equation for *d* dimensional manifolds embedded in ${{\mathbb {R}}}^{d+2}$ (or more generally, in a Riemannian manifold). It can be viewed as a Schrödinger analogue of the mean curvature flow, or alternatively as a quasilinear version of the Schrödinger Map equation. In this article, we prove small data local well-posedness in low-regularity Sobolev spaces for the skew mean curvature flow in dimension $d\ge 4$.

## Introduction

The skew mean curvature flow (SMCF) is a nonlinear Schrödinger type flow modeling the evolution of a *d* dimensional oriented manifold embedded into a fixed oriented $d+2$ dimensional manifold. It can be seen as a Schrödinger analogue of the well studied mean curvature flow. In this article, we consider the small data local well-posedness for the skew mean curvature flow in high dimensions $d \ge 4$, for low regularity initial data.

### The (SMCF) equations

Let $\Sigma ^d$ be a *d*-dimensional oriented manifold, and $({\mathcal {N}}^{d+2},g_{{\mathcal {N}}})$ be a $d+2$-dimensional oriented Riemannian manifold. Let $I=[0,T]$ be an interval and $F:I\times \Sigma ^d \rightarrow {\mathcal {N}}$ be a one parameter family of immersions. This induces a time dependent Riemannian structure on $\Sigma ^d$. For each $t\in I$, we denote the submanifold by $\Sigma _t=F(t,\Sigma )$, its tangent bundle by $T\Sigma _t$, and its normal bundle by $N\Sigma _t$ respectively. For an arbitrary vector *Z* at *F* we denote by $Z^\perp$ its orthogonal projection onto $N\Sigma _t$. The mean curvature ${\mathbf {H}}(F)$ of $\Sigma _t$ can be identified naturally with a section of the normal bundle $N\Sigma _t$.

The normal bundle $N\Sigma _t$ is a rank two vector bundle with a naturally induced complex structure *J*(*F*) which simply rotates a vector in the normal space by $\pi /2$ positively. Namely, for any point $y=F(t,x)\in \Sigma _t$ and any normal vector $\nu \in N_{y}\Sigma _t$, we define $J(F)\nu \in N_{y}\Sigma _t$ as the unique vector with the same length so that

$$\begin{aligned} J(F)\nu \bot \nu , \qquad \omega (F_{*}(e_1), F_{*}(e_2),\ldots F_{*}(e_d), \nu , J(F)\nu )>0, \end{aligned}$$

where $\omega$ is the volume form of ${\mathcal {N}}$ and $\{e_1,\ldots ,e_d\}$ is an oriented basis of $\Sigma ^d$. The skew mean curvature flow (SMCF) is defined by the initial value problem

1.1 $$\begin{aligned} \left\{ \begin{aligned}&(\partial _t F)^{\perp }=J(F){\mathbf {H}}(F),\\&F(\cdot ,0)=F_0, \end{aligned}\right. \end{aligned}$$

which evolves a codimension two submanifold along its binormal direction with a speed given by its mean curvature.

The (SMCF) was derived both in physics and mathematics. The one-dimensional (SMCF) in the Euclidean space ${{\mathbb {R}}}^3$ is the well-known vortex filament equation (VFE)

$$\begin{aligned} \partial _t \gamma =\partial _s \gamma \times \partial _s^2 \gamma , \end{aligned}$$

where $\gamma$ is a time-dependent space curve, *s* is its arc-length parameter and $\times$ denotes the cross product in ${{\mathbb {R}}}^3$. The (VFE) was first discovered by Da Rios [6] in 1906 in the study of the free motion of a vortex filament.

The (SMCF) also arises in the study of asymptotic dynamics of vortices in the context of superfluidity and superconductivity. For the Gross-Pitaevskii equation, which models the wave function associated with a Bose-Einstein condensate, physics evidence indicates that the vortices would evolve along the (SMCF). An incomplete verification was attempted by Lin [20] for the vortex filaments in three space dimensions. For higher dimensions, Jerrard [14] proved this conjecture when the initial singular set is a codimension two sphere with multiplicity one.

The other motivation is that the (SMCF) naturally arises in the study of the hydrodynamical Euler equation. A singular vortex in a fluid is called a vortex membrane in higher dimensions if it is supported on a codimension two subset. The law of locally induced motion of a vortex membrane can be deduced from the Euler equation by applying the Biot-Savart formula. Shashikanth [24] first investigated the motion of a vortex membrane in ${{\mathbb {R}}}^4$ and showed that it is governed by the two dimensional (SMCF), while Khesin [18] then generalized this conclusion to any dimensional vortex membranes in Euclidean spaces.

From a mathematical standpoint, the (SMCF) equation is a canonical geometric flow for codimension two submanifolds which can be viewed as the Schrödinger analogue of the well studied mean curvature flow. In fact, the infinite-dimensional space of codimension two immersions of a Riemannian manifold admits a generalized Marsden-Weinstein sympletic structure, and hence the Hamiltonian flow of the volume functional on this space is verified to be the (SMCF). Haller–Vizman [12] noted this fact where they studied the nonlinear Grassmannians. For a detailed mathematical derivation of these equations we refer the reader to the article [28, Section 2.1].

The study of higher dimensional (SMCF) is still at its infancy compared with its one-dimensional case. For the 1-d case, we refer the reader to the survey article of Vega [29]. For the higher dimensional case, Song–Sun [28] proved the local existence of (SMCF) with a smooth, compact oriented surface as the initial data in two dimensions, then Song [27] generalized this result to compact oriented manifolds for all $d\ge 2$ and also proved a corresponding uniqueness result. Recently, Li [19] considered the transversal small pertubations of Euclidean planes under the (SMCF) and proved the global regularity for small initial data. In addition, Song [26] also proved that the Gauss map of a *d* dimensional (SMCF) in ${{\mathbb {R}}}^{d+2}$ satisfies a Schrödinger Map type equation but relative to the varying metric. We remark that in one space dimension this is exactly the classical Schrödinger Map type equation, provided that one chooses suitable coordinates, i.e. the arclength parametrization.

As written above, the (SMCF) equations are independent of the choice of coordinates in $I \times \Sigma$; here we include the time interval *I* to emphasize that coordinates may be chosen in a time dependent fashion. The manifold $\Sigma ^d$ simply serves to provide a parametrization for the moving manifold $\Sigma _t$; it determines the topology of $\Sigma _t$, but nothing else. Thus, the (SMCF) system written in the form (1.1) should be seen as a geometric evolution, with a large gauge group, namely the group of time dependent changes of coordinates in $I \times \Sigma$. In particular, interpreting the equations (1.1) as a nonlinear Schrödinger equation will require a good gauge choice. This is further discussed in Sect. 2.

In this article we will restrict ourselves to the case when $\Sigma ^d = {{\mathbb {R}}}^d$, i.e. where $\Sigma _t$ has a trivial topology. We will further restrict to the case when ${\mathcal {N}}^{d+2}$ is the Euclidean space ${{\mathbb {R}}}^{d+2}$. Thus, the reader should visualize $\Sigma _t$ as an asymptotically flat codimension two submanifold of ${{\mathbb {R}}}^{d+2}$.

### Scaling and function spaces

To understand what are the natural thresholds for local well-posedness, it is interesting to consider the scaling properties of the solutions. As one might expect, a clean scaling law is obtained when $\Sigma ^d = {{\mathbb {R}}}^d$ and ${\mathcal {N}}^{d+2} = {{\mathbb {R}}}^{d+2}$. Then we have the following

#### Proposition 1.1

(Scale invariance for (SMCF)). Assume that *F* is a solution of (1.1) with initial data $F(0)=F_0$. If $\lambda >0$ then $\tilde{F}(t,x)\,{:}{=}\,\lambda ^{-1}F(\lambda ^2 t,\lambda x)$ is a solution of (1.1) with initial data $\tilde{F}(0)=\lambda ^{-1}F_0(\lambda x)$.

#### Proof

Since the induced metric and Christoffel symbols of the immersion $\tilde{F}$ are

$$\begin{aligned} \tilde{g}_{\alpha {\beta }}(t,x)=\langle \partial _{\alpha } \tilde{F},\partial _{{\beta }}\tilde{F}\rangle =g_{\alpha {\beta }}(\lambda ^2 t,\lambda x), \end{aligned}$$

and

$$\begin{aligned} \tilde{\Gamma }_{\alpha {\beta }}^{\gamma }(t,x)=\lambda \Gamma _{\alpha {\beta }}^{\gamma }(\lambda ^2t,\lambda x). \end{aligned}$$

Then by the relation ${\mathbf {H}}(F)=g^{\alpha {\beta }}(\partial _{\alpha {\beta }}^2F-\Gamma _{\alpha {\beta }}^{\gamma }\partial _{\gamma }F)$, we have

$$\begin{aligned} (\partial _t \tilde{F})^{\perp }&= \lambda (\partial _tF)^{\perp }(\lambda ^2 t,\lambda x)=\lambda Jg^{\alpha {\beta }}(\lambda ^2 t,\lambda x)[(\partial ^2_{\alpha {\beta }}F-\Gamma _{\alpha {\beta }}^{\gamma }\partial _{\gamma }F)(\lambda ^2 t,\lambda x)]\\&= J \tilde{g}^{\alpha {\beta }}(\partial ^2_{\alpha {\beta }}\tilde{F}-\tilde{\Gamma }_{\alpha {\beta }}^{\gamma }\partial _{\gamma }\tilde{F})(t,x). \end{aligned}$$

$$\square $$

The above scaling would suggest the critical Sobolev space for our moving surfaces $\Sigma _t$ to be $\dot{H}^{\frac{d}{2}+1}$. However, instead of working directly with the surfaces, it is far more convenient to track the regularity at the level of the curvature ${\mathbf {H}}(\Sigma _t)$, which scales at the level of $\dot{H}^{\frac{d}{2}-1}$.

### The main result

Our objective in this paper is to establish the local well-posedness of skew mean curvature flow for small data at low regularity. A key observation is that providing a rigorous description of fractional Sobolev spaces for functions (tensors) on a rough manifold is a delicate matter, which a-priori requires both a good choice of coordinates on the manifold and a good frame on the vector bundle (the normal bundle in our case). This is done in the next section, where we fix the gauge and write the equation as a quasilinear Schrödinger evolution in a good gauge. At this point, we content ourselves with a less precise formulation of the main result:

#### Theorem 1.2

(Small data local well-posedness). Let $s>\frac{d}{2}$, $d\ge 4$. Then there exists $\epsilon _0>0$ sufficiently small such that, for all initial data $\Sigma _0$ with metric $\Vert \partial _x(g_0-I_d)\Vert _{H^{s}}\le \epsilon _0$ and mean curvature $\Vert {\mathbf {H}}_0 \Vert _{H^s(\Sigma _0)}\le \epsilon _0$, the skew mean curvature flow (1.1) for maps from ${{\mathbb {R}}}^d$ to the Euclidean space $({{\mathbb {R}}}^{d+2},g_{{{\mathbb {R}}}^{d+2}})$ is locally well-posed on the time interval $I=[0,1]$ in a suitable gauge.

#### Remark 1.2.1

We remark on the necessity of having a smallness condition on both $g_0-I_d$ and the mean curvature ${\mathbf {H}}_0$. The combined efforts of E. De Giorgi [7], F. J. Almgren, Jr. [1], and J. Simons [25] led to the following theorem (see Theorem 4,2, [3]):

*“If* $u:{{\mathbb {R}}}^{n-1}\rightarrow {{\mathbb {R}}}$ *is an entire solution to the minimal surface equation and* $n\le 8$, *then* *u* *is an affine function."*

However, in 1969 E. Bombieri, De Giorgi, and E. Giusti [2] constructed entire non-affine solutions to the minimal surface equation in ${{\mathbb {R}}}^9$. Hence the bound $\Vert \mathbf{H}_0\Vert _{H^s(\Sigma _0)}\le \epsilon _0$ on the mean curvature does not necessarily imply that the sub-manifold is almost flat.

Here we only prove the small data local well-posedness, which means that the initial submanifold $\Sigma _0$ should be a perturbation of Euclidean plane ${{\mathbb {R}}}^d$. Hence, the bound on metric $\Vert \partial _x(g_0-I_d)\Vert _{H^{s}}\le \epsilon _0$ is also necessary in our main result, at least in very high dimension. This condition on metric will insure the existence of global harmonic coordinates (see Proposition 8.2). Later, the mean curvature bound will also yield an estimate $\Vert \partial _x (g_0-I_d)\Vert _{H^{s+1}}\lesssim \epsilon _0$ in harmonic coordinates.

Unlike any of the prior results, which prove only existence and uniqueness for smooth data, here we consider rough data and provide a full, Hadamard style well-posedness result based on a more modern, frequency envelope approach and using a paradifferential form for both the full and the linearized equations. For an overview of these ideas we refer the reader to the expository paper [13]. While, for technical reasons, this result is limited to dimensions $d \ge 4$, we expect the same strategy to also work in lower dimension; the lower dimensional case will be considered in forthcoming work.

The favourable gauge mentioned in the theorem, defined in the next section, will have two components:

- The harmonic coordinates on the manifolds $\Sigma _t$.
- The Coulomb gauge for the orthonormal frame on the normal bundle.

In the next section we reformulate the (SMCF) equations as a quasilinear Schrödinger evolution for a good scalar complex variable $\psi$, which is exactly the mean curvature but represented in the good gauge. There we provide an alternate formulation of the above result, as a well-posedness result for the $\psi$ equation. In the final section of the paper we close the circle and show that one can reconstruct the full (SMCF) flow starting from the good variable $\psi$.

One may compare our gauge choices with the prior work in [28] and [27]. There the tangential component of $\partial _t F$ in (1.1) is omitted, and the coordinates on the manifold $\Sigma _t$ are simply those transported from the initial time. The difficulty with such a choice is that the regularity of the map *F* is no longer determined by the regularity of the second fundamental form, and instead there is a loss of derivatives which may only be avoided if the initial data is assumed to have extra regularity. This loss is what prevents a complete low regularity theory in that approach.

Once our problem is rephrased as a nonlinear Schrödinger evolution, one may compare its study with earlier results on general quasilinear Schrödinger evolutions. This story begins with the classical work of Kenig–Ponce–Vega [15–17], where local well-posedness is established for more regular and localized data. Lower regularity results in translation invariant Sobolev spaces were later established by Marzuola–Metcalfe–Tataru [21–23]. The local energy decay properties of the Schrödinger equation, as developed earlier in [4, 5, 8, 9] play a key role in these results. While here we are using some of the ideas in the above papers, the present problem is both more complex and exhibits additional structure. Because of this, new ideas and more work are required in order to close the estimates required for both the full problem and for its linearization.

### An overview of the paper

Our first objective in this article will be to provide a self-contained formulation of the (SMCF) flow, interpreted as a nonlinear Schrödinger equation for a single independent variable. This independent variable, denoted by $\psi$, represents the trace of the second fundamental form on $\Sigma _t$, in complex notation. In addition to the independent variables, we will use several dependent variables, as follows:

- The Riemannian metric *g* on $\Sigma _t$.
- The (complex) second fundamental form $\lambda$ for $\Sigma _t$.
- The magnetic potential *A*, associated to the natural connection on the normal bundle $N \Sigma _t$, and the corresponding temporal component *B*.
- The advection vector field *V*, associated to the time dependence of our choice of coordinates.

These additional variables will be viewed as uniquely determined by our independent variable $\psi$, provided that a suitable gauge choice was made. The gauge choice involves two steps:

(i) The choice of coordinates on $\Sigma _t$; here we use harmonic coordinates, with suitable boundary conditions at infinity.
(ii) The choice of the orthonormal frame on $N\Sigma _t$; here we use the Coulomb gauge, again assuming flatness at infinity.

To begin this analysis, in the next section we describe the gauge choices, so that by the end we obtain

- a nonlinear Schrödinger equation for $\psi$, see (2.35).
- An elliptic fixed time system (2.36) for the dependent variables ${{\mathcal {S}}}=(g,\lambda ,V,A,B)$, together with suitable compatibility conditions (constraints).

Setting the stage to solve these equations, in Sect. 3 we describe the function spaces for both $\psi$ and ${{\mathcal {S}}}$. This is done at two levels, first at fixed time, which is useful in solving the elliptic system (2.36), and then using in the space-time setting, which is needed in order to solve the Schrödinger evolution. The fixed time spaces are classical Sobolev spaces, with matched regularities for all the components. The space-time norms are the so called local energy spaces, as developed in [21–23].

Using these spaces, in Sect. 4 we consider the solvability of the elliptic system (2.36). This is first considered and solved without reference to the constraint equations, but then we prove that the constraints are indeed satisfied.

Finally, we turn our attention to the Schrödinger system (2.35), in several stages. In Sect. 5 we establish several multilinear and nonlinear estimates in our space-time function spaces. These are then used in Sect. 6 in order to prove local energy decay bounds first for the linear paradifferential Schrödinger flow, and then for a full linear Schrödinger flow associated to the linearization of our main evolution. The analysis is completed in Sect. 7, where we use the linear Schrödinger bounds in order to (i) construct solutions for the full nonlinear Schrödinger flow, and (ii) to prove the uniqueness and continuous dependence of the solutions. The analysis here broadly follows the ideas introduced in [21–23], but a number of improvements are needed which allow us to take better advantage of the structure of the (SMCF) equations.

Last but not least, in the last section we prove that the full set of variables $(g,\lambda ,V,A,B)$ suffice in order to uniquely reconstruct the defining function *F* for the evolving surfaces $\Sigma _t$, as $H^{s+2}_{loc}$ manifolds. More precisely, with respect to the parametrization provided by our chosen gauge, *F* has regularity

$$\begin{aligned} \partial _t F,\ \partial _x^2 F \in C[0,1;H^s]. \end{aligned}$$

## The Differentiated Equations and the Gauge Choice

The goal of this section is to introduce our main independent variable $\psi$, which represents the trace of the second fundamental form in complex notation, as well as the following auxiliary variables: the metric *g*, the second fundamental form $\lambda$, the connection coefficients *A*, *B* for the normal bundle as well as the advection vector field *V*. For $\psi$ we start with (1.1) and derive a nonlinear Schödinger type system (2.35), with coefficients depending on ${{\mathcal {S}}}=(\lambda ,h,V,A,B)$, where $h=g-I_d$. Under suitable gauge conditions, the auxiliary variables ${{\mathcal {S}}}$ are shown to satisfy an elliptic system (2.36), as well as a natural set of constraints. We conclude the section with a gauge formulation of our main result, see Theorem 2.7.

We remark that H. Gomez ([11, Chapter 4]) introduced the language of gauge fields as an appropriate framework for presenting the structural properties of the surface and the evolution equations of its geometric quantities, and showed that the complex mean curvature of the evolving surface satisfies a nonlinear Schrödinger-type equation. Here we will further derive the self-contained modified Schrödinger system under harmonic coordinate conditions and Coulomb gauge.

### The Riemannian metric *g*

Let $(\Sigma ^d,g)$ be a *d*-dimensional oriented manifold and let $({{\mathbb {R}}}^{d+2},g_{{{\mathbb {R}}}^{d+2}})$ be $(d+2)$-dimensional Euclidean space. Let $\alpha ,{\beta },\gamma ,\ldots \in \{1,2,\ldots ,d\}$ and $k\in \{1,2,\ldots ,d+2\}$. Considering the immersion $F:\Sigma \rightarrow ({{\mathbb {R}}}^{d+2},g_{{{\mathbb {R}}}^{d+2}})$, we obtain the induced metric *g* in $\Sigma$,

2.1 $$\begin{aligned} g_{\alpha {\beta }}=\partial _{x_{\alpha }} F\cdot \partial _{x_{{\beta }}} F. \end{aligned}$$

We denote the inverse of the matrix $g_{\alpha {\beta }}$ by $g^{\alpha {\beta }}$, i.e.

$$\begin{aligned} g^{\alpha {\beta }}\,{:}{=}\,(g_{\alpha {\beta }})^{-1},\quad g_{\alpha \gamma }g^{\gamma {\beta }}=\delta _{\alpha }^{{\beta }}. \end{aligned}$$

Let $\nabla$ be the cannonical Levi-Civita connection in $\Sigma$ associated with the induced metric *g*. A direct computation shows that on the Riemannian manifold $(\Sigma ,g)$ we have the Christoffel symbols

$$\begin{aligned} \Gamma ^{\gamma }_{\alpha {\beta }}=\ \frac{1}{2}g^{\gamma \sigma }(\partial _{{\beta }}g_{\alpha \sigma }+\partial _{\alpha }g_{{\beta }\sigma }-\partial _{\sigma }g_{\alpha {\beta }}) =\ g^{\gamma \sigma }\partial ^2_{\alpha {\beta }}F\cdot \partial _\sigma F. \end{aligned}$$

Hence, the Laplace-Beltrami operator $\Delta _g$ can be written in the form

$$\begin{aligned} \Delta _g f&= \ {\text {tr}}\nabla ^2 f=g^{\alpha {\beta }}(\partial _{\alpha {\beta }}^2f-\Gamma ^{\gamma }_{\alpha {\beta }}\partial _{\gamma } f)\\&= \ g^{\alpha {\beta }}[\partial _{\alpha {\beta }}^2f-g^{\gamma \sigma }(\partial _{\alpha {\beta }}^2F\cdot \partial _{\sigma } F)\partial _{\gamma } f], \end{aligned}$$

for any twice differentiable function $f:\Sigma \rightarrow {{\mathbb {R}}}$. The curvature tensor *R* on the Riemannian manifold $(\Sigma ,g)$ is given by

$$\begin{aligned} R(\partial _{\alpha },\partial _{{\beta }})\partial _{\gamma }&= \ (\partial _{\alpha } \Gamma _{{\beta }\gamma }^{\sigma } +\Gamma _{{\beta }\gamma }^m\Gamma _{\alpha m}^{\sigma } -\partial _{{\beta }} \Gamma _{\alpha \gamma }^{\sigma } -\Gamma _{\alpha \gamma }^m\Gamma _{{\beta }m}^{\sigma } )\partial _{\sigma }. \end{aligned}$$

Hence, we have

2.2 $$\begin{aligned} R_{\gamma \alpha {\beta }}^{\sigma }=\partial _{\alpha } \Gamma _{{\beta }\gamma }^{\sigma }-\partial _{{\beta }} \Gamma _{\alpha \gamma }^{\sigma } +\Gamma _{{\beta }\gamma }^m\Gamma _{\alpha m}^{\sigma } -\Gamma _{\alpha \gamma }^m\Gamma _{{\beta }m}^{\sigma }. \end{aligned}$$

By $R(X,Y,Z,W)=\langle R(Z,W)Y,X\rangle$ and $R_{\alpha {\beta }\gamma \sigma }=R(\partial _{\alpha },\partial _{{\beta }},\partial _{\gamma },\partial _{\sigma })$, we get

$$\begin{aligned} R_{\alpha {\beta }\gamma \sigma }=\langle R(\partial _{\gamma },\partial _{\sigma })\partial _{{\beta }},\partial _{\alpha }\rangle =\langle R_{{\beta }\gamma \sigma }^{\mu } \partial _{\mu },\partial _{\alpha }\rangle =g_{\mu \alpha }R_{{\beta }\gamma \sigma }^{\mu }, \end{aligned}$$

We will also use the Ricci curvature

$$\begin{aligned} {{\,\mathrm{Ric}\,}}_{\alpha {\beta }}=R^{\sigma }_{\alpha \sigma {\beta }}=g^{\sigma \gamma }R_{\gamma \alpha \sigma {\beta }}. \end{aligned}$$

### The second fundamental form

Let ${\bar{\nabla }}$ be the Levi-Civita connection in $({{\mathbb {R}}}^{d+2},g_{{{\mathbb {R}}}^{d+2}})$ and let ${\mathbf {h}}$ be the second fundamental form for $\Sigma$ as an embedded manifold. For any vector fields $u,v\in T_{*}\Sigma$, the Gauss relation is

$$\begin{aligned} {\bar{\nabla }}_u F_{*}v=F_{*}(\nabla _u v)+ {\mathbf {h}}(u,v). \end{aligned}$$

Then we have

$$\begin{aligned} {\mathbf {h}}_{\alpha {\beta }}&= {\mathbf {h}}(\partial _{\alpha },\partial _{{\beta }})={\bar{\nabla }}_{\partial _{\alpha }} \partial _{{\beta }} F-F_{*}(\nabla _{\partial _{\alpha }}\partial _{{\beta }})\\&= \partial _{\alpha {\beta }}^2 F+{\bar{\Gamma }}_{kl}\partial _{\alpha } F^k \partial _{{\beta }} F^l-\Gamma _{\alpha {\beta }}^{\gamma } \partial _{\gamma } F. \end{aligned}$$

By ${\bar{\Gamma }}_{kl}^j=0$, this gives the mean curvature ${\mathbf {H}}$ at *F*(*x*),

$$\begin{aligned} {\mathbf {H}}={\text {tr}}_g {\mathbf {h}}=g^{\alpha {\beta }}{\mathbf {h}}_{\alpha {\beta }}=g^{\alpha {\beta }}(\partial ^2_{\alpha {\beta }}F-\Gamma ^{\gamma }_{\alpha {\beta }}\partial _{\gamma } F)=\Delta _g F. \end{aligned}$$

Hence, the *F*-equation in (1.1) is rewritten as

$$\begin{aligned} (\partial _t F)^{\perp }=J(F)\Delta _g F=J(F)g^{\alpha {\beta }}(\partial ^2_{\alpha {\beta }}F-\Gamma ^{\gamma }_{\alpha {\beta }}\partial _{\gamma } F). \end{aligned}$$

This equation is still independent of the choice of coordinates in $\Sigma ^d$, which at this point are allowed to fully depend on *t*.

### The complex structure equations

Here we introduce a complex structure on the normal bundle $N\Sigma _t$. This is achieved by choosing $\{\nu _1,\nu _2\}$ to be an orthonormal basis of $N\Sigma _t$ such that

$$\begin{aligned} J\nu _1=\nu _2,\quad J\nu _2=-\nu _1. \end{aligned}$$

Such a choice is not unique; in making it we introduce a second component to our gauge group, namely the group of sections of an *SU*(1) bundle over $I \times {{\mathbb {R}}}^d$.

The vectors $\{ F_1,\ldots ,F_d,\nu _1,\nu _2\}$ form a frame at each point on the manifold $(\Sigma ,g)$, where $F_{\alpha }$ for $\alpha \in \{1,\ldots ,d\}$ are defined as

$$\begin{aligned} F_{\alpha }=\partial _{\alpha }F. \end{aligned}$$

If we differentiate the frame, we obtain a set of structure equations of the following type

2.3 $$\begin{aligned} \left\{ \begin{aligned}&\partial _{\alpha } F_{{\beta }}=\Gamma ^{\gamma }_{\alpha {\beta }}F_{\gamma }+\kappa _{\alpha {\beta }}\nu _1+\tau _{\alpha {\beta }}\nu _2,\\&\partial _{\alpha } \nu _1=-\kappa ^{\gamma }_{\alpha } F_{\gamma }+A_{\alpha } \nu _2,\\&\partial _{\alpha } \nu _2=-\tau ^{\gamma }_{\alpha } F_{\gamma }-A_{\alpha } \nu _1, \end{aligned}\right. \end{aligned}$$

where the tensors $\kappa _{\alpha {\beta }},\tau _{\alpha {\beta }}$ and the connection coefficients $A_{\alpha }$ are defined by

$$\begin{aligned} \kappa _{\alpha {\beta }}\,{:}{=}\,\partial _{\alpha } F_{{\beta }}\cdot \nu _1,\quad \tau _{\alpha {\beta }}\,{:}{=}\,\partial _{\alpha } F_{{\beta }}\cdot \nu _2,\quad A_{\alpha }=\partial _{\alpha }\nu _1\cdot \nu _2. \end{aligned}$$

The mean curvature ${\mathbf {H}}$ can be expressed in term of $\kappa _{\alpha {\beta }}$ and $\tau _{\alpha {\beta }}$, i.e.

$$\begin{aligned} {\mathbf {H}}=g^{\alpha {\beta }}(\kappa _{\alpha {\beta }}\nu _1+\tau _{\alpha {\beta }}\nu _2). \end{aligned}$$

Next, we complexify the structure equations (2.3) as follows. We define the complex vector *m* and the complex second fundamental form tensor $\lambda _{\alpha {\beta }}$ to be

$$\begin{aligned} m=\nu _1+i\nu _2,\quad \lambda _{\alpha {\beta }}=\kappa _{\alpha {\beta }}+i\tau _{\alpha {\beta }}. \end{aligned}$$

Then we define the *complex scalar mean curvature* $\psi$ as the trace of the second fundamental form,

2.4 $$\begin{aligned} \psi \,{:}{=}\,{\text {tr}}\lambda =g^{\alpha {\beta }}\lambda _{\alpha {\beta }}. \end{aligned}$$

Our objective for the rest of this section will be to interpret the (SMCF) equation as a nonlinear Schrödinger evolution for $\psi$, by making suitable gauge choices.

We remark that the action of sections of the *SU*(1) bundle is given by

2.5 $$\begin{aligned} \psi \rightarrow e^{i \theta }\psi , \quad \lambda \rightarrow e^{i \theta }\lambda , \quad m \rightarrow e^{i\theta } m, \quad A_\alpha \rightarrow A_\alpha - \partial _\alpha \theta . \end{aligned}$$

for a real valued function $\theta$.

We use the convention for the inner product of two complex vectors, say *a* and *b*, as

$$\begin{aligned} \langle a,b\rangle =\sum _{j=1}^{d+2}a_{j} {\bar{b}}_{j}, \end{aligned}$$

where $a_{j}$ and $b_{j}$ are the complex components of *a* and *b* respectively. Then we get the following relations for the complex vector *m*,

$$\begin{aligned} \langle m,m\rangle =|\nu _1|^2+|\nu _2|^2=2,\quad \langle m,{\bar{m}}\rangle =\langle {\bar{m}},m\rangle =|\nu _1|^2-|\nu _2|^2=0. \end{aligned}$$

From these relations we obtain

$$\begin{aligned} \kappa _{\alpha {\beta }}\nu _1+\tau _{\alpha {\beta }}\nu _2&= \frac{1}{2}(\lambda _{\alpha {\beta }}+{\bar{\lambda }}_{\alpha {\beta }})\frac{1}{2}(m+{\bar{m}})+\frac{1}{2i}(\lambda _{\alpha {\beta }}-{\bar{\lambda }}_{\alpha {\beta }})\frac{1}{2i}(m-{\bar{m}})\\&= \frac{1}{2}(\lambda _{\alpha {\beta }}{\bar{m}}+{\bar{\lambda }}_{\alpha {\beta }}m) =\mathop {\mathrm{Re}}\nolimits (\lambda _{\alpha {\beta }}{\bar{m}}). \end{aligned}$$

Then the structure equations (2.3) are rewritten as

2.6 $$\begin{aligned} \left\{ \begin{aligned}&\partial _{\alpha }F_{{\beta }}=\Gamma ^{\gamma }_{\alpha {\beta }}F_{\gamma }+\mathop {\mathrm{Re}}\nolimits (\lambda _{\alpha {\beta }}{\bar{m}}),\\&\partial _{\alpha }^A m=-\lambda ^{\gamma }_{\alpha } F_{\gamma }, \end{aligned}\right. \end{aligned}$$

where

$$\begin{aligned} \partial _{\alpha }^A=\partial _{\alpha }+iA_{\alpha }. \end{aligned}$$

### The Gauss and Codazzi relations

The Gauss and Codazzi equations are derived from the equality of second derivatives $\partial _{\alpha }\partial _{{\beta }}F_{\gamma }=\partial _{{\beta }}\partial _{\alpha }F_{\gamma }$ for the tangent vectors on the submanifold $\Sigma$ and for the normal vectors respectively. Here we use the Gauss and Codazzi relations to derive the Riemannian curvature, the first compatibility condition and a symmetry.

By the structure equations (2.6), we get

2.7 $$\begin{aligned} \partial ^2_{\alpha {\beta }}F_{\gamma }= & {} \partial _{\alpha }(\Gamma ^{\sigma }_{{\beta }\gamma }F_{\sigma }+\mathop {\mathrm{Re}}\nolimits (\lambda _{{\beta }\gamma }{\bar{m}}))\nonumber \\= & {} \partial _{\alpha }\Gamma _{{\beta }\gamma }^{\sigma } F_{\sigma }+\Gamma _{{\beta }\gamma }^{\sigma } (\Gamma ^{\mu }_{\alpha \sigma }F_{\mu }+\mathop {\mathrm{Re}}\nolimits (\lambda _{\alpha \sigma }{\bar{m}}))+\mathop {\mathrm{Re}}\nolimits (\partial _{\alpha }\lambda _{{\beta }\gamma }{\bar{m}}\nonumber \\&+\lambda _{{\beta }\gamma }(iA_{\alpha }{\bar{m}}-{\bar{\lambda }}_{\alpha }^{\mu }F_{\mu }))\nonumber \\= & {} (\partial _{\alpha }\Gamma _{{\beta }\gamma }^{\sigma }+\Gamma _{{\beta }\gamma }^{\mu }\Gamma ^{\sigma }_{\alpha \mu }-\mathop {\mathrm{Re}}\nolimits (\lambda _{{\beta }\gamma }{\bar{\lambda }}_{\alpha }^{\sigma }))F_{\sigma }+\mathop {\mathrm{Re}}\nolimits [(\partial _{\alpha }^A\lambda _{{\beta }\gamma }+\Gamma ^{\sigma }_{{\beta }\gamma }\lambda _{\alpha \sigma }){\bar{m}}].\qquad \end{aligned}$$

Then in view of $\partial _{\alpha }\partial _{{\beta }} F_{\gamma }=\partial _{{\beta }}\partial _{\alpha } F_{\gamma }$ and equating the coefficients of the tangent vectors, we obtain

$$\begin{aligned} \partial _{\alpha }\Gamma _{{\beta }\gamma }^{\sigma }+\Gamma _{{\beta }\gamma }^{\mu }\Gamma ^{\sigma }_{\alpha \mu }-\partial _{{\beta }}\Gamma _{\alpha \gamma }^{\sigma }-\Gamma _{\alpha \gamma }^{\mu }\Gamma ^{\sigma }_{{\beta }\mu }=\mathop {\mathrm{Re}}\nolimits (\lambda _{{\beta }\gamma }{\bar{\lambda }}_{\alpha }^{\sigma }-\lambda _{\alpha \gamma }{\bar{\lambda }}_{{\beta }}^{\sigma }). \end{aligned}$$

This gives the Riemannian curvature

2.8 $$\begin{aligned} R_{\sigma \gamma \alpha {\beta }}=\langle R^{\mu }_{\gamma \alpha {\beta }}F_{\mu },F_{\sigma }\rangle =\langle R(\partial _{\alpha },\partial _{{\beta }})F_{\gamma },F_{\sigma }\rangle =\mathop {\mathrm{Re}}\nolimits (\lambda _{{\beta }\gamma }{\bar{\lambda }}_{\alpha \sigma }-\lambda _{\alpha \gamma }{\bar{\lambda }}_{{\beta }\sigma }), \end{aligned}$$

which is a complex formulation of the Gauss equation. Correspondingly we obtain the the Ricci curvature

2.9 $$\begin{aligned} {{\,\mathrm{Ric}\,}}_{\gamma {\beta }}=\mathop {\mathrm{Re}}\nolimits (\lambda _{\gamma {\beta }}{\bar{\psi }}-\lambda _{\gamma \alpha }{\bar{\lambda }}_{{\beta }}^{\alpha }). \end{aligned}$$

After equating the coefficients of the vector *m* in (2.7), we obtain

$$\begin{aligned} \partial ^A_{\alpha }\lambda _{{\beta }\gamma }+\Gamma _{{\beta }\gamma }^{\sigma }\lambda _{\alpha \sigma }=\partial ^A_{{\beta }}\lambda _{\alpha \gamma }+\Gamma _{\alpha \gamma }^{\sigma }\lambda _{{\beta }\sigma }, \end{aligned}$$

By the definition of covariant derivatives, i.e.

$$\begin{aligned} \nabla _{\alpha } \lambda _{{\beta }\gamma }=\partial _{\alpha }\lambda _{{\beta }\gamma }-\Gamma _{\alpha {\beta }}^{\sigma } \lambda _{\sigma \gamma }-\Gamma _{\alpha \gamma }^{\sigma }\lambda _{{\beta }\sigma }, \end{aligned}$$

we obtain

$$\begin{aligned} \partial ^A_{\alpha }\lambda _{{\beta }\gamma }-\Gamma _{\alpha \gamma }^{\sigma }\lambda _{{\beta }\sigma }-\Gamma _{\alpha {\beta }}^{\sigma }\lambda _{\sigma \gamma }=\partial ^A_{{\beta }}\lambda _{\alpha \gamma }-\Gamma _{{\beta }\gamma }^{\sigma }\lambda _{\alpha \sigma }-\Gamma _{\alpha {\beta }}^{\sigma }\lambda _{\sigma \gamma }. \end{aligned}$$

This implies the complex formulation of the Codazzi equation, namely

2.10 $$\begin{aligned} \nabla ^A_{\alpha } \lambda _{{\beta }\gamma }=\nabla ^A_{{\beta }}\lambda _{\alpha \gamma }. \end{aligned}$$

As a consequence of this equality, we obtain

#### Lemma 2.1

The second fundamental form $\lambda$ satisfies the Codazzi relations

2.11 $$\begin{aligned} \nabla ^A_{\alpha } \lambda ^{\gamma }_{{\beta }}=\nabla ^A_{{\beta }} \lambda ^{\gamma }_{\alpha }=\nabla ^{A,\gamma }\lambda _{\alpha {\beta }}. \end{aligned}$$

#### Proof

Here we prove the last equality. By $\nabla _{{\beta }}g^{\gamma \sigma }=0$ and (2.10) we have

$$\begin{aligned} \nabla ^A_{{\beta }}\lambda ^{\gamma }_{\alpha }=g^{\gamma \sigma }\nabla _{{\beta }}^A \lambda _{\sigma \alpha }=g^{\gamma \sigma }\nabla ^A_{\sigma }\lambda _{{\beta }\alpha }=\nabla ^{A,\gamma }\lambda _{\alpha {\beta }}. \end{aligned}$$

The first equality can be proved similarly. $\square$

Next, we use the relation $\partial _{\alpha }\partial _{{\beta }}m=\partial _{{\beta }}\partial _{\alpha }m$ in order to derive a compatibility condition between the connection *A* in the normal bundle and the second fundamental form. Indeed, from $\partial _{\alpha }\partial _{{\beta }}m=\partial _{{\beta }}\partial _{\alpha }m$ we obtain the commutation relation

2.12 $$\begin{aligned}{}[\partial ^A_{\alpha },\partial ^A_{{\beta }}]m=i(\partial _{\alpha } A_{{\beta }}-\partial _{{\beta }} A_{\alpha })m. \end{aligned}$$

By (2.6) we have

$$\begin{aligned} \partial ^A_{\alpha }\partial ^A_{{\beta }} m&= -\partial ^A_{\alpha }(\lambda ^{\gamma }_{{\beta }} F_{\gamma })=-(\partial ^A_{\alpha }\lambda ^{\sigma }_{{\beta }}+\lambda ^{\gamma }_{{\beta }}\Gamma ^{\sigma }_{\alpha \gamma })F_{\sigma }-\lambda ^{\gamma }_{{\beta }}\mathop {\mathrm{Re}}\nolimits (\lambda _{\alpha \gamma }{\bar{m}}). \end{aligned}$$

Then multiplying (2.12) by *m* yields

$$\begin{aligned} 2i(\partial _{\alpha } A_{{\beta }}-\partial _{{\beta }} A_{\alpha })&= \langle [-\lambda ^{\gamma }_{{\beta }}\mathop {\mathrm{Re}}\nolimits (\lambda _{\alpha \gamma }{\bar{m}})+\lambda ^{\gamma }_{\alpha }\mathop {\mathrm{Re}}\nolimits (\lambda _{{\beta }\gamma }{\bar{m}})],m\rangle \\&= -\lambda ^{\gamma }_{{\beta }} {\bar{\lambda }}_{\alpha \gamma }+\lambda ^{\gamma }_{\alpha }{\bar{\lambda }}_{{\beta }\gamma }=2i\mathop {\mathrm{Im}}\nolimits (\lambda ^{\gamma }_{\alpha }{\bar{\lambda }}_{{\beta }\gamma }). \end{aligned}$$

This gives the compatibility condition for the curvature of *A*,

$$\begin{aligned} \partial _{\alpha } A_{{\beta }}-\partial _{{\beta }} A_{\alpha }=\mathop {\mathrm{Im}}\nolimits (\lambda ^{\gamma }_{\alpha }{\bar{\lambda }}_{{\beta }\gamma }). \end{aligned}$$

Using covariant derivative, this can be written as

2.13 $$\begin{aligned} \nabla _{\alpha } A_{{\beta }}-\nabla _{{\beta }} A_{\alpha }=\mathop {\mathrm{Im}}\nolimits (\lambda ^{\gamma }_{\alpha }{\bar{\lambda }}_{{\beta }\gamma }), \end{aligned}$$

which can be seen as the complex form of the Ricci equations.

We remark that, by equating the coefficients of the tangent vectors in (2.12), we also obtain

$$\begin{aligned} \partial ^A_{\alpha }\lambda ^{\sigma }_{{\beta }}+\lambda ^{\gamma }_{{\beta }}\Gamma ^{\sigma }_{\alpha \gamma }=\partial ^A_{{\beta }}\lambda ^{\sigma }_{\alpha }+\lambda ^{\gamma }_{\alpha }\Gamma ^{\sigma }_{{\beta }\gamma }, \end{aligned}$$

and hence

$$\begin{aligned} \nabla ^A_{\alpha }\lambda ^{\sigma }_{{\beta }}=\nabla ^A_{{\beta }}\lambda ^{\sigma }_{\alpha }, \end{aligned}$$

which is the same as (2.11).

Next, we state an elliptic system for the second fundamental form $\lambda _{\alpha {\beta }}$ in terms of $\psi$, using the Codazzi relations (2.11).

#### Lemma 2.2

(Div-curl system for $\lambda$). The second fundamental form $\lambda$ satisfies

2.14 $$\begin{aligned} \left\{ \begin{aligned}&\nabla ^A_\alpha \lambda _{{\beta }\gamma }-\nabla ^A_{\beta }\lambda _{\alpha \gamma }=0,\\&\nabla ^{A,\alpha }\lambda _{\alpha {\beta }}=\nabla ^A_{\beta }\psi . \end{aligned}\right. \end{aligned}$$

We remark that a-priori solutions $\lambda$ to the above system are not guaranteed to be symmetric, so we record this as a separate property:

2.15 $$\begin{aligned} \lambda _{\alpha {\beta }} = \lambda _{{\beta }\alpha }. \end{aligned}$$

Finally, we turn our attention to the connection *A*, for which we have the curvature relations (2.13) together with the gauge group (2.5). In order to both fix the gauge and obtain an elliptic system for *A*, we impose the Coulomb gauge condition

2.16 $$\begin{aligned} \nabla ^\alpha A_\alpha =0. \end{aligned}$$

Next, we derive the elliptic *A*-equations from the Ricci equations (2.13).

#### Lemma 2.3

(Elliptic equations for *A*). Under the Coulomb gauge condition, the connection *A* solves

2.17 $$\begin{aligned} \nabla ^{\gamma }\nabla _{\gamma } A_{\alpha }=\mathop {\mathrm{Re}}\nolimits (\lambda _\alpha ^\sigma {\bar{\psi }}-\lambda _{\alpha }^{\gamma }{\bar{\lambda }}_{\gamma }^{\sigma })A_{\sigma }+\nabla ^{\gamma }\mathop {\mathrm{Im}}\nolimits (\lambda ^{\sigma }_{\gamma }{\bar{\lambda }}_{\alpha \sigma }). \end{aligned}$$

#### Proof

Applying $\nabla ^{{\beta }}$ to (2.13), by curvature and (2.16) we obtain

$$\begin{aligned} \nabla ^{{\beta }}\nabla _{{\beta }} A_{\alpha }={{\,\mathrm{Ric}\,}}_{\alpha \delta }A^{\delta }+\nabla ^{{\beta }}\mathop {\mathrm{Im}}\nolimits (\lambda ^{\sigma }_{{\beta }}{\bar{\lambda }}_{\alpha \sigma }). \end{aligned}$$

Then the equation (2.17) for *A* is obtained from (2.9). $\square$

### The elliptic equation for the metric *g* in harmonic coordinates

Here we take the next step towards fixing the gauge, by choosing to work in harmonic coordinates. Precisely, we will require the coordinate functions $\{x_{\alpha },\alpha =1,\ldots ,d\}$ to be globally Lipschitz solutions of the elliptic equations

2.18 $$\begin{aligned} \Delta _g x_{\alpha }=0. \end{aligned}$$

This determines the coordinates uniquely modulo time dependent affine transformations. This remaining ambiguity will be removed later on by imposing suitable boundary conditions at infinity. After this, the only remaining degrees of freedom in the choice of coordinates will be given by *time independent* translations and rigid rotations. Thus, once a choice is made at the initial time, the coordinates will be uniquely determined later on (see also Remark 2.5.1).

Here we will interpret the above harmonic coordinate condition at fixed time as an elliptic equation for the metric *g* (see e.g. [10, 30, P161]). The equations (2.18) may be expressed in terms of the Christoffel symbols $\Gamma$, which must satisfy the condition

2.19 $$\begin{aligned} g^{\alpha {\beta }}\Gamma ^{\gamma }_{\alpha {\beta }}=0,\quad \mathrm{for}\ \gamma =1,\ldots ,d. \end{aligned}$$

This implies

2.20 $$\begin{aligned} g^{\alpha {\beta }}\partial _{\alpha } g_{{\beta }\gamma }=\frac{1}{2}g^{\alpha {\beta }}\partial _{\gamma } g_{\alpha {\beta }},\quad \partial _{\alpha } g^{\alpha \gamma }=\frac{1}{2}g_{\alpha {\beta }}g^{\gamma \sigma }\partial _{\sigma } g^{\alpha {\beta }}. \end{aligned}$$

Let

2.21 $$\begin{aligned} \Gamma _{\alpha {\beta },\gamma }=\frac{1}{2}(\partial _{\alpha } g_{{\beta }\gamma }+\partial _{{\beta }} g_{\alpha \gamma }-\partial _{\gamma } g_{\alpha {\beta }})=g_{\gamma \sigma }\Gamma ^{\sigma }_{\alpha {\beta }}. \end{aligned}$$

Then we also have

$$\begin{aligned} g^{\alpha {\beta }}\Gamma _{\alpha {\beta },\gamma }=g^{\alpha {\beta }}g_{\gamma \sigma }\Gamma ^{\sigma }_{\alpha {\beta }}=0, \end{aligned}$$

and

$$\begin{aligned} R_{\alpha \gamma {\beta }\sigma }=\partial _{{\beta }} \Gamma _{\gamma \sigma ,\alpha }-\partial _{\sigma }\Gamma _{{\beta }\gamma ,\alpha }+\Gamma _{\sigma \alpha ,\nu }\Gamma ^{\nu }_{{\beta }\gamma }-\Gamma _{{\beta }\alpha ,\nu }\Gamma ^{\nu }_{\gamma \sigma }. \end{aligned}$$

This leads to an equation for the metric *g*:

#### Lemma 2.4

(Elliptic equations of *g*). In harmonic coordinates, the metric *g* satisfies

2.22 $$\begin{aligned} \begin{aligned} g^{\alpha {\beta }}\partial ^2_{\alpha {\beta }}g_{\gamma \sigma }&= \ [-\partial _{\gamma } g^{\alpha {\beta }}\partial _{{\beta }} g_{\alpha \sigma }-\partial _{\sigma } g^{\alpha {\beta }}\partial _{{\beta }} g_{\alpha \gamma }+\partial _{\gamma } g_{\alpha {\beta }}\partial _{\sigma } g^{\alpha {\beta }}]\\&\quad +2g^{\alpha {\beta }}\Gamma _{\sigma \alpha ,\nu }\Gamma ^{\nu }_{{\beta }\gamma }-2\mathop {\mathrm{Re}}\nolimits (\lambda _{\gamma \sigma }{\bar{\psi }}-\lambda _{\alpha \gamma }{\bar{\lambda }}_{\sigma }^{\alpha }). \end{aligned} \end{aligned}$$

#### Proof

By the definition of Ricci curvature, (2.2) and (2.19), we have

$$\begin{aligned} {{\,\mathrm{Ric}\,}}_{\gamma \sigma }&= g^{\alpha {\beta }}R_{\alpha \gamma {\beta }\sigma }=g^{\alpha {\beta }}(\partial _{{\beta }} \Gamma _{\gamma \sigma ,\alpha }-\partial _{\sigma }\Gamma _{{\beta }\gamma ,\alpha })+g^{\alpha {\beta }}\Gamma _{\sigma \alpha ,\nu }\Gamma ^{\nu }_{{\beta }\gamma }-g^{\alpha {\beta }}\Gamma _{{\beta }\alpha ,\nu }\Gamma ^{\nu }_{\gamma \sigma }\\&= g^{\alpha {\beta }}(\partial _{{\beta }} \Gamma _{\gamma \sigma ,\alpha }-\partial _{\sigma }\Gamma _{{\beta }\gamma ,\alpha })+g^{\alpha {\beta }}\Gamma _{\sigma \alpha ,\nu }\Gamma ^{\nu }_{{\beta }\gamma }\\&= I+II. \end{aligned}$$

We compute the first term *I*. By the definition of $\Gamma _{\alpha {\beta },\gamma }$ in (2.21), we have

$$\begin{aligned} I&= \frac{1}{2}g^{\alpha {\beta }}[\partial _{{\beta }}(\partial _{\gamma } g_{\sigma \alpha }+\partial _{\sigma } g_{\gamma \alpha }-\partial _{\alpha } g_{\gamma \sigma })-\partial _{\sigma }(\partial _{{\beta }} g_{\gamma \alpha }+\partial _{\gamma }g_{{\beta }\alpha }-\partial _{\alpha } g_{{\beta }\gamma })]\\&= -\frac{1}{2}g^{\alpha {\beta }}\partial ^2_{\alpha {\beta }}g_{\gamma \sigma }+\frac{1}{2}g^{\alpha {\beta }}(\partial ^2_{{\beta }\gamma }g_{\alpha \sigma }+\partial ^2_{\alpha \sigma }g_{{\beta }\gamma }-\partial ^2_{\gamma \sigma }g_{\alpha {\beta }}) \end{aligned}$$

Since, by (2.20) we have

$$\begin{aligned} g^{\alpha {\beta }}(\partial ^2_{\gamma {\beta }}g_{\alpha \sigma }-\frac{1}{2}\partial ^2_{\gamma \sigma }g_{\alpha {\beta }})=-\partial _{\gamma } g^{\alpha {\beta }}(\partial _{{\beta }} g_{\alpha \sigma }-\frac{1}{2}\partial _{\sigma } g_{\alpha {\beta }}). \end{aligned}$$

Then

$$\begin{aligned} I&= -\frac{1}{2}g^{\alpha {\beta }}\partial ^2_{\alpha {\beta }}g_{\gamma \sigma }+\frac{1}{2}[-\partial _{\gamma } g^{\alpha {\beta }}(\partial _{{\beta }} g_{\alpha \sigma }-\frac{1}{2}\partial _{\sigma } g_{\alpha {\beta }})-\partial _{\sigma } g^{\alpha {\beta }}(\partial _{{\beta }} g_{\alpha \gamma }-\frac{1}{2}\partial _{\gamma } g_{\alpha {\beta }})]\\&= -\frac{1}{2}g^{\alpha {\beta }}\partial ^2_{\alpha {\beta }}g_{\gamma \sigma }+\frac{1}{2}[-\partial _{\gamma } g^{\alpha {\beta }}\partial _{{\beta }} g_{\alpha \sigma }-\partial _{\sigma } g^{\alpha {\beta }}\partial _{{\beta }} g_{\alpha \gamma }+\partial _{\gamma } g_{\alpha {\beta }}\partial _{\sigma } g^{\alpha {\beta }}]. \end{aligned}$$

Hence,

$$\begin{aligned} {{\,\mathrm{Ric}\,}}_{\gamma \sigma }=-\frac{1}{2}g^{\alpha {\beta }}\partial ^2_{\alpha {\beta }}g_{\gamma \sigma }+\frac{1}{2}[-\partial _{\gamma } g^{\alpha {\beta }}\partial _{{\beta }} g_{\alpha \sigma }-\partial _{\sigma } g^{\alpha {\beta }}\partial _{{\beta }} g_{\alpha \gamma }+\partial _{\gamma } g_{\alpha {\beta }}\partial _{\sigma } g^{\alpha {\beta }}]+g^{\alpha {\beta }}\Gamma _{\sigma \alpha ,\nu }\Gamma ^{\nu }_{{\beta }\gamma }. \end{aligned}$$

By (2.9) this concludes the proof of the Lemma. $\square$

### The motion of the frame $\{F_1,\ldots ,F_d,m\}$ under (SMCF)

Here we derive the equations of motion for the frame, assuming that the immersion *F* satisfying (1.1).

We begin by rewriting the SMCF equations in the form

2.23 $$\begin{aligned} \partial _t F=J(F){\mathbf {H}}(F)+V^{\gamma } F_{\gamma }, \end{aligned}$$

where $V^{\gamma }$ is a vector field on the manifold $\Sigma$, which in general depends on the choice of coordinates.

By the definition of *m* and $\lambda _{\alpha {\beta }}$, we get

$$\begin{aligned} J(F){\mathbf {H}}(F)=J(F) \mathop {\mathrm{Re}}\nolimits (\psi {\bar{m}})=\mathop {\mathrm{Re}}\nolimits i(\psi {\bar{m}})=-\mathop {\mathrm{Im}}\nolimits (\psi {\bar{m}}). \end{aligned}$$

Hence, the above *F*-equation (2.23) is rewritten as

2.24 $$\begin{aligned} \partial _t F=-\mathop {\mathrm{Im}}\nolimits (\psi {\bar{m}})+V^{\gamma } F_{\gamma }. \end{aligned}$$

Then we use this to derive the equations of motion for the frame. Applying $\partial _{\alpha }$ to (2.24), by the structure equations (2.6) we obtain

$$\begin{aligned} \partial _t F_{\alpha }&= \ \partial _{\alpha } F_t=\partial _{\alpha }[-\mathop {\mathrm{Im}}\nolimits (\psi {\bar{m}})+V^{\gamma } F_{\gamma }]\\&= -\mathop {\mathrm{Im}}\nolimits ((\partial _{\alpha }+iA_{\alpha })\psi {\bar{m}}+\psi \overline{(\partial _{\alpha }+iA_{\alpha })}{\bar{m}})+\partial _{\alpha } V^{\gamma } F_{\gamma }+V^{\gamma } (\Gamma ^{\sigma }_{\alpha \gamma }F_{\sigma }+\mathop {\mathrm{Re}}\nolimits (\lambda _{\alpha \gamma }{\bar{m}}))\\&= -\mathop {\mathrm{Im}}\nolimits (\partial ^A_{\alpha } \psi {\bar{m}}-\psi {\bar{\lambda }}^{\gamma }_{\alpha } F_{\gamma })+\partial _{\alpha } V^{\gamma } F_{\gamma }+V^{\gamma } (\Gamma ^{\sigma }_{\alpha \gamma }F_{\sigma }+\mathop {\mathrm{Re}}\nolimits (\lambda _{\alpha \gamma }{\bar{m}}))\\&= -\mathop {\mathrm{Im}}\nolimits (\partial ^A_{\alpha } \psi {\bar{m}})+\mathop {\mathrm{Re}}\nolimits (\lambda _{\alpha \gamma }V^{\gamma } {\bar{m}})+[\mathop {\mathrm{Im}}\nolimits (\psi {\bar{\lambda }}^{\gamma }_{\alpha })+\nabla _{\alpha } V^{\gamma }]F_{\gamma }\\&= -\mathop {\mathrm{Im}}\nolimits (\partial ^A_{\alpha } \psi {\bar{m}}-i\lambda _{\alpha \gamma }V^{\gamma } {\bar{m}})+[\mathop {\mathrm{Im}}\nolimits (\psi {\bar{\lambda }}^{\gamma }_{\alpha })+\nabla _{\alpha } V^{\gamma } ]F_{\gamma }. \end{aligned}$$

By the orthogonality relation $m\bot F_{\alpha }=0$, this implies

$$\begin{aligned} \langle \partial _t m,F_{\alpha }\rangle&= \ \partial _t\langle m,F_{\alpha }\rangle -\langle m,\partial _t F_{\alpha }\rangle \\&= \ -\langle m, -\mathop {\mathrm{Im}}\nolimits (\partial ^A_{\alpha } \psi {\bar{m}}-i\lambda _{\alpha \gamma }V^{\gamma } {\bar{m}})\rangle \\&= \ \langle m,\frac{i}{2}(\overline{\partial ^A_{\alpha } \psi -i\lambda _{\alpha \gamma }V^{\gamma }})m\rangle \\&= \ -i(\partial ^A_{\alpha } \psi -i\lambda _{\alpha \gamma }V^{\gamma } ). \end{aligned}$$

In order to describe the normal component of the time derivative of *m*, we also need the temporal component of the connection in the normal bundle. This is defined by

$$\begin{aligned} B=\langle \partial _t \nu _1,\nu _2\rangle . \end{aligned}$$

We have

$$\begin{aligned} (\partial _t m)^{\perp }=(\partial _t(\nu _1+i\nu _2))^{\perp }=B\nu _2-iB\nu _1=-iB(\nu _1+i\nu _2)=-iB m. \end{aligned}$$

Then we get

$$\begin{aligned} \partial _t m=-i(\partial ^{A,\alpha } \psi -i\lambda ^{\alpha }_{\gamma }V^{\gamma } )F_{\alpha }-iB m, \end{aligned}$$

which can be further rewritten as

$$\begin{aligned} \partial ^{B}_t m=-i(\partial ^{A,\alpha } \psi -i\lambda ^{\alpha }_{\gamma }V^{\gamma } )F_{\alpha }. \end{aligned}$$

Therefore, we obtain the following equations of motion for the frame

2.25 $$\begin{aligned} \left\{ \begin{aligned}&\partial _t F_{\alpha }=-\mathop {\mathrm{Im}}\nolimits (\partial ^A_{\alpha } \psi {\bar{m}}-i\lambda _{\alpha \gamma }V^{\gamma } {\bar{m}})+[\mathop {\mathrm{Im}}\nolimits (\psi {\bar{\lambda }}^{\gamma }_{\alpha })+\nabla _{\alpha } V^{\gamma }]F_{\gamma },\\&\partial ^{B}_t m=-i(\partial ^{A,\alpha } \psi -i\lambda ^{\alpha }_{\gamma }V^{\gamma } )F_{\alpha }. \end{aligned}\right. \end{aligned}$$

From this we obtain the evolution equation for the metric *g*. By the definition of the induced metric *g* (2.1) and (2.25), we have

$$\begin{aligned} \partial _t g_{\alpha {\beta }}&= \ \partial _t\langle F_{\alpha },F_{{\beta }}\rangle =\langle \partial _t F_{\alpha },F_{{\beta }}\rangle +\langle F_{\alpha },\partial _tF_{{\beta }}\rangle \\&= \ \langle -\mathop {\mathrm{Im}}\nolimits (\partial ^A_{\alpha } \psi {\bar{m}}-i\lambda _{\alpha \gamma }V^{\gamma } {\bar{m}})+[\mathop {\mathrm{Im}}\nolimits (\psi {\bar{\lambda }}^{\gamma }_{\alpha })+\nabla _{\alpha } V^{\gamma }]F_{\gamma },F_{{\beta }}\rangle \\&\quad +\langle F_{\alpha },-\mathop {\mathrm{Im}}\nolimits (\partial ^A_{{\beta }} \psi {\bar{m}}-i\lambda _{{\beta }\gamma }V^{\gamma } {\bar{m}})+[\mathop {\mathrm{Im}}\nolimits (\psi {\bar{\lambda }}^{\gamma }_{{\beta }})+\nabla _{{\beta }} V^{\gamma }]F_{\gamma }\rangle \\&= \ g_{\gamma {\beta }}(\mathop {\mathrm{Im}}\nolimits (\psi {\bar{\lambda }}^{\gamma }_{\alpha })+\nabla _{\alpha } V^{\gamma })+g_{\alpha \gamma }(\mathop {\mathrm{Im}}\nolimits (\psi {\bar{\lambda }}^{\gamma }_{{\beta }})+\nabla _{{\beta }} V^{\gamma })\\&= \ 2\mathop {\mathrm{Im}}\nolimits (\psi {\bar{\lambda }}_{\alpha {\beta }})+\nabla _{\alpha }V_{{\beta }}+\nabla _{{\beta }}V_{\alpha }, \end{aligned}$$

which we record for later reference:

2.26 $$\begin{aligned} \partial _t g_{\alpha {\beta }}=2\mathop {\mathrm{Im}}\nolimits (\psi {\bar{\lambda }}_{\alpha {\beta }})+\nabla _{\alpha }V_{{\beta }}+\nabla _{{\beta }}V_{\alpha }. \end{aligned}$$

Then we also obtain

2.27 $$\begin{aligned} \partial _t g^{\alpha {\beta }}&=-2\mathop {\mathrm{Im}}\nolimits (\psi {\bar{\lambda }}^{\alpha {\beta }})-\nabla ^{\alpha }V^{{\beta }}-\nabla ^{{\beta }}V^{\alpha }, \end{aligned}$$

2.28 $$\begin{aligned} \partial _t \Gamma _{\alpha {\beta }}^\gamma&=\nabla _\alpha G_{\beta }^\gamma +\nabla _{\beta }G_\alpha ^\gamma -\nabla ^\gamma G_{\alpha {\beta }}, \end{aligned}$$

where $G_{\alpha {\beta }}$ are defined by

2.29 $$\begin{aligned} G_{\alpha {\beta }}=\mathop {\mathrm{Im}}\nolimits (\psi {\bar{\lambda }}_{\alpha {\beta }})+\frac{1}{2}(\nabla _{\alpha }V_{{\beta }}+\nabla _{{\beta }}V_{\alpha }). \end{aligned}$$

So far, the choice of *V* has been unspecified; it depends on the choice of coordinates on our manifold as the time varies. However, once the latter is fixed via the harmonic coordinate condition (2.19), we can also derive an elliptic equation for the advection field *V*:

#### Lemma 2.5

(Elliptic equation for the vector field *V*). Under the harmonic coordinate condition (2.19), the advection field *V* solves

2.30 $$\begin{aligned} \nabla ^{\alpha }\nabla _{\alpha }V^{\gamma }=-2\nabla _\alpha \mathop {\mathrm{Im}}\nolimits (\psi {\bar{\lambda }}^{\alpha \gamma })-\mathop {\mathrm{Re}}\nolimits (\lambda ^{\gamma }_{\sigma }{\bar{\psi }}-\lambda _{\alpha \sigma }{\bar{\lambda }}^{\alpha \gamma })V^{\sigma } \ +2(\mathop {\mathrm{Im}}\nolimits (\psi {\bar{\lambda }}^{\alpha {\beta }})+\nabla ^{\alpha }V^{{\beta }})\Gamma ^{\gamma }_{\alpha {\beta }}.\nonumber \\ \end{aligned}$$

#### Proof

Applying $\partial _t$ to $g^{\alpha {\beta }}\Gamma _{\gamma {\beta }}^\gamma$, by (2.27) and (2.28) we have

$$\begin{aligned} \partial _t (g^{\alpha {\beta }}\Gamma _{\alpha {\beta }}^\gamma )&= -2G^{\alpha {\beta }}\Gamma _{\alpha {\beta }}^\gamma +g^{\alpha {\beta }}(2\nabla _\alpha G_{\beta }^\gamma -\nabla ^\gamma G_{\alpha {\beta }})\\&= -2G^{\alpha {\beta }}\Gamma _{\alpha {\beta }}^\gamma +2\nabla _\alpha \mathop {\mathrm{Im}}\nolimits (\psi {\bar{\lambda }}^{\alpha \gamma })+\Delta _g V^\gamma +[\nabla _\alpha ,\nabla ^\gamma ] V^\alpha . \end{aligned}$$

Since

$$\begin{aligned} {[}\nabla _\alpha ,\nabla ^\gamma ] V^\alpha ={{\,\mathrm{Ric}\,}}^\gamma _\sigma V^\sigma =\mathop {\mathrm{Re}}\nolimits (\lambda ^\gamma _\sigma {\bar{\psi }}-\lambda _{\alpha \sigma }{\bar{\lambda }}^{\alpha \gamma })V^\sigma . \end{aligned}$$

By the harmonic coordinate condition (2.19), the above two equalities give the *V*-equations (2.30). $\square$

#### Remark 2.5.1

Consider an arbitrary choice of coordinates (parametrization) $\{x_1,\ldots ,x_d\}$ for the time evolving manifolds $\Sigma _t$ for $t \in [0,T]$. This yields a representation of $\Sigma _t$ as the image of a map

$$\begin{aligned} F:{{\mathbb {R}}}^d\times [0,T]\rightarrow {{\mathbb {R}}}^{d+2}, \end{aligned}$$

restricted to time *t*. If $\Sigma _t$ moves along the (SMCF) flow (2.23), then we have the relation

$$\begin{aligned} \partial _t (g^{\alpha {\beta }}\Gamma _{\alpha {\beta }}^\gamma )=(V\ equation). \end{aligned}$$

Here we uniquely determine the evolution of the coordinates as the time varies by choosing the advection vector field *V*, precisely so that it satisfies the *V*-equation (2.30). For this choice we obtain $\partial _t(g^{\alpha {\beta }}\Gamma _{\alpha {\beta }}^\gamma )=0$. This implies that $g^{\alpha {\beta }}\Gamma _{\alpha {\beta }}^\gamma$ is conserved for any $x\in {{\mathbb {R}}}^d$, and thus the harmonic gauge condition is propagated in time.

### Derivation of the modified Schrödinger system from SMCF

Here we derive the main Schrödinger equation and the second compatibility condition. We consider the commutation relation

$$\begin{aligned}{}[\partial ^{B}_t,\partial ^A_{\alpha }]m=i(\partial _t A_{\alpha }-\partial _{\alpha } B)m. \end{aligned}$$

In order, for the left-hand side, by (2.6) and (2.25) we have

$$\begin{aligned} \partial ^{B}_t\partial ^A_{\alpha } m&= -\partial ^{B}_t(\lambda ^{\gamma }_{\alpha } F_{\gamma })=-\partial ^{B}_t\lambda ^{\gamma }_{\alpha } \cdot F_{\gamma }-\lambda ^{\gamma }_{\alpha }\cdot \partial _t F_{\gamma }\\&= -[\partial ^{B}_t\lambda ^{\sigma }_{\alpha }+\lambda ^{\gamma }_{\alpha }(\mathop {\mathrm{Im}}\nolimits (\psi {\bar{\lambda }}^{\sigma }_{\gamma })+\nabla _{\gamma }V^{\sigma })]F_{\sigma }+\lambda ^{\gamma }_{\alpha }\mathop {\mathrm{Im}}\nolimits (\partial ^A_{\gamma }\psi {\bar{m}}-i\lambda _{\gamma \sigma }V^{\sigma }{\bar{m}}), \end{aligned}$$

and

$$\begin{aligned} \partial ^A_{\alpha }\partial ^{B}_t m&= -i\partial ^A_{\alpha }[(\partial ^{A,\sigma } \psi -i\lambda ^{\sigma }_{\gamma }V^{\gamma } )F_{\sigma }]\\&= -i\partial ^A_{\alpha }(\partial ^{A,\sigma } \psi -i\lambda ^{\sigma }_{\gamma }V^{\gamma } )F_{\sigma }-i(\partial ^{A,\sigma } \psi -i\lambda ^{\sigma }_{\gamma }V^{\gamma } )[\Gamma ^{\mu }_{\alpha \sigma }F_{\mu }+\mathop {\mathrm{Re}}\nolimits (\lambda _{\alpha \sigma }{\bar{m}})]\\&= -i\nabla ^A_{\alpha }(\partial ^{A,\sigma } \psi -i\lambda ^{\sigma }_{\gamma }V^{\gamma } )F_{\sigma }-i(\partial ^{A,\sigma } \psi -i\lambda ^{\sigma }_{\gamma }V^{\gamma } )\mathop {\mathrm{Re}}\nolimits (\lambda _{\alpha \gamma }{\bar{m}}). \end{aligned}$$

Then by the above three equalities, equating the coefficients of the tangent vectors and the normal vector *m*, we obtain the evolution equation for $\lambda$

2.31 $$\begin{aligned} \partial ^{B}_t\lambda ^{\sigma }_{\alpha }+\lambda ^{\gamma }_{\alpha }(\mathop {\mathrm{Im}}\nolimits (\psi {\bar{\lambda }}^{\sigma }_{\gamma })+\nabla _{\gamma } V^{\sigma })=i\nabla ^A_{\alpha }(\partial ^{A,\sigma } \psi -i\lambda ^{\sigma }_{\gamma }V^{\gamma } ), \end{aligned}$$

as well as the compatibility condition (curvature relation)

$$\begin{aligned} \begin{aligned} \partial _t A_{\alpha }-\partial _{\alpha } B&= \ \frac{1}{2i}\langle \lambda ^{\gamma }_{\alpha }\mathop {\mathrm{Im}}\nolimits (\partial ^A_{\gamma }\psi {\bar{m}}-i\lambda _{\gamma \sigma }V^{\sigma }{\bar{m}})+i(\partial ^{A,\sigma } \psi -i\lambda ^{\sigma }_{\gamma }V^{\gamma } )\mathop {\mathrm{Re}}\nolimits (\lambda _{\alpha \sigma }{\bar{m}}),{\bar{m}}\rangle \\&= \ \frac{1}{2}\lambda _{\alpha }^{\gamma }({\bar{\partial }}^A_{\gamma }{\bar{\psi }}+i{\bar{\lambda }}_{\gamma \sigma }V^{\sigma })+\frac{1}{2}(\partial ^{A,\sigma } \psi -i\lambda ^{\sigma }_{\gamma }V^{\gamma } ){\bar{\lambda }}_{\alpha \sigma }\\&= \ \frac{1}{2}[\lambda _{\alpha }^{\gamma }({\bar{\partial }}^A_{\gamma }{\bar{\psi }}+i{\bar{\lambda }}_{\gamma \sigma }V^{\sigma })+{\bar{\lambda }}_{\alpha }^{\gamma }(\partial ^A_{\gamma }\psi -i\lambda _{\gamma \sigma }V^{\sigma })]\\&= \ \mathop {\mathrm{Re}}\nolimits (\lambda _{\alpha }^{\gamma }{\bar{\partial }}^A_{\gamma }{\bar{\psi }})-\mathop {\mathrm{Im}}\nolimits (\lambda ^\gamma _\alpha {\bar{\lambda }}_{\gamma \sigma })V^\sigma , \end{aligned} \end{aligned}$$

which we record for later reference:

2.32 $$\begin{aligned} \partial _t A_{\alpha }-\partial _{\alpha } B = \mathop {\mathrm{Re}}\nolimits (\lambda _{\alpha }^{\gamma }{\bar{\partial }}^A_{\gamma }{\bar{\psi }})-\mathop {\mathrm{Im}}\nolimits (\lambda ^\gamma _\alpha {\bar{\lambda }}_{\gamma \sigma })V^\sigma . \end{aligned}$$

This in turn allows us to use the Coulomb gauge condition (2.16) in order to obtain an elliptic equation for *B*:

#### Lemma 2.6

(Elliptic equation of *B*). The temporal connection coefficient *B* solves

2.33 $$\begin{aligned} \nabla ^{\gamma }\nabla _{\gamma }B=-\nabla ^{\gamma }[\mathop {\mathrm{Re}}\nolimits (\lambda _{\gamma }^{\sigma }{\bar{\partial }}^A_{\sigma }{\bar{\psi }})-\mathop {\mathrm{Im}}\nolimits (\lambda ^\sigma _\gamma {\bar{\lambda }}_{\sigma {\beta }})V^{\beta }]+(2\mathop {\mathrm{Im}}\nolimits (\psi {\bar{\lambda }}^{{\beta }\gamma })+\nabla ^{{\beta }}V^{\gamma }+\nabla ^{\gamma }V^{{\beta }})\partial _{{\beta }}A_{\gamma }.\nonumber \\ \end{aligned}$$

#### Proof

Applying $\nabla ^{\alpha }$ to (2.32) yields

$$\begin{aligned} \nabla ^{\gamma }\nabla _{\gamma } B=\nabla ^{\gamma }\partial _tA_{\gamma }-\nabla ^{\gamma }\mathop {\mathrm{Re}}\nolimits [\lambda _{\gamma }^{\sigma }({\bar{\partial }}^A_{\sigma }{\bar{\psi }}+i{\bar{\lambda }}_{\sigma {\beta }}V^{{\beta }})]. \end{aligned}$$

By the harmonic coordinates condition (2.19), (2.27) and the Coulomb gauge condition (2.16) the first term in the right hand side is written as

$$\begin{aligned} \nabla ^{\gamma }\partial _t A_{\gamma }&= g^{{\beta }\gamma }\nabla _{{\beta }}\partial _t A_{\gamma }=g^{{\beta }\gamma }(\partial _{{\beta }}\partial _tA_{\gamma }-\Gamma ^{\sigma }_{{\beta }\gamma }\partial _tA_{\sigma })=g^{{\beta }\gamma }\partial _{{\beta }}\partial _tA_{\gamma }\\&= \partial _t(g^{{\beta }\gamma }\partial _{{\beta }}A_{\gamma })-\partial _t g^{{\beta }\gamma }\cdot \partial _{{\beta }}A_{\gamma }\\&= \partial _t\nabla ^{\gamma }A_{\gamma }+(2\mathop {\mathrm{Im}}\nolimits (\psi {\bar{\lambda }}^{{\beta }\gamma })+\nabla ^{{\beta }}V^{\gamma }+\nabla ^{\gamma }V^{{\beta }})\partial _{{\beta }}A_{\gamma }\\&= (2\mathop {\mathrm{Im}}\nolimits (\psi {\bar{\lambda }}^{{\beta }\gamma })+\nabla ^{{\beta }}V^{\gamma }+\nabla ^{\gamma }V^{{\beta }})\partial _{{\beta }}A_{\gamma }. \end{aligned}$$

We then obtain the *B*-equation. $\square$

Next, we use (2.31) to derive the main equation, i.e. the Schrödinger equation for $\psi$. By (2.10), the right-hand side of (2.31) is rewritten as

$$\begin{aligned} \nabla ^A_{\alpha }(\partial ^{A,\sigma } \psi -i\lambda ^{\sigma }_{\gamma }V^{\gamma } ) =\nabla ^A_{\alpha } \partial ^{A,\sigma }\psi -i\nabla ^A_{\gamma } \lambda ^{\sigma }_{\alpha }V^{\gamma }-i\lambda ^{\sigma }_{\gamma }\nabla _{\alpha } V^{\gamma }. \end{aligned}$$

Hence, we have

$$\begin{aligned} (\partial ^{B}_t-V^{\gamma }\nabla ^A_{\gamma })\lambda ^{\sigma }_{\alpha }+\lambda ^{\gamma }_{\alpha }\mathop {\mathrm{Im}}\nolimits (\psi {\bar{\lambda }}^{\sigma }_{\gamma })+(\lambda ^{\gamma }_{\alpha }\nabla _{\gamma } V^{\sigma }-\lambda ^{\sigma }_{\gamma } \nabla _{\alpha } V^{\gamma })=i\nabla ^A_{\alpha }\nabla ^{A,\sigma }\psi , \end{aligned}$$

and then contracting this yields

$$\begin{aligned} i(\partial ^{B}_t-V^{\gamma }\nabla ^A_{\gamma })\psi +\nabla ^A_{\alpha }\nabla ^{A,\alpha }\psi =-i\lambda ^{\gamma }_{\sigma }\mathop {\mathrm{Im}}\nolimits (\psi {\bar{\lambda }}^{\sigma }_{\gamma }). \end{aligned}$$

This can be further written as

$$\begin{aligned} i(\partial _t+iB-V^{\gamma }\nabla ^A_{\gamma })\psi +(\nabla _{\alpha }+iA_{\alpha })(\nabla ^{\alpha }+iA^{\alpha })\psi =-i\lambda _{\sigma }^{\gamma }\mathop {\mathrm{Im}}\nolimits (\psi {\bar{\lambda }}^{\sigma }_{\gamma }). \end{aligned}$$

Hence, under the harmonic coordinates condition (2.19) and the Coulomb gauge condition (2.16) we obtain the main Schrödinger equation

2.34 $$\begin{aligned} i\partial _t\psi +g^{\alpha {\beta }}\partial _{\alpha }\partial _{{\beta }}\psi&\!=\! \ iV^{\gamma }\nabla ^A_{\gamma }\psi -2iA_{\alpha }\nabla ^{\alpha }\psi +(B+A_{\alpha }A^{\alpha }\!-\!i\nabla _{\alpha }A^{\alpha })\psi \!-\!i\lambda _{\sigma }^{\gamma }\mathop {\mathrm{Im}}\nolimits (\psi {\bar{\lambda }}^{\sigma }_{\gamma })\nonumber \\&= \ iV^{\gamma }\nabla ^A_{\gamma }\psi -2iA_{\alpha }\nabla ^{\alpha }\psi +(B+A_{\alpha }A^{\alpha })\psi -i\lambda _{\sigma }^{\gamma }\mathop {\mathrm{Im}}\nolimits (\psi {\bar{\lambda }}^{\sigma }_{\gamma }). \end{aligned}$$

In conclusion, under the Coulomb gauge condition $\nabla ^{\alpha }A_{\alpha }=0$ and the harmonic coordinate condition $g^{\alpha {\beta }}\Gamma ^{\gamma }_{\alpha {\beta }}=0$, by (2.34), (2.14), (2.22), (2.30), (2.17) and (2.33), we obtain the Schrödinger equation for the complex mean curvature $\psi$

2.35 $$\begin{aligned} \left\{ \begin{aligned}&i\partial _t\psi +g^{\alpha {\beta }}\partial _{\alpha }\partial _{{\beta }}\psi =i(V-2A)_{\alpha }\nabla ^{\alpha }\psi +(B+A_{\alpha }A^{\alpha }-V_{\alpha }A^{\alpha })\psi -i\lambda _{\sigma }^{\gamma }\mathop {\mathrm{Im}}\nolimits (\psi {\bar{\lambda }}^{\sigma }_{\gamma }), \\&\psi (0) = \psi _0, \end{aligned} \right. \end{aligned}$$

where the metric *g*, curvature tensor $\lambda$, the advection field *V*, connection coefficients *A* and *B* are determined at fixed time in an elliptic fashion via the following equations

2.36 $$\begin{aligned} \left\{ \begin{aligned}&\nabla ^A_\alpha \lambda _{{\beta }\gamma }-\nabla ^A_{\beta }\lambda _{\alpha \gamma }=0,\quad \nabla ^{A,\alpha }\lambda _{\alpha {\beta }}=\nabla ^A_{\beta }\psi ,\\&\begin{aligned} g^{\alpha {\beta }}\partial ^2_{\alpha {\beta }}g_{\gamma \sigma }&= \ [-\partial _{\gamma } g^{\alpha {\beta }}\partial _{{\beta }} g_{\alpha \sigma }-\partial _{\sigma } g^{\alpha {\beta }}\partial _{{\beta }} g_{\alpha \gamma }+\partial _{\gamma } g_{\alpha {\beta }}\partial _{\sigma } g^{\alpha {\beta }}]\\&\quad +2g^{\alpha {\beta }}\Gamma _{\sigma \alpha ,\nu }\Gamma ^{\nu }_{{\beta }\gamma }-2\mathop {\mathrm{Re}}\nolimits (\lambda _{\gamma \sigma }{\bar{\psi }}-\lambda _{\alpha \gamma }{\bar{\lambda }}_{\sigma }^{\alpha }), \end{aligned}\\&\begin{aligned} \nabla ^{\alpha }\nabla _{\alpha }V^{\gamma }&= \ -2\nabla _\alpha \mathop {\mathrm{Im}}\nolimits (\psi {\bar{\lambda }}^{\alpha \gamma })-\mathop {\mathrm{Re}}\nolimits (\lambda ^{\gamma }_{\sigma }{\bar{\psi }}-\lambda _{\alpha \sigma }{\bar{\lambda }}^{\alpha \gamma })V^{\sigma }\\&\quad +2(\mathop {\mathrm{Im}}\nolimits (\psi {\bar{\lambda }}^{\alpha {\beta }})+\nabla ^{\alpha }V^{{\beta }})\Gamma ^{\gamma }_{\alpha {\beta }}, \end{aligned}\\&\nabla ^{\gamma }\nabla _{\gamma } A_{\alpha }=\mathop {\mathrm{Re}}\nolimits (\psi {\bar{\lambda }}_{\alpha }^{\sigma }-\lambda _{\alpha }^{{\beta }}{\bar{\lambda }}_{{\beta }}^{\sigma })A_{\sigma }+\nabla ^{\gamma }\mathop {\mathrm{Im}}\nolimits (\lambda ^{\sigma }_{\gamma }{\bar{\lambda }}_{\alpha \sigma }),\\&\begin{aligned}\nabla ^{\gamma }\nabla _{\gamma }B&= -\nabla ^{\gamma }[\mathop {\mathrm{Re}}\nolimits (\lambda _{\gamma }^{\sigma }{\bar{\partial }}^A_{\sigma }{\bar{\psi }})-\mathop {\mathrm{Im}}\nolimits (\lambda ^\sigma _\gamma {\bar{\lambda }}_{\sigma {\beta }})V^{\beta }]\\&\quad +(2\mathop {\mathrm{Im}}\nolimits (\psi {\bar{\lambda }}^{{\beta }\gamma })+\nabla ^{{\beta }}V^{\gamma }+\nabla ^{\gamma }V^{{\beta }})\partial _{{\beta }}A_{\gamma }.\end{aligned} \end{aligned}\right. \end{aligned}$$

Fixing the remaining degrees of freedom (i.e. the affine group for the choice of the coordinates as well as the time dependence of the *SU*(1) connection) we can assume that the following conditions hold at infinity in an averaged sense:

$$\begin{aligned} \lambda (\infty )=0,\quad g(\infty ) = I_d, \quad V(\infty ) = 0,\quad A(\infty ) = 0, \quad B(\infty ) = 0 \end{aligned}$$

These are needed to insure the unique solvability of the above elliptic equations in a suitable class of functions. For the metric *g* it will be useful to use the representation

$$\begin{aligned} g = I_d + h \end{aligned}$$

so that *h* vanishes at infinity.

Finally, we note that the above system (2.35)-(2.36) is accompanied by a large family of compatibility conditions as follows:

(i) The trace relation (2.4).
(ii) The Gauss equations (2.8) connecting the curvature *R* of *g* and $\lambda$.
(iii) The symmetry property (2.15).
(iv) The Ricci equations (2.13) for the curvature of *A*.
(v) The Coulomb gauge condition (2.16) for *A*.
(vi) The harmonic coordinates condition (2.19) for *g*.
(vii) The time evolution (2.26) for the metric *g* (2.26).
(viii) The time evolution (2.31) for the second fundamental form $\lambda$ .
(ix) The time evolution (2.32) for *A* .

These conditions will all be shown to be satisfied for small solutions to the nonlinear elliptic system (2.35).

Now we can restate here the small data local well-posedness result for the (SMCF) system in Theorem 1.2 in terms of the above system:

#### Theorem 2.7

(Small data local well-posedness in the good gauge). Let $s>\frac{d}{2}$, $d\ge 4$. Then there exists $\epsilon _0>0$ sufficiently small such that, for all initial data $\psi _0$ with

$$\begin{aligned} \Vert \psi _0\Vert _{H^s}\le \epsilon _0, \end{aligned}$$

the modified Schrödinger system (2.35), with $(\lambda ,h,V,A,B)$ determined via the elliptic system (2.36), is locally well-posed in $H^s$ on the time interval $I=[0,1]$. Moreover, the mean curvature satisfies the bounds

2.37 $$\begin{aligned} \Vert \psi \Vert _{l^2 {\mathbf {X}}^s} + \Vert (\lambda ,h,V,A,B)\Vert _{{\varvec{ {\mathcal {E}}}}^s}\lesssim \Vert \psi _0\Vert _{H^s}. \end{aligned}$$

In addition, the auxiliary functions $(\lambda ,h,V,A,B)$ satisfy the constraints (2.4), (2.8), (2.15), (2.13), (2.16) and (2.19), and the time evolutions (2.26), (2.31) and (2.32).

Here the solution $\psi$ satisfies in particular the expected bounds

$$\begin{aligned} \Vert \psi \Vert _{C[0,1;H^s]} \lesssim \Vert \psi _0\Vert _{H^s}. \end{aligned}$$

The spaces $l^2 {\mathbf {X}}^s$ and ${\varvec{ {\mathcal {E}}}}^s$, defined in the next section, contain a more complete description of the full set of variables $\psi ,\lambda ,h,V,A,B$, which includes both Sobolev regularity and local energy bounds.

In the above theorem, by well-posedness we mean a full Hadamard-type well-posedness, including the following properties:

(i) Existence of solutions $\psi \in C[0,1;H^s]$, with the additional regularity properties (2.37).
(ii) Uniqueness in the same class.
(iii) Continuous dependence of solutions with respect to the initial data in the strong $H^s$ topology.
(iv) Weak Lipschitz dependence of solutions with respect to the initial data in the weaker $L^2$ topology.
(v) Energy bounds and propagation of higher regularity.

## Function Spaces and Notations

The goal of this section is to define the function spaces where we aim to solve the (SMCF) system in the good gauge, given by (2.35). Both the spaces and the notation presented in this section are similar to those introduced in [21–23]. All the function spaces described below will be used with respect to harmonic coordinates determined by our gauge choices described in the previous section. We neither attempt nor need to transfer these spaces to other coordinate frames.

For a function *u*(*t*, *x*) or *u*(*x*), let $\hat{u}={{\mathcal {F}}}u$ denote the Fourier transform in the spatial variable *x*. Fix a smooth radial function $\varphi :{{\mathbb {R}}}^d \rightarrow [0,1]$ supported in $[-2,2]$ and equal to 1 in $[-1,1]$, and for any $i\in {{\mathbb {Z}}}$, let

$$\begin{aligned} \varphi _i(x)\,{:}{=}\,\varphi (x/2^i)-\varphi (x/2^{i-1}). \end{aligned}$$

We then have the spatial Littlewood-Paley decomposition,

$$\begin{aligned} \sum _{i=-\infty }^{\infty }P_i (D)=1, \quad \sum _{i=0}^{\infty }S_i (D)=1, \end{aligned}$$

where $P_i$ localizes to frequency $2^i$ for $i\in {{\mathbb {Z}}}$, i.e,

$$\begin{aligned} {{\mathcal {F}}}(P_iu)=\varphi _i(\xi )\hat{u}(\xi ), \end{aligned}$$

and

$$\begin{aligned} S_0(D)=\sum _{i\le 0}P_i(D),\quad S_i(D)=P_i(D),\ \text { for}\ i>0. \end{aligned}$$

For simplicity of notation, we set

$$\begin{aligned} u_j=S_j u,\quad u_{\le j}=\sum _{i=0}^j S_i u,\quad u_{\ge j}=\sum _{i=j}^{\infty } S_i u,\quad \text {for }j\ge 0. \end{aligned}$$

For each $j\in {{\mathbb {N}}}$, let ${{\mathcal {Q}}}_j$ denote a partition of ${{\mathbb {R}}}^d$ into cubes of side length $2^j$, and let $\{\chi _Q\}$ denote an associated partition of unity. For a translation-invariant Sobolev-type space *U*, set $l^p_j U$ to be the Banach space with associated norm

$$\begin{aligned} \Vert u\Vert _{l^p_j U}^p=\sum _{Q\in {{\mathcal {Q}}}_j}\Vert \chi _Q u\Vert _U^p \end{aligned}$$

with the obvious modification for $p=\infty$.

Next we define the $l^2{\mathbf {X}}^s$ and $l^2N^s$ spaces, which will be used for the primary variable $\psi$, respectively for the source term in the Schrödinger equation for $\psi$. Following [21–23], we first define the *X*-norm as

$$\begin{aligned} \Vert u\Vert _{X}=\sup _{l \in {{\mathbb {N}}}} \sup _{Q\in {{\mathcal {Q}}}_l} 2^{-\frac{l}{2}}\Vert u\Vert _{L^2L^2([0,1]\times Q)}. \end{aligned}$$

Here and throughout, $L^pL^q$ represents $L^p_tL^q_x$. To measure the source term, we use an atomic space *N* satisfying $X=N^{*}$. A function *a* is an atom in *N* if there is a $j\ge 0$ and a $Q\in {{\mathcal {Q}}}_j$ such that *a* is supported in $[0,1]\times Q$ and

$$\begin{aligned} \Vert a\Vert _{L^2([0,1]\times Q)}\lesssim 2^{-\frac{j}{2}}. \end{aligned}$$

Then we define *N* as linear combinations of the form

$$\begin{aligned} f=\sum _k c_k a_k,\ \ \sum _k|c_k|<\infty ,\ \ a_k\ \mathrm{atom}, \end{aligned}$$

with norm

$$\begin{aligned} \Vert f\Vert _N=\inf \big \{\sum _k |c_k|: f=\sum _k c_k a_k,\ a_k\ \mathrm{atoms}\big \}. \end{aligned}$$

For solutions which are localized to frequency $2^j$ with $j \ge 0$, we will work in the space

$$\begin{aligned} X_j=2^{-\frac{j}{2}}X\cap L^{\infty }L^2, \end{aligned}$$

with norm

$$\begin{aligned} \Vert u\Vert _{X_j}=2^{\frac{j}{2}}\Vert u\Vert _X+\Vert u\Vert _{L^{\infty }L^2}. \end{aligned}$$

One way to assemble the $X_j$ norms is via the $X^s$ space

$$\begin{aligned} \Vert u\Vert _{X^s}^2=\sum _{j\ge 0} 2^{2js}\Vert S_j u\Vert _{X_j}^2. \end{aligned}$$

But we will also add the $l^p$ spatial summation on the $2^j$ scale to $X_j$, in order to obtain the space $l^p_j X_j$ with norm

$$\begin{aligned} \Vert u\Vert _{l^p_j X_j} =(\sum _{Q\in {{\mathcal {Q}}}_j} \Vert \chi _Q u\Vert _{X_j}^p)^{1/p}. \end{aligned}$$

We then define the space $l^p X^s$ by

$$\begin{aligned} \Vert u\Vert _{l^p X^s}^2=\sum _{j\ge 0}2^{2js}\Vert S_j u\Vert _{l^p_j X_j}^2. \end{aligned}$$

For the solutions of Schrödinger equation in (2.35), we will be working primarily in $l^2 {\mathbf {X}}^s$, which is defined by

$$\begin{aligned} \Vert u\Vert _{l^2{\mathbf {X}}^s}=\Vert u\Vert _{l^2X^s}+\Vert \partial _t u\Vert _{L^2H^{s-2}}. \end{aligned}$$

We note that the second component, introduced here for the first time, serves the purpose of providing better bounds at low frequencies $j \le 0$.

We analogously define

$$\begin{aligned} N_j=2^{\frac{j}{2}}N+L^1L^2, \end{aligned}$$

which has norm

$$\begin{aligned} \Vert f\Vert _{N_j}=\inf _{f=2^{\frac{j}{2}}f_1+f_2} \big (\Vert f_1\Vert _N+\Vert f_2\Vert _{L^1L^2}\big ), \end{aligned}$$

and

$$\begin{aligned} \Vert f\Vert _{l^pN^s}^2=\sum _{j\ge 0}2^{2js}\Vert S_j f\Vert _{l^p_j N_j}^2. \end{aligned}$$

Here we shall be working primarily with $l^2N^s$.

We also note that for any $j\in {{\mathbb {N}}}$, we have

$$\begin{aligned} \sup _{Q\in {{\mathcal {Q}}}_j} 2^{-\frac{j}{2}}\Vert u\Vert _{L^2L^2([0,1]\times Q)}\le \Vert u\Vert _{X}, \end{aligned}$$

hence

$$\begin{aligned} \Vert u\Vert _N\lesssim 2^{j/2}\Vert u\Vert _{l^1_jL^2L^2}. \end{aligned}$$

This bound will come in handy at several places later on.

For the elliptic system (2.36), at a fixed time we define the ${\mathcal {H}}^s$ norm,

$$\begin{aligned} \Vert (\lambda ,h,V,A,B)\Vert _{\mathcal {H}^s}=\Vert \lambda \Vert _{H^{s}}+\Vert |D|h\Vert _{H^{s+1}} +\Vert |D|V\Vert _{H^{s}}+\Vert |D|A\Vert _{H^{s}}+\Vert |D|B\Vert _{H^{s-1}}. \end{aligned}$$

In addition to the fixed time norms, for the study of the Schrödinger equation for $\psi$ we will also need to bound time dependent norms ${{\mathcal {E}}}^s$ and ${\varvec{ {\mathcal {E}}}}^s$ for the elliptic system (2.36), in terms of similar norms for $\psi$. For simplicity of notation, we define

$$\begin{aligned} \Vert u\Vert _{Z^{\sigma ,s}}=\Vert |D|^{\sigma }S_0 u\Vert _{l^2_0L^{\infty }L^2}^2+\sum _{j>0} 2^{2sj} \Vert S_ju\Vert _{l^2_jL^{\infty }L^2}^2. \end{aligned}$$

Then the ${\mathbf {Z}}^{\sigma ,s}$ spaces are defined by

$$\begin{aligned} \Vert u\Vert _{{\mathbf {Z}}^{\sigma ,s}}=\Vert u\Vert _{Z^{\sigma ,s}}+\Vert |D|^{\sigma }\partial _t u\Vert _{L^2H^{s-\sigma -2}}. \end{aligned}$$

For the $\lambda$, *V*, *A* and *B*-equations in (2.36), we will be working primarily in ${\mathbf {Z}}^{0,s}$, ${\mathbf {Z}}^{1,s+1}$, ${\mathbf {Z}}^{1,s+1}$ and ${\mathbf {Z}}^{1,s}$, respectively.

On the other hand, for the metric component $h=g-I_d$ we need to introduce some additional structure which is associated to spatial scales larger than the frequency. Precisely, to measure the portion of *h* which is localized to frequency $2^j$, $j\in {{\mathbb {Z}}}$, we decompose $P_j h$ as an atomic summation of components $h_{j,l}$ associated to spatial scales $2^l$ with $l\ge |j|$, where $h_{j,l}$ still localizes to frequency $2^j$, i.e.,

$$\begin{aligned} P_jh=\sum _{l\ge |j|}h_{j,l}. \end{aligned}$$

Then we define the $Y_j$-norm by

$$\begin{aligned} \Vert P_j h \Vert _{Y_j} =\inf _{P_jh=\sum _{l\ge |j|}h_{j,l}} \sum _{l\ge |j|} 2^{l-|j|} \Vert h_{j,l}\Vert _{l^1_lL^{\infty }L^2}. \end{aligned}$$

Assembling together the dyadic pieces in an $l^2$ Besov fashion, we obtain the $Y^{\sigma ,s}$ space with norm given by

$$\begin{aligned} \Vert h\Vert _{Y^{\sigma ,s}}^2=\sum _{j\le {{\mathbb {Z}}}} 2^{2(\sigma j^-+sj^+)}\Vert P_jh\Vert _{Y_j}^2. \end{aligned}$$

Then for *h*-equation in (2.35), we will be working primarily in ${\mathbf {Y}}^{s+2}$, whose norm is defined by

$$\begin{aligned} \Vert h\Vert _{{\mathbf {Y}}^{s+2}}=\Vert h\Vert _{Y^{s+2}}+\Vert \nabla \partial _t h\Vert _{L^2H^{s-1}}&= \Vert h\Vert _{Y^{\frac{d}{2}-1-\delta ,s+2}}+\Vert h\Vert _{{\mathbf {Z}}^{1,s+2}}, \end{aligned}$$

where the space $Y^s=Y^{\frac{d}{2}-1-\delta ,s}\cap Z^{1,s}$. Collecting all the components defined above, for the elliptic system (2.36), we define the ${{\mathcal {E}}}^s$ norm as

$$\begin{aligned} \Vert (\lambda ,h,V,A,B)\Vert _{{{\mathcal {E}}}^s}=\Vert \lambda \Vert _{Z^{0,s}}+\Vert h\Vert _{Y^{s+2}} +\Vert V\Vert _{Z^{1,s+1}}+\Vert A\Vert _{Z^{1,s+1}}+\Vert B\Vert _{Z^{1,s}}, \end{aligned}$$

and the ${\varvec{ {\mathcal {E}}}}^s$ norm as

$$\begin{aligned} \Vert (\lambda ,h,V,A,B)\Vert _{{\varvec{ {\mathcal {E}}}}^s}=\Vert (\lambda ,h,V,A,B)\Vert _{{{\mathcal {E}}}^s}+\Vert \partial _t(\lambda ,h,V,A,B)\Vert _{L^2{{\mathcal {H}}}^{s-2}}. \end{aligned}$$

Since we often use Littlewood-Paley decompositions, the next lemma is a convenient tool to see that our function spaces are invariant under the action of some standard classes of multipliers:

### Lemma 3.1

For any Schwartz function $f\in {{\mathcal {S}}}$, multiplier *m*(*D*) with $\Vert {{\mathcal {F}}}^{-1}(m(\xi ))\Vert _{L^1}<\infty$, and translation-invariant Sobolev-type space *U*, we have

$$\begin{aligned} \Vert m(D)f\Vert _{U}\lesssim \Vert {{\mathcal {F}}}^{-1}(m(\xi ))\Vert _{L^1}\Vert f\Vert _U. \end{aligned}$$

We will also need the following Bernstein-type inequality:

### Lemma 3.2

(Bernstein-type inequality). For any $j,k\in {{\mathbb {Z}}}$ with $j+k\ge 0$, $1\le r<\infty$ and $1\le q\le p\le \infty$, we have

3.1 $$\begin{aligned}&\Vert P_k f\Vert _{l^r_jL^p}\lesssim 2^{kd(\frac{1}{q}-\frac{1}{p})}\Vert P_k f\Vert _{l^r_jL^q}, \end{aligned}$$

3.2 $$\begin{aligned}&\Vert \langle x\rangle ^{\alpha -d}*f_{\le 0}\Vert _{l_0^pL^{\infty }L^2}\lesssim \Vert f_{\le 0}\Vert _{l^1_0L^{\infty }L^2},\ \ \mathrm{for }\ p>\frac{d}{d-\alpha }. \end{aligned}$$

### Proof

We begin with the Bernstein-type inequality (3.1). Using the properies of the Fourier transform, $P_kf$ is rewritten as

$$\begin{aligned} P_k f=\int _{{{\mathbb {R}}}^d} ({{\mathcal {F}}}^{-1}{\varphi }_k) (x-y)P_kf(y)dy=2^{kd}\int _{{{\mathbb {R}}}^d} K(2^k(x-y))S_k f(y)dy, \end{aligned}$$

where $$K(x)={{\mathcal {F}}}^{-1}\varphi (x)$$. Then

$$\begin{aligned} \Vert P_kf\Vert _{l^r_jL^p}&= \ 2^{kd}(\sum _{Q\in {{\mathcal {Q}}}_j}\Vert \chi _Q(x)\int _{{{\mathbb {R}}}^d} K(2^k(x-y))P_k f(y)dy\Vert _{L^p}^r)^{1/r}\\ \le&\ 2^{kd}(\sum _{Q\in {{\mathcal {Q}}}_j}\Vert \chi _Q(x)\int _{{{\mathbb {R}}}^d} K(2^k(x-y)) {\mathbf {1}}_{<M}(2^k(x-y)) P_k f(y)dy\Vert _{L^p}^r)^{1/r}\\&+2^{kd} \Vert K(2^kx){\mathbf {1}}_{>M}(2^kx)*P_k f\Vert _{l^r_j L^p} \\&\,{:}{=}\, \ I+II, \end{aligned}$$

where $d(Q,\tilde{Q})=\inf \{|x-y|:x\in Q,y\in \tilde{Q}\}$ and *M* is a large constant. Since $j+k\ge 0$, for any fixed $Q\in {{\mathcal {Q}}}_j$ there are only finite many $\tilde{Q}\in {{\mathcal {Q}}}_j$ such that $d(Q,\tilde{Q})\le 2^{-k} M$. Then from Young’s inequality and $1+1/p=1/q+1/\tilde{q}$ we can bound *I* by

$$\begin{aligned} I\lesssim 2^{kd}(\sum _{Q\in {{\mathcal {Q}}}_j} \sum _{d(Q,\tilde{Q})\le 2^{-k}M,\tilde{Q}\in {{\mathcal {Q}}}_j}\Vert K(2^kx)\Vert _{L^{\tilde{q}}}^r\Vert \chi _{\tilde{Q}}P_k f\Vert _{L^q}^r)^{1/r} \lesssim 2^{kd(\frac{1}{q}-\frac{1}{p})}\Vert P_kf\Vert _{l^r_jL^q}. \end{aligned}$$

On the other hand, since $|K(x)|\lesssim \langle x\rangle ^{-N}$ for any large *N*, for *II* we have

$$\begin{aligned} II&\lesssim \ 2^{k(d-N)} \Vert |2^kx|^{-N}{\mathbf {1}}_{>M}(2^kx)\Vert _{L^1}\Vert S_k f\Vert _{l^r_j L^p}\\&\lesssim \ M^{-N+d} \Vert P_k f\Vert _{l^r_jL^q}, \end{aligned}$$

which can be absorbed by the term on the left. These imply the bound (3.1).

Next, we prove the estimate (3.2). The left hand side of (3.2) is decomposed as

$$\begin{aligned} \Vert \langle x\rangle ^{\alpha -d}*f_{\le 0}\Vert _{l_0^pL^{\infty }L^2}^p&\lesssim \ \sum _{Q\in {{\mathcal {Q}}}_0}\Vert \chi _Q(x)\int \langle y\rangle ^{\alpha -d}f_{\le 0}(x-y)dy\Vert _{L^{\infty }L^{\infty }}^p\\&\lesssim \sum _{Q\in {{\mathcal {Q}}}_0}\Vert \chi _Q(x)\int _{|y|\le 1} \langle y\rangle ^{\alpha -d}f_{\le 0}(x-y)dy\Vert _{L^{\infty }L^{\infty }}^p\\&\quad +\sum _{Q\in {{\mathcal {Q}}}_0}\Vert \chi _Q(x)\int _{|y|>1} \langle y\rangle ^{\alpha -d}\sum _{\tilde{Q}\in {{\mathcal {Q}}}_0}\chi _{\tilde{Q}}f_{\le 0}(x-y)dy\Vert _{L^{\infty }L^{\infty }}^p\\&= I_1^p +I_2^p. \end{aligned}$$

Then by (3.1) we bound $I_1$ by

$$\begin{aligned} I_1\lesssim \Vert f_{\le 0}\Vert _{l^p_0L^{\infty }L^{\infty }}\lesssim \Vert f_{\le 0}\Vert _{l^p_0L^{\infty }L^2}. \end{aligned}$$

On the other hand, by Hölder’s inequality and (3.1), we bound $I_2$ by

$$\begin{aligned} I_2&\lesssim \sum _{\tilde{Q}\in {{\mathcal {Q}}}_0}(\sum _{Q\in {{\mathcal {Q}}}_0}\Vert \chi _Q(x)\int _{|y|>1} \langle y\rangle ^{\alpha -d}\chi _{\tilde{Q}}f_{\le 0}(x-y)dy\Vert _{L^{\infty }L^{\infty }}^p)^{1/p}\\&\lesssim \sum _{\tilde{Q}\in {{\mathcal {Q}}}_0}(\sum _{Q\in {{\mathcal {Q}}}_0}\int _{|y|>1}\chi _Q\langle y\rangle ^{(\alpha -d)p}dy \Vert \chi _{\tilde{Q}}f_{\le 0}\Vert _{L^{\infty }L^q}^p)^{1/p}\\&\lesssim \Vert f_{\le 0}\Vert _{l^1_0L^{\infty }L^q}(\int \langle y\rangle ^{(\alpha -d)p}dy)^{1/p}\\&\lesssim \Vert f_{\le 0}\Vert _{l^1_0L^{\infty }L^2}, \end{aligned}$$

which gives the bound (3.2), and thus completes the proof of the lemma. $\square$

Finally, we define the frequency envelopes as in [21–23] which will be used in multilinear estimates. Consider a Sobolev-type space *U* for which we have

$$\begin{aligned} \Vert u\Vert _U^2=\sum _{k=0}^{\infty } \Vert S_k u\Vert _U^2. \end{aligned}$$

A frequency envelope for a function $u\in U$ is a positive $l^2$-sequence, $\{ a_j\}$, with

$$\begin{aligned} \Vert S_j u\Vert _U\le a_j. \end{aligned}$$

We shall only permit slowly varying frequency envelopes. Thus, we require $a_0\approx \Vert u\Vert _U$ and

$$\begin{aligned} a_j\le 2^{\delta |j-k|} a_k,\quad j,k\ge 0,\ 0<\delta \ll s-d/2. \end{aligned}$$

The constant $\delta$ only depends on *s* and the dimension *d*. Such frequency envelopes always exist. For example, one may choose

3.3 $$\begin{aligned} a_j=2^{-\delta j}\Vert u\Vert _U+\max _k 2^{-\delta |j-k|} \Vert S_k u\Vert _U. \end{aligned}$$

## Elliptic Estimates

Here we consider the solvability of the elliptic system (2.36), together with the constraints (2.4), (2.8), (2.15), (2.13), (2.19) and (2.16). We will do this in two steps. First we prove that this system is solvable in Sobolev spaces at fixed time. Then we prove space-time bounds in local energy spaces; the latter will be needed in the study of the Schrödinger evolution (2.35).

For simplicity of notations, we define the set of elliptic variables by

$$\begin{aligned} {{\mathcal {S}}}=(\lambda ,h,V,A,B), \end{aligned}$$

Later when we compare two solutions for (2.36), we will denote the differences of two solutions or the linearized variable by

$$\begin{aligned} \delta {{\mathcal {S}}}=(\delta \lambda ,\delta h,\delta V,\delta A,\delta B). \end{aligned}$$

Our fixed time result is as follows:

### Theorem 4.1

a) Assume that $\psi$ is small in $H^s$ for $s > d/2$ and $d \ge 4$. Then the elliptic system (2.36) admits a unique small solution ${{\mathcal {S}}}= (\lambda ,h,V,A,B)$ in ${\mathcal {H}}^s$, with

4.1 $$\begin{aligned} \Vert {{\mathcal {S}}}\Vert _{{\mathcal {H}}^s} \lesssim \Vert \psi \Vert _{H^s}. \end{aligned}$$

In addition this solution has a smooth dependence on $\psi$ in $H^s$ and satisfies the constraints (2.4), (2.8), (2.15), (2.13), (2.19) and (2.16).

b) Let $\psi$ and $(\lambda , h,V,A,B)= {{\mathcal {S}}}(\psi )$ be as above. Then for the linearization of the solution map above we also have the bound:

4.2 $$\begin{aligned} \Vert D{{\mathcal {S}}}(\delta \psi )\Vert _{{\mathcal {H}}^{\sigma }} \lesssim \Vert \delta \psi \Vert _{H^{\sigma }}, \qquad \sigma \in (d/2-3,s]. \end{aligned}$$

Moreover, assume that $\tilde{p}_k$ and $s_k$ are admissible frequency envelopes for $\psi \in H^{\sigma }$, ${{\mathcal {S}}}\in {{\mathcal {H}}}^s$ respectively. Then we have

4.3 $$\begin{aligned} \Vert S_k{{\mathcal {S}}}\Vert _{{{\mathcal {H}}}^{\sigma }}\lesssim \tilde{p}_k+s_k\Vert \delta {{\mathcal {S}}}\Vert _{{{\mathcal {H}}}^{\sigma }}. \end{aligned}$$

c) We also have a similar bound for the Hessian of the solution map,

4.4 $$\begin{aligned} \Vert D^2{{\mathcal {S}}}(\delta _1 \psi ,\delta _2 \psi )\Vert _{{\mathcal {H}}^{\sigma }} \lesssim \Vert \delta _1 \psi \Vert _{H^{\sigma _1}} \Vert \delta _2 \psi \Vert _{H^{\sigma _2}}, \end{aligned}$$

with $$\sigma ,\sigma _1,\sigma _2\in (d/2-3,s], \sigma _1+\sigma _2 = \sigma +s$$.

### Remark 4.1.1

Here we solve the elliptic system (2.36) in the function space ${{\mathcal {H}}}^s$ for $s>d/2$, which is more suitable for the nonlinear estimates of $\psi$-equation. Nevertheless, this system can be solved in a similar fashion for the full range of indices *s* above scaling, namely $s>d/2-1$. However, in the additional range $d/2-1 < s \le d/2$ one needs to replace the above solution space $\mathcal H^s$ with a slightly larger one,

$$\begin{aligned} \Vert {{\mathcal {S}}}\Vert _{\tilde{\mathcal {H}}^s}=\Vert \lambda \Vert _{H^{s}}+\Vert |D|h\Vert _{H^{\sigma +1}} +\Vert |D|V\Vert _{H^{\sigma }}+\Vert |D|A\Vert _{H^{\sigma }}+\Vert |D|B\Vert _{H^{\sigma -1}}, \end{aligned}$$

where $\sigma = 2s-d/2$. Then the elliptic system (2.36) admits a unique small solution ${{\mathcal {S}}}$ in $\tilde{{\mathcal {H}}}^s$ with $\Vert {{\mathcal {S}}}\Vert _{\tilde{{\mathcal {H}}}^s}\lesssim \Vert \psi \Vert _{H^s}.$

### Proof of Theorem 4.1

a) The proof is based on a perturbative argument. We rewrite the system (2.36) in the form

4.5 $$\begin{aligned} \left\{ \begin{aligned}&\partial _\alpha \lambda _{\alpha {\beta }}=\partial _{\beta }\psi +H_{1\lambda },\\&\partial _\alpha \lambda _{{\beta }\gamma }-\partial _{\beta }\lambda _{\alpha \gamma }=H_{2\lambda },\\&\Delta g_{\gamma \sigma }=H_g,\\&\Delta V^{\gamma }=H_V,\\&\Delta A_{\alpha }=H_A,\\&\Delta B=H_B, \end{aligned}\right. \end{aligned}$$

where $\Delta =\sum _{\alpha =1}^d\partial ^2_\alpha$ and the nonlinear source terms are given by

$$\begin{aligned}&H_{1\lambda }= iA_{\beta }\psi -h^{\alpha \mu }\partial _\mu \lambda _{\alpha {\beta }}+\Gamma _{\alpha {\beta },\sigma }\lambda ^{\alpha \sigma },\\&H_{2\lambda }= -iA_\alpha \lambda _{{\beta }\gamma }+iA_{\beta }\lambda _{\alpha \gamma }+\Gamma _{\alpha \gamma ,\sigma }\lambda _{\beta }^\sigma -\Gamma _{{\beta }\gamma ,\sigma }\lambda _\alpha ^\sigma ,\\&\begin{aligned} H_g&= -h^{\alpha {\beta }}\partial ^2_{\alpha {\beta }}g_{\gamma \sigma }-\partial _{\gamma } g^{\alpha {\beta }}\partial _{{\beta }} g_{\alpha \sigma }-\partial _{\sigma } g^{\alpha {\beta }}\partial _{{\beta }} g_{\alpha \gamma }+\partial _{\gamma } g_{\alpha {\beta }}\partial _{\sigma } g^{\alpha {\beta }}\\&\quad +2g^{\alpha {\beta }}\Gamma _{\sigma \alpha ,\nu }\Gamma ^{\nu }_{{\beta }\gamma }-2\mathop {\mathrm{Re}}\nolimits (\lambda _{\gamma \sigma }{\bar{\psi }}-\lambda _{\alpha \gamma }{\bar{\lambda }}_{\sigma }^{\alpha }), \end{aligned}\\&\begin{aligned} H_V&= -\nabla ^{\alpha }\nabla _{\alpha }V^{\gamma }+\Delta V^{\gamma }-2\nabla _\alpha \mathop {\mathrm{Im}}\nolimits (\psi {\bar{\lambda }}^{\alpha \gamma }) -\mathop {\mathrm{Re}}\nolimits (\lambda ^{\gamma }_{\sigma }{\bar{\psi }}-\lambda _{\alpha \sigma }{\bar{\lambda }}^{\alpha \gamma })V^{\sigma }\\&\quad +2(\mathop {\mathrm{Im}}\nolimits (\psi {\bar{\lambda }}^{\alpha {\beta }})+\nabla ^{\alpha }V^{{\beta }})\Gamma ^{\gamma }_{\alpha {\beta }}, \end{aligned}\\&H_A=-\nabla ^{\gamma }\nabla _{\gamma } A_{\alpha }+\Delta A_{\alpha }+\mathop {\mathrm{Re}}\nolimits (\psi {\bar{\lambda }}_{\alpha }^{\sigma }-\lambda _{\alpha }^{{\beta }}{\bar{\lambda }}_{{\beta }}^{\sigma })A_{\sigma }+\nabla ^{\gamma }\mathop {\mathrm{Im}}\nolimits (\lambda ^{\sigma }_{\gamma }{\bar{\lambda }}_{\alpha \sigma }),\\&\begin{aligned} H_B&= -\nabla ^{\gamma }\nabla _{\gamma }B+\Delta B-\nabla ^{\gamma }\mathop {\mathrm{Re}}\nolimits [\lambda _{\gamma }^{\sigma }({\bar{\partial }}^A_{\sigma }{\bar{\psi }}+i{\bar{\lambda }}_{\sigma {\beta }}V^{{\beta }})]\\&\quad +(2\mathop {\mathrm{Im}}\nolimits (\psi {\bar{\lambda }}^{{\beta }\gamma })+\nabla ^{{\beta }}V^{\gamma }+\nabla ^{\gamma }V^{{\beta }})\partial _{{\beta }}A_{\gamma }. \end{aligned} \end{aligned}$$

In order to prove the existence of solutions to (4.5) at a fixed time for small $\psi \in H^s$, we construct solutions to (4.5) iteratively. We define the sets of elliptic variables

$$\begin{aligned} {{\mathcal {S}}}^{(n)}=(\lambda ^{(n)},h^{(n)},V^{(n)},A^{(n)},B^{(n)}), \end{aligned}$$

at each step, based on the scheme

4.6 $$\begin{aligned} \left\{ \begin{aligned}&\partial _\alpha \lambda ^{(n+1)}_{\alpha {\beta }}=\partial _{\beta }\psi +H^{(n)}_{1\lambda },\\&\partial _\alpha \lambda ^{(n+1)}_{{\beta }\gamma }-\partial _{\beta }\lambda ^{(n+1)}_{\alpha \gamma }=H^{(n)}_{2\lambda },\\&\Delta g^{(n+1)}_{\gamma \sigma }=H^{(n)}_g,\\&\Delta V^{(n+1)\gamma }=H^{(n)}_V,\\&\Delta A^{(n+1)}_{\alpha }=H^{(n)}_A,\\&\Delta B^{(n+1)}=H^{(n)}_B, \end{aligned}\right. \end{aligned}$$

with the trivial initialization

$$\begin{aligned} {{\mathcal {S}}}^{(0)}=(0,0,0,0,0), \quad g^{(0)}=h^{(0)}+I_d, \end{aligned}$$

where $H^{(n)}_{1\lambda }$, $H^{(n)}_{2\lambda }$, $H^{(n)}_{g}$, $H^{(n)}_{V}$, $H^{(n)}_{A}$ and $H^{(n)}_{B}$ are defined as $H_{1\lambda }$, $H_{2\lambda }$, $H_{g}$, $H_{V}$, $H_{A}$ and $H_{B}$ with

$$\begin{aligned} {{\mathcal {S}}}={{\mathcal {S}}}^{(n)}. \end{aligned}$$

We will inductively show that

$$\begin{aligned} \Vert {{\mathcal {S}}}^{(n)}\Vert _{{{\mathcal {H}}}^s}\le C\Vert \psi \Vert _{H^s}, \end{aligned}$$

with a large universal constant *C*. This trivially holds for our initialization. Then using a standard Littlewood-Paley decomposition, Bernstein’s inequality and the smallness of our data $$\psi \in H^s$$ in order to estimate the source terms $$H^{(n)}_{1\lambda }$$, $$H^{(n)}_{2\lambda }$$, $$H^{(n)}_{g}$$, $$H^{(n)}_{V}$$, $$H^{(n)}_{A}$$ and $$ H^{(n)}_{B}$$, we obtain

$$\begin{aligned} \Vert {{\mathcal {S}}}^{(n+1)}\Vert _{{{\mathcal {H}}}^s} \lesssim \Vert \psi \Vert _{H^s}+\Vert {{\mathcal {S}}}^{(n)}\Vert _{{{\mathcal {H}}}^s}^2(1+\Vert {{\mathcal {S}}}^{(n)}\Vert _{{{\mathcal {H}}}^s})^N \lesssim \Vert \psi \Vert _{H^s}. \end{aligned}$$

From the iterative scheme (4.6) and $\psi \in H^s$ small, we can repeat the same analysis for successive differences in order to obtain a small Lipschitz constant,

$$\begin{aligned} \Vert {{\mathcal {S}}}^{(n+1)}-{{\mathcal {S}}}^{(n)}\Vert _{{{\mathcal {H}}}^s}\ll \Vert {{\mathcal {S}}}^{(n)}-{{\mathcal {S}}}^{(n-1)}\Vert _{{{\mathcal {H}}}^s}. \end{aligned}$$

Hence the elliptic system (2.36) admits a small solution

$$\begin{aligned} {{\mathcal {S}}}=\lim _{n\rightarrow \infty } {{\mathcal {S}}}^{(n)}\in {{\mathcal {H}}}^s. \end{aligned}$$

The uniqueness and the Lipschitz dependence of the solution on $\psi$ are easily obtained by similar elliptic estimates.

Next, we prove the solution satisfies the constraints (2.4), (2.15), (2.13), (2.16), (2.19) and (2.8). To get started, let us summarize the compatibility conditions we need to verify:

$$\begin{aligned} \psi = g^{\alpha \beta } \lambda _{\alpha \beta };\qquad \lambda _{\alpha \beta } = \lambda _{{\beta }\alpha }; \qquad \nabla _\alpha A_\beta - \nabla _\beta A_\alpha = \mathop {\mathrm{Im}}\nolimits ( \lambda _\alpha ^{\ \gamma } {{\bar{\lambda }}}_{\gamma \beta });\qquad \nabla ^\alpha A_\alpha = 0;\\ g^{\alpha \beta } \Gamma _{\alpha \beta ,\delta } = 0;\qquad {{\,\mathrm{Ric}\,}}_{\gamma {\beta }}=\mathop {\mathrm{Re}}\nolimits (\lambda _{\gamma {\beta }}{\bar{\psi }}-\lambda _{\gamma \alpha }{\bar{\lambda }}_{\ {\beta }}^\alpha );\qquad R_{\sigma \gamma \alpha {\beta }}= \mathop {\mathrm{Re}}\nolimits (\lambda _{\gamma {\beta }}{\bar{\lambda }}_{\sigma \alpha }-\lambda _{\gamma \alpha }{\bar{\lambda }}_{\sigma {\beta }}). \end{aligned}$$

We need to show that these constraints are satisfied for solutions to the elliptic system (2.36). We can disregard the *B* and *V* equations, which are unneeded here.

To shorten the notations, we define

$$\begin{aligned} \begin{aligned}&C^1=\psi - g^{\alpha \beta } \lambda _{\alpha \beta },\qquad C^2_{\alpha {\beta }}=\lambda _{\alpha \beta } -\lambda _{{\beta }\alpha },\\&C^3_{\alpha {\beta }}=\nabla _\alpha A_\beta - \nabla _\beta A_\alpha - \mathop {\mathrm{Im}}\nolimits ( \lambda _{\alpha \gamma } {{\bar{\lambda }}}^\gamma _{\ \beta }),\quad C^4=\nabla ^\alpha A_\alpha ,\quad C^5_\delta =g^{\alpha \beta } \Gamma _{\alpha \beta ,\delta },\\&C^6_{\gamma {\beta }}={{\,\mathrm{Ric}\,}}_{\gamma {\beta }}-\mathop {\mathrm{Re}}\nolimits (\lambda _{\gamma {\beta }}{\bar{\psi }}-\lambda _{\gamma \alpha }{{\bar{\lambda }}^\alpha }_{\ {\beta }}), \quad C^7_{\sigma \gamma \alpha {\beta }}=R_{\sigma \gamma \alpha {\beta }}- \mathop {\mathrm{Re}}\nolimits (\lambda _{\gamma {\beta }}{\bar{\lambda }}_{\sigma \alpha }-\lambda _{\gamma \alpha }{\bar{\lambda }}_{\sigma {\beta }}). \end{aligned} \end{aligned}$$

Here $C^2$ and $C^3$ are antisymmetric, $C^6$ is symmetric and $C^7$ inherits all the linear symmetries of the curvature tensor.

Our goal is to show that all these functions vanish. We will prove this by showing that they solve a coupled linear homogeneous elliptic system of the form

$$\begin{aligned} \begin{aligned} \nabla ^A_{\beta }C^1&= \ \nabla ^{A,\alpha }C^2_{\alpha {\beta }}, \\ \Delta ^A_g C^2&= \ (\lambda +\psi )(C^3+C^6+C^7)+(\lambda ^2+\lambda \psi )C^2, \\ \Delta _g C^3&= \ R C^3+ \nabla ( C^6 A) + \nabla ( \lambda \nabla C^2+\nabla \lambda C^2) ,\\ \Delta _g C^4&= \ \nabla ( C^6 A) + R C^3 +\nabla ^2(\lambda C^2), \\ \Delta _g C^5&= \ R C^5 + \nabla ( C^1 \psi )+\lambda \nabla C^2+\nabla \lambda C^2 ,\\ C^6_{\gamma \sigma }&= \ \frac{1}{2}(\nabla _\gamma C^5_\sigma +\nabla _\sigma C^5_\gamma ), \\ \nabla _\delta C^7_{\sigma \gamma \alpha {\beta }}&\ + \nabla _{\sigma } C^7_{\gamma \delta \alpha {\beta }} + \nabla _{\gamma } C^7_{\delta \sigma \alpha {\beta }} =0, \\ \nabla ^\sigma C^7_{\sigma \gamma \alpha {\beta }}&= \ \nabla _{\alpha } C^6_{\gamma {\beta }} - \nabla _{{\beta }} C^6_{\gamma \alpha } + \nabla (\lambda C^1+\lambda C^2). \end{aligned} \end{aligned}$$

Here the covariant Laplace operators $\Delta _g$, respectively $\Delta _g^A$ are symmetric and coercive in $\dot{H}^1$. We consider these equations as a system in the space

$$\begin{aligned} (C^1,C^2,C^3,C^4,C^5,C^6,C^7) \in \dot{H}^1 \times \dot{H}^1 \times \dot{H}^1 \times \dot{H}^1 \times \dot{H}^1 \times L^2 \times L^2 \end{aligned}$$

using $\dot{H}^1$ bounds for the Laplace operator in the second to fifth equations, and interpreting the last two equations as an elliptic div-curl system in $L^2$, with an $\dot{H}^{-1}$ source term. Since the coefficients are all small, the right hand side terms are perturbative and 0 is the unique solution for this system. The details are left for the reader, as they only involve Sobolev embeddings and Hölder’s inequality.

To complete the argument, we now successively derive the equations in the above system. In the computations below, it is convenient to introduce several auxiliary notations. The curvature of the connection *A* acting on complex valued functions is denoted by

$$\begin{aligned} {\mathbf {F}}_{\alpha \beta } = \partial _\alpha A_\beta - \partial _\beta A_\alpha \end{aligned}$$

so that we have

$$\begin{aligned}{}[\nabla ^A_\alpha ,\nabla ^A_\beta ]\psi = i {\mathbf {F}}_{\alpha \beta } \psi . \end{aligned}$$

We also set

$$\begin{aligned} C^7_{\sigma \gamma \alpha {\beta }}=R_{\sigma \gamma \alpha {\beta }}-{\tilde{R}}_{\sigma \gamma \alpha {\beta }},\qquad {\tilde{R}}_{\sigma \gamma \alpha {\beta }}\,{:}{=}\, \mathop {\mathrm{Re}}\nolimits (\lambda _{\gamma {\beta }}{\bar{\lambda }}_{\sigma \alpha }-\lambda _{\gamma \alpha }{\bar{\lambda }}_{\sigma {\beta }}), \end{aligned}$$

respectively

$$\begin{aligned} C^6_{\gamma {\beta }}={{\,\mathrm{Ric}\,}}_{\gamma {\beta }}-{{\,\mathrm{{\widetilde{Ric}}}\,}}_{\gamma {\beta }},\qquad {{\,\mathrm{{\widetilde{Ric}}}\,}}_{\gamma {\beta }}\,{:}{=}\,\mathop {\mathrm{Re}}\nolimits (\lambda _{\gamma {\beta }}{\bar{\psi }}-\lambda _{\gamma \alpha }{\bar{\lambda }}^\alpha _{\ {\beta }}),\quad {\tilde{R}}\,{:}{=}\,g^{\gamma {\beta }}{{\,\mathrm{{\widetilde{Ric}}}\,}}_{\gamma {\beta }}, \end{aligned}$$

and

$$\begin{aligned} C^3_{\alpha \beta } = {\mathbf {F}}_{\alpha \beta } - \tilde{{\mathbf {F}}}_{\alpha \beta }, \qquad \tilde{{\mathbf {F}}}_{\alpha \beta } \,{:}{=}\, \mathop {\mathrm{Im}}\nolimits ( \lambda _{\alpha \gamma } \bar{\lambda }^\gamma _{\ \beta }). \end{aligned}$$

*The equation for* $C^1$ This equation has the exact form

$$\begin{aligned} \nabla ^A_{\beta }C^1 = \nabla ^{A,\alpha }C^2_{\alpha {\beta }}. \end{aligned}$$

This is obtained by (2.14) directly. $\square$

*The equation for* $C^2$ The full system for $C^2$ has the form

4.7 $$\begin{aligned} \begin{aligned} \Delta ^A_g C^2_{\alpha {\beta }}&= (\lambda +\psi )(C^3+C^6+C^7)+(\lambda ^2+\lambda \psi )C^2. \end{aligned} \end{aligned}$$

By $\lambda$-equation (2.14) we have

$$\begin{aligned} \nabla ^{A,\gamma }\nabla ^A_\gamma \lambda _{\alpha {\beta }}&= [\nabla ^{A,\gamma },\nabla ^A_\alpha ]\lambda _{\gamma {\beta }}+\nabla ^A_\alpha \nabla ^A_{\beta }\psi \\&= {{\,\mathrm{Ric}\,}}_{\alpha \mu }{\lambda ^\mu }_{\beta }+R_{\sigma \alpha {\beta }\mu }\lambda ^{\sigma \mu }+iC^3_{\gamma \alpha }{\lambda ^\gamma }_{\beta }+i\mathop {\mathrm{Im}}\nolimits ({\lambda _\gamma }^\mu {\bar{\lambda }}_{\mu \alpha }){\lambda ^\gamma }_{\beta }+\nabla ^A_\alpha \nabla ^A_{\beta }\psi . \end{aligned}$$

Then we use $C^6$, $C^7$ and $C^3$ to give

$$\begin{aligned} \Delta _g^A C^2_{\alpha {\beta }}&= C^6_{\alpha \mu }{\lambda ^\mu }_{\beta }-C^6_{{\beta }\mu }{\lambda ^\mu }_\alpha +C^7_{\sigma \alpha {\beta }\mu }\lambda ^{\sigma \mu }-C^7_{\sigma {\beta }\alpha \mu }\lambda ^{\sigma \mu }\\&+iC^3_{\gamma \alpha }{\lambda ^\gamma }_{\beta }-iC^3_{\gamma {\beta }}{\lambda ^\gamma }_\alpha +iC^3_{\alpha {\beta }}\psi +C^2(\lambda ^2+\lambda \psi ). \end{aligned}$$

Hence, the $C^2$-equation (4.7) follows. $\square$

*The equation for* $C^3$ This has the form

4.8 $$\begin{aligned} \begin{aligned} \Delta _g C^3_{\alpha {\beta }}&= \ \nabla _\beta ( C^6_{\alpha \delta } A^\delta ) - \nabla _\alpha (C^6_{\beta \delta } A^\delta ) + R_{\beta \alpha \sigma \delta } C^{3,\sigma \delta } + {{\,\mathrm{Ric}\,}}_{\alpha \delta } C^{3,\delta \beta } - {{\,\mathrm{Ric}\,}}_{\beta \delta } C^{3,\delta \alpha }\\&\ + \nabla ^\gamma \mathop {\mathrm{Im}}\nolimits (\lambda _\gamma ^{\ \sigma }(\overline{\nabla ^A_\alpha C^2_{\sigma {\beta }}}-\overline{\nabla ^A_{\beta }C^2_{\sigma \alpha }})+\nabla ^A_\gamma \lambda ^\sigma _{\ {\beta }}\bar{C^2}_{\alpha \sigma }). \end{aligned} \end{aligned}$$

To prove this, it is convenient to separate the left hand side into two terms,

$$\begin{aligned} \Delta _g C^3_{\alpha {\beta }} = ( [\Delta _g, \nabla _\alpha ] A_\beta - [\Delta _g, \nabla _\beta ] A_\alpha ) + (\nabla _\alpha \Delta _g A_\beta - \nabla _\beta \Delta _g A_\alpha - \Delta _g \tilde{{\mathbf {F}}}_{\alpha \beta }) \,{:}{=}\, I + II . \end{aligned}$$

For the commutator we use the Bianchi identities to compute

$$\begin{aligned} I&= \ [\nabla ^\sigma \nabla _\sigma ,\nabla _{\alpha }] A_\beta - [ \nabla ^\sigma \nabla _\sigma , \nabla _\beta ] A_\alpha \\&= \ \nabla ^\sigma (R_{\sigma \alpha \beta \delta } A^\delta - R_{\sigma \beta \alpha \delta } A^\delta ) + (R_{\sigma \alpha \beta \delta } - R_{\sigma \beta \alpha \delta }) \nabla ^\sigma A^\delta + {R^\sigma }_{\alpha \sigma \delta } \nabla ^\delta A_\beta -{R^\sigma }_{\beta \sigma \delta } \nabla ^\delta A_\alpha \\&= \ \nabla ^\sigma R_{\beta \alpha \sigma \delta } A^\delta + 2 R_{\beta \alpha \sigma \delta } \nabla ^\sigma A^\delta + {{\,\mathrm{Ric}\,}}_{\alpha \delta } \nabla ^{\delta } A_\beta - {{\,\mathrm{Ric}\,}}_{\beta \delta } \nabla ^{\delta } A_\alpha \\&= \ (\nabla _\beta {{\,\mathrm{Ric}\,}}_{\alpha \delta } - \nabla _\alpha R_{\beta \delta } ) A^\delta + R_{\beta \alpha \sigma \delta } {\mathbf {F}}^{\sigma \delta }+ {{\,\mathrm{Ric}\,}}_{\alpha \delta } ({{\mathbf {F}}^\delta }_\beta +\nabla _{\beta } A^\delta ) - {{\,\mathrm{Ric}\,}}_{\beta \delta } ({{\mathbf {F}}^\delta }_\alpha + \nabla _{\alpha } A^\delta ) \\&= \ \nabla _\beta ( {{\,\mathrm{Ric}\,}}_{\alpha \delta } A^\delta ) - \nabla _\alpha ({{\,\mathrm{Ric}\,}}_{\beta \delta } A^\delta ) + R_{\beta \alpha \sigma \delta } {\mathbf {F}}^{\sigma \delta }+ {{\,\mathrm{Ric}\,}}_{\alpha \delta } {{\mathbf {F}}^\delta }_\beta - {{\,\mathrm{Ric}\,}}_{\beta \delta } {{\mathbf {F}}^\delta }_\alpha . \end{aligned}$$

On the other hand for the second term we use the *A* equation in (2.36) to write

$$\begin{aligned} II&= \ \nabla _{\alpha }[{{\,\mathrm{{\widetilde{Ric}}}\,}}_{\beta \sigma }A^{\sigma }]-\nabla _{{\beta }}[{{\,\mathrm{{\widetilde{Ric}}}\,}}_{\alpha \sigma } A^{\sigma }] \\&\ +\quad \nabla _{\alpha }\nabla ^{\gamma }\mathop {\mathrm{Im}}\nolimits (\lambda _{\gamma \sigma }{\bar{\lambda }}^\sigma _{\ {\beta }})-\nabla _{{\beta }}\nabla ^{\gamma }\mathop {\mathrm{Im}}\nolimits (\lambda _{\gamma \sigma }{\bar{\lambda }}^\sigma _{\ \alpha })-\nabla _{\gamma }\nabla ^{\gamma }\mathop {\mathrm{Im}}\nolimits (\lambda _{\alpha \sigma }{\bar{\lambda }}^\sigma _{\ {\beta }})\\&\,{:}{=}\, II_1+II_2. \end{aligned}$$

The first term $II_1$ combines directly with the first two terms in *I*. For the second we commute

$$\begin{aligned} II_2&= \ R_{\alpha \gamma \gamma \delta } \tilde{{\mathbf {F}}}^{\delta }_\beta + R_{\alpha \gamma \beta \delta } \tilde{{\mathbf {F}}}^\delta _{\gamma } - R_{\beta \gamma \gamma \delta } \tilde{{\mathbf {F}}}^{\delta }_\alpha - R_{\beta \gamma \alpha \delta } \tilde{{\mathbf {F}}}^\delta _{\gamma } + \\&\ \quad + \nabla ^{\gamma }(\nabla _{\alpha }\mathop {\mathrm{Im}}\nolimits (\lambda _{\gamma \sigma }{\bar{\lambda }}^\sigma _{\ {\beta }})-\nabla _{{\beta }}\mathop {\mathrm{Im}}\nolimits (\lambda _{\gamma \sigma }{\bar{\lambda }}^\sigma _{\ \alpha })-\nabla _{\gamma }\mathop {\mathrm{Im}}\nolimits (\lambda _{\alpha \sigma }{\bar{\lambda }}^\sigma _{\ {\beta }})) \\&= \ - {{\,\mathrm{Ric}\,}}_{\alpha \delta } {\tilde{{\mathbf {F}}}^\delta }_{\ \beta } + {{\,\mathrm{Ric}\,}}_{\beta \delta } {\tilde{{\mathbf {F}}}^\delta }_{\ \alpha } - R_{\beta \alpha \sigma \delta } \tilde{{\mathbf {F}}}^{\sigma \delta }\\&\quad + \nabla ^\gamma \mathop {\mathrm{Im}}\nolimits (\lambda _\gamma ^{\ \sigma }(\overline{\nabla ^A_\alpha C^2_{\sigma {\beta }}}-\overline{\nabla ^A_{\beta }C^2_{\sigma \alpha }})+\nabla ^A_\gamma \lambda ^\sigma _{\ {\beta }}\bar{C^2}_{\alpha \sigma }). \end{aligned}$$

Summing up the expressions for *I* and *II* we obtain (4.8). $\square$

*The equation for* $C^4$ This has the form

4.9 $$\begin{aligned} \begin{aligned} \Delta _g C^4&= -\nabla ^{\sigma }(C^6_{\mu \sigma }A^{\gamma })-\frac{1}{2}[\nabla ^{\alpha },\nabla ^{\gamma }]C^3_{\gamma \alpha }-\frac{1}{2}\nabla ^\gamma \nabla ^\alpha \mathop {\mathrm{Im}}\nolimits (C^2_{\sigma \gamma }{\bar{\lambda }}_\alpha ^{\ \sigma }+\lambda _{\gamma }^{\ \sigma }\bar{C^2}_{\alpha \sigma }) . \end{aligned} \end{aligned}$$

To prove it we commute $\Delta _g$ with $\nabla ^{\alpha }$

$$\begin{aligned} \begin{aligned} \Delta _g \nabla ^{\alpha } A_{\alpha }&= \ \nabla _{\sigma }[\nabla ^{\sigma },\nabla ^{\alpha }]A_{\alpha }+[\nabla _{\sigma },\nabla ^{\alpha }]\nabla ^{\sigma }A_{\alpha }+\nabla ^{\alpha }\Delta _g A_{\alpha } \\&= \ - \nabla ^\sigma ( {{\,\mathrm{Ric}\,}}_{\mu \sigma } A^\mu ) + \frac{1}{2} [\nabla ^{\sigma },\nabla ^{\alpha }] {\mathbf {F}}_{\sigma \alpha } + \nabla ^\alpha ({{\,\mathrm{{\widetilde{Ric}}}\,}}_{\alpha \sigma } A^\sigma ) + \nabla ^\alpha \nabla ^\gamma \tilde{{\mathbf {F}}}_{\alpha \gamma } \end{aligned} \end{aligned}$$

In the last term we can symmetrize in $\alpha$ and $\gamma$, and the desired equation (4.9) follows. $\square$

*The equation for* $C^5$ Here we compute

4.10 $$\begin{aligned} \begin{aligned} \Delta _g C^5_{\beta }&= -[\nabla ^\alpha ,\nabla _{\beta }]C^5_\alpha -\mathop {\mathrm{Re}}\nolimits (\nabla _{\beta }(C^1 {\bar{\psi }})-2{\bar{\lambda }}^{\alpha \sigma }\nabla ^A_\alpha C^2_{\sigma {\beta }}+\nabla _{\beta }(\lambda ^{\alpha \sigma }C^2_{\sigma \alpha }) ). \end{aligned} \end{aligned}$$

We can rewrite the *g* equation (2.22) as

$$\begin{aligned} {{\,\mathrm{Ric}\,}}_{\alpha \beta } = {{\,\mathrm{{\widetilde{Ric}}}\,}}_{\alpha \beta } + \frac{1}{2}(\nabla _\alpha C^5_\beta + \nabla _\beta C^5_\alpha ) \end{aligned}$$

which by contraction yields

$$\begin{aligned} R = {\tilde{R}}+ \nabla ^\alpha C^5_\alpha . \end{aligned}$$

To get to $\Delta _g C^5$, by the above two equalities we write

$$\begin{aligned} \begin{aligned} \frac{1}{2}\Delta _g C^5_\beta&= \ \nabla ^\alpha ({{\,\mathrm{Ric}\,}}_{\alpha \beta } - {{\,\mathrm{{\widetilde{Ric}}}\,}}_{\alpha \beta }) - \frac{1}{2} [\nabla ^\alpha ,\nabla _\beta ] C^5_\alpha - \frac{1}{2} \nabla _\beta (R-{\tilde{R}}) \\&= \ (\nabla ^\alpha R_{\alpha \beta } - \frac{1}{2} \nabla _\beta R) - \frac{1}{2} [\nabla ^\alpha ,\nabla _\beta ] C^5_\alpha - (\nabla ^\alpha {\tilde{R}}_{\alpha \beta } -\frac{1}{2} \nabla _\beta {\tilde{R}}) . \end{aligned} \end{aligned}$$

The first term drops by twice contracted Bianchi,

$$\begin{aligned} g^{\mu \nu }g^{\gamma \alpha }(\nabla _\gamma R_{\nu {\beta }\mu \alpha }+\nabla _\nu R_{{\beta }\gamma \mu \alpha }+\nabla _{\beta }R_{\gamma \nu \mu \alpha })=0, \end{aligned}$$

and the last one is quadratic in $\lambda$ and yields $C^1$ and $C^2$ terms,

$$\begin{aligned} (\nabla ^\alpha {\tilde{R}}_{\alpha \beta }-\frac{1}{2} \nabla _\beta {\tilde{R}})&= \mathop {\mathrm{Re}}\nolimits (\frac{1}{2}\nabla _{\beta }(C^1 {\bar{\psi }})-{\bar{\lambda }}^{\alpha \sigma }\nabla ^A_\alpha C^2_{\sigma {\beta }}+\frac{1}{2}\nabla _{\beta }(\lambda ^{\alpha \sigma }C^2_{\sigma \alpha }) ). \end{aligned}$$

This completes the derivation of (4.10). $\square$

*The equation for* $C^6$ This has the form

4.11 $$\begin{aligned} C^6_{\gamma \sigma }=\frac{1}{2}(\nabla _\gamma C^5_\sigma +\nabla _\sigma C^5_\gamma ). \end{aligned}$$

Indeed, by the *g*-equation in (2.36) and its proof, we recover the Ricci curvature

$$\begin{aligned} \mathop {\mathrm{Re}}\nolimits (\lambda _{\gamma \sigma }{\bar{\psi }}-\lambda _{\gamma \alpha }{\bar{\lambda }}^\alpha _{\ \sigma })={{\,\mathrm{Ric}\,}}_{\gamma \sigma }-\frac{1}{2}(\partial _\gamma C^5_\sigma +\partial _\sigma C^5_\gamma )+\Gamma ^\nu _{\gamma \sigma }C^5_\nu . \end{aligned}$$

This implies the relation (4.11) immediately. $\square$

*The equation for* $C^7$ By the second Bianchi identities of Riemannian curvature and the following equality

$$\begin{aligned}&\nabla _\delta \mathop {\mathrm{Re}}\nolimits (\lambda _{\gamma {\beta }}{\bar{\lambda }}_{\sigma \alpha }-\lambda _{\gamma \alpha }{\bar{\lambda }}_{\sigma {\beta }})+\nabla _\sigma \mathop {\mathrm{Re}}\nolimits (\lambda _{\delta {\beta }}{\bar{\lambda }}_{\gamma \alpha }-\lambda _{\delta \alpha }{\bar{\lambda }}_{\gamma {\beta }})+\nabla _\gamma \mathop {\mathrm{Re}}\nolimits (\lambda _{\sigma {\beta }}{\bar{\lambda }}_{\delta \alpha }-\lambda _{\sigma \alpha }{\bar{\lambda }}_{\delta {\beta }})=0, \end{aligned}$$

we have the counterpart of the second Bianchi identities

$$\begin{aligned} \nabla _\delta C^7_{\sigma \gamma \alpha {\beta }} + \nabla _{\sigma } C^7_{\gamma \delta \alpha {\beta }} + \nabla _{\gamma } C^7_{\delta \sigma \alpha {\beta }} =0, \end{aligned}$$

which combine with the algebraic symmetries of the same tensor to yield an elliptic system for $C^7$. Precisely, using the above relation we have

$$\begin{aligned} \nabla ^{\sigma }C^7_{\sigma \gamma \alpha {\beta }} = \nabla _{\alpha } C^6_{\gamma {\beta }} - \nabla _{{\beta }} C^6_{\gamma \alpha } + \nabla (\lambda C^1+\lambda C^2), \end{aligned}$$

which combined with the previous one yields the desired elliptic system, with $C^6$ viewed as a source term. $\square$

b) Assume that ${{\tilde{s}}}_k$ and $s_k$ are admissible frequency envelopes for $\delta {{\mathcal {S}}}\in {{\mathcal {H}}}^{\sigma }$ and ${{\mathcal {S}}}\in {{\mathcal {H}}}^{s}$, respectively. In view of the bound (4.1) and of the smallness of $\Vert \psi \Vert _{H^s}$, it suffices to prove the difference or linearized estimate

4.12 $$\begin{aligned} \Vert S_k\delta {{\mathcal {S}}}\Vert _{{\mathcal {H}}^{\sigma }} \lesssim \Vert S_k\delta \psi \Vert _{H^{\sigma }}+({{\tilde{s}}}_k\Vert \psi \Vert _{H^s}+s_k\Vert \delta {{\mathcal {S}}}\Vert _{{{\mathcal {H}}}^{\sigma }} ) (1+\Vert \psi \Vert _{H^s})^N. \end{aligned}$$

If this is true, then the bound (4.2) follows. Thus, by the definition of frequency envelope (3.3), (4.2) and the smallness of $\psi \in H^s$, the bound (4.12) with operator $\delta =Id$ and $\sigma =s$ also implies the bound (4.3).

As an intermediate step in the proof of (4.2), we collect in the next Lemma several bilinear estimates. The proof of this Lemma is standard by Littlewood-Paley decompositions and Bernstein inequality.

### Lemma 4.2

Let $d/2-3<\sigma \le s$, $d\ge 3$, then we have

$$\begin{aligned} \Vert \nabla \delta (\tilde{h}h)\Vert _{H^{\sigma }}\lesssim \Vert \nabla \delta \tilde{h}\Vert _{H^{\sigma }}\Vert \nabla h\Vert _{H^{s}}+\Vert \nabla \tilde{h}\Vert _{H^{s}}\Vert \nabla \delta h\Vert _{H^{\sigma }},\\ \Vert \delta (\lambda h)\Vert _{H^{\sigma }}\lesssim \Vert \delta \lambda \Vert _{H^{\sigma }}\Vert \nabla h\Vert _{H^{s}}+\Vert \lambda \Vert _{H^{s}}\Vert \nabla \delta h\Vert _{H^{\sigma }},\\ \Vert \nabla \delta (Ah)\Vert _{H^{\sigma -1}}\lesssim \Vert \nabla \delta A\Vert _{H^{\sigma -1}}\Vert \nabla h\Vert _{H^{s}}+\Vert \nabla A\Vert _{H^{s}}\Vert \nabla \delta h\Vert _{H^{\sigma }}. \end{aligned}$$

Now we turn our attention to the proof of (4.12). Here we first prove the estimates for $\delta \lambda$. By $\lambda$-equations in (4.5) it suffices to consider the following form

$$\begin{aligned}&\partial _\alpha \delta \lambda _{\alpha {\beta }}=\partial _{\beta }\delta \psi + \delta A\psi +A\delta \psi +\delta h\nabla \lambda +h \nabla \delta \lambda +\nabla \delta h\lambda +\nabla h\delta \lambda ,\\&\partial _\alpha \delta \lambda _{{\beta }\gamma }-\partial _{\beta }\delta \lambda _{\alpha \gamma }= \delta A\lambda +A \delta \lambda +\nabla \delta h\lambda +\nabla h\delta \lambda . \end{aligned}$$

By the relation

4.13 $$\begin{aligned} \widehat{\lambda }(\xi )=|\xi |^{-2}(\widehat{\lambda }\cdot \xi )\xi +|\xi |^{-2}(\widehat{\lambda }\xi ^{\top }-\xi \widehat{\lambda }^{\top })\cdot \xi , \end{aligned}$$

we obtain

$$\begin{aligned} \Vert S_k\delta \lambda \Vert _{H^\sigma }&\lesssim \Vert S_k\delta \psi \Vert _{H^\sigma }+\Vert |D|^{-1}S_k[\delta A(\lambda +\psi )+A(\delta \lambda +\delta \psi )+\delta h\nabla \lambda +h \nabla \delta \lambda \\&\qquad +\nabla \delta h\lambda +\nabla h\delta \lambda ]\Vert _{H^\sigma }\\&\lesssim \Vert S_k\delta \psi \Vert _{H^\sigma }+{{\tilde{s}}}_k\Vert {{\mathcal {S}}}\Vert _{{{\mathcal {H}}}^s}+s_k\Vert \delta {{\mathcal {S}}}\Vert _{{{\mathcal {H}}}^\sigma }. \end{aligned}$$

Next we provide the estimate for $\delta A$; the other estimates can be proved similarly. By *A*-equation in (4.5) and Lemma 4.2, it suffices to consider the following form

$$\begin{aligned} \Delta \delta A&= \delta h\nabla ^2 A+h\nabla ^2\delta A+\nabla \delta h\nabla A+\nabla h\nabla \delta A+\nabla \delta h\nabla h A+(\nabla h)^2\delta A\\&\quad +\delta \lambda \lambda (A+\nabla h)+\lambda ^2 (\delta A+\nabla \delta h)+\nabla \lambda \delta \lambda +\lambda \nabla \delta \lambda . \end{aligned}$$

Using Littlewood–Paley trichotomy and Bernstein inequality, we bound all the nonlinearities except $\nabla \lambda \delta \lambda$ and $\lambda \nabla \delta \lambda$ by

$$\begin{aligned}&\Vert |D|^{-1}S_k (\delta h\nabla ^2 A+h\nabla ^2\delta A+\nabla \delta h\nabla A+\nabla h\nabla \delta A+\nabla \delta h\nabla h A+(\nabla h)^2\delta A) \Vert _{H^\sigma }\\&\quad +\Vert |D|^{-1}S_k (\delta \lambda \lambda (A+\nabla h)+\lambda ^2 (\delta A+\nabla \delta h)) \Vert _{H^\sigma }\\&\lesssim ({{\tilde{s}}}_k\Vert {{\mathcal {S}}}\Vert _{{{\mathcal {H}}}^s}+s_k\Vert \delta {{\mathcal {S}}}\Vert _{{{\mathcal {H}}}^\sigma })(1+\Vert {{\mathcal {S}}}\Vert _{{{\mathcal {H}}}^s}). \end{aligned}$$

For the remainder terms, we can also bound their low-frequency part by

$$\begin{aligned}&\Vert |D|^{-1}S_0(\nabla \lambda \delta \lambda +\lambda \nabla \delta \lambda )\Vert _{L^2} \lesssim \Vert S_0(\nabla \lambda \delta \lambda +\lambda \nabla \delta \lambda )\Vert _{L^1} \lesssim {{\tilde{s}}}_0 \Vert \lambda \Vert _{H^s}, \end{aligned}$$

and bound their high-frequency part $S_k$ for $k>0$ by

$$\begin{aligned}&\Vert |D|^{-1}S_k(\nabla \lambda \delta \lambda +\lambda \nabla \delta \lambda )\Vert _{H^\sigma } \lesssim {{\tilde{s}}}_k\Vert \lambda \Vert _{H^s}+s_k\Vert \delta \lambda \Vert _{H^\sigma }. \end{aligned}$$

This completes the proof of (4.2).

c) Using the similar argument to *b)*, we have

$$\begin{aligned} \Vert D^2{{\mathcal {S}}}(\delta _1\psi ,\delta _2\psi )\Vert _{{\mathcal {H}}^{\sigma }} \lesssim \Vert \delta _1{{\mathcal {S}}}\Vert _{{{\mathcal {H}}}^{\sigma }}\Vert \delta _2{{\mathcal {S}}}\Vert _{{{\mathcal {H}}}^{s}} (1+\Vert \psi \Vert _{H^s})^N, \end{aligned}$$

and

$$\begin{aligned} \Vert D^2{{\mathcal {S}}}(\delta _1\psi ,\delta _2\psi )\Vert _{{\mathcal {H}}^{\sigma }} \lesssim \Vert \delta _1{{\mathcal {S}}}\Vert _{{{\mathcal {H}}}^{s}}\Vert \delta _2{{\mathcal {S}}}\Vert _{{{\mathcal {H}}}^{\sigma }} (1+\Vert \psi \Vert _{H^s})^N. \end{aligned}$$

Then by the smallness of $\psi \in H^s$, (4.2) and interpolation, the above two bounds imply

$$\begin{aligned} \Vert D^2{{\mathcal {S}}}(\delta _1\psi ,\delta _2\psi )\Vert _{{\mathcal {H}}^{\sigma }} \lesssim \Vert \delta _1\psi \Vert _{H^{\sigma _1}}\Vert \delta _2\psi \Vert _{H^{\sigma _2}}. \end{aligned}$$

This completes the proof of (4.4). $\square$

Next we establish bounds for the above solutions in space-time local energy spaces:

### Theorem 4.3

a) Assume that $\psi$ is small in $l^2{\mathbf {X}}^s$ for $s>d/2$, $d\ge 4$. Then the solution $(\lambda ,h,A,V,B)$ for the elliptic system (2.36) given by Theorem 4.1 belongs to ${\varvec{ {\mathcal {E}}}}^s$ and satisfies the bounds

4.14 $$\begin{aligned} \Vert {{\mathcal {S}}}\Vert _{{\varvec{ {\mathcal {E}}}}^s} \lesssim \Vert \psi \Vert _{l^2 {\mathbf {X}}^s}, \end{aligned}$$

with Lipschitz dependence on the initial data in these topologies. Moreover, assume that $p_k$ is an admissible frequency envelope for $\psi \in l^2{\mathbf {X}}^{s}$, we have the frequency envelope version

4.15 $$\begin{aligned} \Vert {{\mathcal {S}}}_k \Vert _{{\varvec{ {\mathcal {E}}}}^s} \lesssim p_k . \end{aligned}$$

b) In addition, for the linearization of the elliptic system (2.36) we have the bounds

4.16 $$\begin{aligned} \Vert \delta {{\mathcal {S}}}\Vert _{{\varvec{ {\mathcal {E}}}}^\sigma } \lesssim \Vert \delta \psi \Vert _{l^2 {\mathbf {X}}^\sigma }, \end{aligned}$$

for $$\sigma \in (d/2-1,s]$$.

### Proof of Theorem 4.3

For the elliptic system (4.5), we will prove the bound for differences $\delta {{\mathcal {S}}}$

4.17 $$\begin{aligned} \Vert \delta {{\mathcal {S}}}\Vert _{{\varvec{ {\mathcal {E}}}}^{\sigma }} \lesssim \Vert \delta \psi \Vert _{l^2 {\mathbf {X}}^{\sigma }}+\Vert \delta {{\mathcal {S}}}\Vert _{{\varvec{ {\mathcal {E}}}}^{\sigma }}\Vert {{\mathcal {S}}}\Vert _{{\varvec{ {\mathcal {E}}}}^{s}}\big (1+\Vert {{\mathcal {S}}}\Vert _{{\varvec{ {\mathcal {E}}}}^{s}}\big )^N. \end{aligned}$$

If this is true, by a continuity argument the bounds (4.14) and (4.16) follow.

Assume that $\tilde{s}_k$ and $s_k$ are admissible frequency envelopes for $\delta {{\mathcal {S}}}\in {\varvec{ {\mathcal {E}}}}^{\sigma }$ and ${{\mathcal {S}}}\in {\varvec{ {\mathcal {E}}}}^{s}$, respectively. We can separate the bound (4.17) into two parts, namely

$$\begin{aligned} \Vert \partial _t\delta {{\mathcal {S}}}\Vert _{L^2{{\mathcal {H}}}^{\sigma -2}} \lesssim \Vert \delta \psi \Vert _{l^2 {\mathbf {X}}^{\sigma }}(1+\Vert \partial _t\psi \Vert _{L^2 H^{s-2}}) \end{aligned}$$

respectively

4.18 $$\begin{aligned} \Vert S_k\delta {{\mathcal {S}}}\Vert _{{{\mathcal {E}}}^{\sigma }} \lesssim p_k+(\tilde{s}_k\Vert {{\mathcal {S}}}\Vert _{{\varvec{ {\mathcal {E}}}}^{s}}+s_k\Vert \delta {{\mathcal {S}}}\Vert _{{\varvec{ {\mathcal {E}}}}^{\sigma }})\big (1+\Vert {{\mathcal {S}}}\Vert _{{\varvec{ {\mathcal {E}}}}^{s}}\big )^N. \end{aligned}$$

Here one can think of the first bound as a fixed time bound for the linearization of the elliptic system (2.36), square integrated in time. As such, this is a direct consequence of the bound (4.2) with argument $\partial _t\delta \psi$ and regularity index $\sigma -2$, and the bound (4.4) with $\delta _1=\partial _t,\delta _2=\delta ,\sigma _1=s-2,\sigma _2=\sigma$ in Theorem 4.1. So it remains to prove (4.18).

If the bound (4.18) holds, then by the bound (4.3) with $\delta =\partial _t,\sigma =s-2$ and (4.18) with $\delta =Id,\sigma =s$, the bound (4.15) follows.

As an intermediate step in the proof of (4.18), we collect in the next Lemma several bilinear estimates and equivalent relations.

### Lemma 4.4

(Bilinear estimates). Let $s>d/2$, $0< \sigma \le s$, $d\ge 4$, assume that $h\in {\mathbf {Y}}^{s}$, then we have

4.19 $$\begin{aligned}&\Vert \tilde{h}h\Vert _{Y^{\sigma }}\lesssim \Vert \tilde{h}\Vert _{{\mathbf {Y}}^{\sigma }}\Vert h\Vert _{{\mathbf {Y}}^{s}}, \end{aligned}$$

4.20 $$\begin{aligned}&\Vert \lambda h\Vert _{Z^{0,\sigma }}\lesssim \Vert \lambda \Vert _{{\mathbf {Z}}^{0,\sigma }}\Vert h\Vert _{{\mathbf {Y}}^{s}}, \end{aligned}$$

4.21 $$\begin{aligned}&\Vert (Ah)\Vert _{Z^{1,\sigma }}\lesssim \Vert A\Vert _{{\mathbf {Z}}^{1,\sigma }}\Vert h\Vert _{{\mathbf {Y}}^{s}}. \end{aligned}$$

As consequences of these bounds, for $h^{\alpha {\beta }}=g^{\alpha {\beta }}-\delta ^{\alpha {\beta }},h_{\alpha {\beta }}=g_{\alpha {\beta }}-\delta _{\alpha {\beta }}$, $\lambda ^{\alpha {\beta }}=g^{\alpha \gamma }\lambda _{\gamma }^{{\beta }}, \lambda ^{{\beta }}_{\gamma }=g^{{\beta }\nu }\lambda _{\gamma \nu }$, $V^{\alpha }=g^{\alpha {\beta }}V_{{\beta }}$ and $A^{\alpha }=g^{\alpha {\beta }}A_{{\beta }}$, assume that $\Vert h_{\alpha {\beta }}\Vert _{{\mathbf {Y}}^{\sigma +1}}\ll 1$, we have

$$\begin{aligned}&\Vert h_{\alpha {\beta }}\Vert _{{\mathbf {Y}}^{\sigma +1}}\approx \Vert h^{\alpha {\beta }}\Vert _{{\mathbf {Y}}^{\sigma +1}},\\&\Vert \lambda ^{\alpha {\beta }}\Vert _{{\mathbf {Z}}^{0,\sigma }}\approx \Vert \lambda ^{{\beta }}_{\alpha }\Vert _{{\mathbf {Z}}^{0,\sigma }}\approx \Vert \lambda _{\alpha {\beta }}\Vert _{{\mathbf {Z}}^{0,\sigma }},\\&\Vert V_{\alpha }\Vert _{{\mathbf {Z}}^{1,\sigma }}\approx \Vert V^{\alpha }\Vert _{{\mathbf {Z}}^{1,\sigma }},\\&\Vert A_{\alpha }\Vert _{{\mathbf {Z}}^{1,\sigma }}\approx \Vert A^{\alpha }\Vert _{{\mathbf {Z}}^{1,\sigma }}. \end{aligned}$$

### Proof of Lemma 4.4

We do this in several steps:

*Proof of the bound* (4.19). First, we consider the *Y*-norm estimates. For the high-low interaction, for any decomposition $P_j{{\tilde{h}}}=\sum _{l\ge |j|} {{\tilde{h}}}_{j,l}$, we have

$$\begin{aligned} \Vert \sum _{l\ge |j|}( {{\tilde{h}}}_{j,l} h_{\le j})\Vert _{Y_j}\lesssim \sum _{l\ge |j|} 2^{l-|j|}\Vert ( {{\tilde{h}}}_{j,l} h_{\le j})\Vert _{l^1_lL^{\infty }L^2} \lesssim \sum _{l\ge |j|} 2^{l-|j|}\Vert {{\tilde{h}}}_{j,l}\Vert _{l^1_lL^{\infty }L^2}\Vert h_{\le j}\Vert _{L^{\infty }L^{\infty }}. \end{aligned}$$

Taking the infimum over the decomposition of ${{\tilde{h}}}_j$ yields

$$\begin{aligned} \Vert \sum _{l\ge |j|}( {{\tilde{h}}}_{j,l} h_{\le j})\Vert _{Y_j}\lesssim \Vert P_j {{\tilde{h}}}\Vert _{Y_j} \Vert h\Vert _{Z^{1,s}}, \end{aligned}$$

which is acceptable. Similarly, for the low-high interaction, we have

$$\begin{aligned} \Vert \sum _{l\ge |j|}( P_{\le j}{{\tilde{h}}}h_{j,l})\Vert _{Y^{\frac{d}{2}-1-\delta ,\sigma }}&\lesssim 2^{(\frac{d}{2}-1-\delta )j^-+\sigma j^+} \sum _{k\le j}2^{dk/2}\Vert P_{k} {{\tilde{h}}}\Vert _{L^{\infty }L^2} \Vert P_j h\Vert _{Y_j}\\&\lesssim \Vert \nabla {{\tilde{h}}}\Vert _{L^{\infty }H^{\sigma -1}}\Vert P_j h\Vert _{Y^{d/2-1-\delta ,s}}, \end{aligned}$$

which is acceptable.

Next, for the high-high interaction, when $j<0$ we rewrite it as

$$\begin{aligned} \sum _{j<j_1<-j} P_j (P_{j_1}{{\tilde{h}}}P_{j_1}h)+\sum _{-j\le j_1} P_j (P_{j_1}{{\tilde{h}}}P_{j_1}h). \end{aligned}$$

Then we bound the first term by

$$\begin{aligned}&2^{(d/2-1-\delta )j}\Vert \sum _{j<j_1<-j}P_j(P_{j_1} {{\tilde{h}}}P_{j_1}h)\Vert _{Y_j}\\&\lesssim 2^{(d-1-\delta )j}\sum _{j<j_1<-j}\Vert P_{j_1} {{\tilde{h}}}P_{j_1}h\Vert _{l^1_{|j|}L^{\infty }L^1}\\&\lesssim 2^{(d-1-\delta )j}\sum _{j<j_1<-j}\Vert P_{j_1}{{\tilde{h}}}\Vert _{l^2_{|j|}L^{\infty }L^2}\Vert P_{j_1}h\Vert _{l^2_{|j|}L^{\infty }L^2}\\&\lesssim 2^{(d-3-2\delta )j}\Vert \nabla {{\tilde{h}}}_{\le 0}\Vert _{l^2_0L^{\infty }L^2}\Vert \nabla h_{\le 0}\Vert _{l^2_0L^{\infty }L^2}+2^{(d-1-\delta )j}\Vert {{\tilde{h}}}\Vert _{Z^{1,0}}\Vert h\Vert _{Z^{1,0}}. \end{aligned}$$

We bound the second term by

$$\begin{aligned} 2^{(d/2-1-\delta )j}\Vert \sum _{-j\le j_1}P_j(P_{j_1} h P_{j_1} h)\Vert _{Y_j}&\lesssim \sum _{-j\le j_1}2^{(d-\delta )j+j_1}\Vert ( P_{j_1}\tilde{h} P_{j_1}h)\Vert _{l^1_{j_1}L^{\infty }L^1}\\&\lesssim \sum _{-j\le j_1}2^{(d-\delta )j+j_1}\Vert P_{j_1}\tilde{h}\Vert _{l^2_{j_1}L^{\infty }L^2}\Vert P_{j_1}h\Vert _{l^2_{j_1}L^{\infty }L^2}\\&\lesssim 2^{(d-\delta )j}\Vert \tilde{h}\Vert _{Z^{1,0}}\Vert h\Vert _{Z^{1,1}}. \end{aligned}$$

When $j\ge 0$, we have

$$\begin{aligned}&2^{\sigma j}\Vert \sum _{j_1>j}P_j(P_{j_1} \tilde{h} P_{j_1} h)\Vert _{Y_j}\\&\lesssim \sum _{j_1>j}2^{(\sigma -1+d/2)j+j_1}\Vert (P_{j_1} \tilde{h} P_{j_1} h)_j\Vert _{l^1_{j_1}L^{\infty }L^1}\\&\lesssim \sum _{j_1>j}2^{(\sigma -1+d/2)(j-j_1)}\Vert P_{j_1}\tilde{h}\Vert _{Z^{1,\sigma }}\Vert P_{j_1}h\Vert _{Z^{1,s}}, \end{aligned}$$

which is acceptable.

Secondly, we consider the $Z^{1,\sigma +1}$-norm estimates. For the low-frequency part, we have

$$\begin{aligned} \Vert \nabla (\tilde{h}h)_{\le 0}\Vert _{l^2_0L^{\infty }L^2}&\lesssim \Vert \nabla \tilde{h}_{\le 0}\Vert _{l^2_0L^{\infty }L^2}\Vert h_{\le 0}\Vert _{L^{\infty }L^{\infty }}+\sum _{j>0}\Vert (\tilde{h}_j h_j)_{\le 0}\Vert _{l^2_0L^{\infty }L^1}\\&\lesssim \Vert \tilde{h}\Vert _{Z^{1,0}}\Vert h\Vert _{Z^{1,s}}. \end{aligned}$$

For the high frequency part, by Littlewood-Paley dichotomy, we have

$$\begin{aligned}&2^{\sigma j}\Vert ({{\tilde{h}}}h)_j\Vert _{l^2_jL^{\infty }L^2}\\&\lesssim 2^{\sigma j}\Vert \tilde{h}_j\Vert _{l^2_jL^{\infty }L^2}\Vert h_{\le j}\Vert _{L^{\infty }L^{\infty }}+2^{\sigma j}\Vert \tilde{h}_{\le j}\Vert _{L^{\infty }L^\infty }\Vert h_{ j}\Vert _{l^2_jL^{\infty }L^2}+\sum _{l\ge j}2^{(\sigma +d/2) j}\Vert (\tilde{h}_l h_l)_j\Vert _{l^2_jL^{\infty }L^1}\\&\lesssim \Vert \tilde{h}_j\Vert _{Z^{1,\sigma }}\Vert \nabla h\Vert _{L^{\infty }H^{s-1}}+\Vert \tilde{h}\Vert _{Z^{1,\sigma }}\Vert h_j\Vert _{Z^{1,s}}+\sum _{l\ge j}2^{\sigma (j-l)}2^{(\sigma +d/2)l}\Vert \tilde{h}_l \Vert _{l^2_lL^{\infty }L^2}\Vert h_l\Vert _{L^{\infty }L^2}, \end{aligned}$$

which is acceptable. This completes the proof of (4.19).

*Proof of the bound* (4.20). First we consider the $Z^{\delta ,\sigma }$-norm estimates. For the low-frequency part we have

$$\begin{aligned} \Vert (h\lambda )_{\le 0}\Vert _{l^2_0L^{\infty }L^2}&\lesssim \Vert h_{\le 0}\Vert _{L^\infty L^\infty }\Vert \lambda _{\le 0}\Vert _{l^2_0L^{\infty }L^2}+\sum _{j>0} 2^{dj/2}\Vert h_j\Vert _{L^\infty L^2}\Vert \lambda _j\Vert _{l^2_jL^{\infty }L^2}\\&\lesssim \Vert h\Vert _{Z^{1,s}}\Vert \lambda \Vert _{Z^{0,\sigma }}. \end{aligned}$$

For the high-frequency part, by the Littlewood-Paley dichotomy, we have

$$\begin{aligned} 2^{\sigma j}\Vert (\lambda h)_j\Vert _{l^2_jL^{\infty }L^2}&\lesssim \sum _{l<j}2^{\sigma j+dl/2}\Vert \lambda _l\Vert _{L^{\infty }L^2}\Vert h_j\Vert _{l^2_jL^{\infty }L^2}+2^{\sigma j}\Vert \lambda _j\Vert _{l^2_jL^{\infty }L^2}\Vert h\Vert _{Z^{1,s}}\\&\quad +\sum _{l>j}2^{\sigma (j-l)}2^{(\sigma +d/2)l}\Vert h_l\Vert _{l^2_lL^{\infty }L^2}\Vert \lambda _l\Vert _{L^{\infty }L^2}, \end{aligned}$$

which implies

$$\begin{aligned} (\sum _{j>0}2^{2\sigma j}\Vert (h\lambda )_j\Vert _{l^2_jL^{\infty }L^2}^2)^{1/2}\lesssim \Vert h\Vert _{Z^{1,s}}\Vert \lambda \Vert _{Z^{\delta ,\sigma }}. \end{aligned}$$

This completes the proof of (4.20).

*Proof of the bound* (4.21). For the low-frequency part, by Bernstein’s inequality we have

$$\begin{aligned} \Vert \nabla (Ah)_{\le 0}\Vert _{l^2_0L^{\infty }L^2}&\lesssim \Vert \nabla (A_{\le 0}h_{\le 0})\Vert _{l^2_0L^{\infty }L^2}+\sum _{j>0}\Vert \nabla (A_jh_j)_{\le 0}\Vert _{l^2_0L^{\infty }L^2}\\&\lesssim \Vert \nabla A_{\le 0} \Vert _{l^2_0L^{\infty }L^2}\Vert \nabla h_{\le 0}\Vert _{L^{\infty }L^2}+\Vert \nabla A_{\le 0}\Vert _{L^{\infty }L^2}\Vert \nabla h_{\le 0}\Vert _{l^2_0L^{\infty }L^2}\\&\quad +\sum _{j>0} 2^{dj/2}\Vert A_j\Vert _{l^2_jL^{\infty }L^2} \Vert h_j\Vert _{L^{\infty }L^2}\\&\lesssim \Vert A \Vert _{Z^{1,0}}\Vert h\Vert _{Z^{1,s}}. \end{aligned}$$

For the high-frequency part, by Littlewood-Paley dichotomy we bound the high-low and low-high interactions by

$$\begin{aligned}&2^{\sigma k}\Vert S_k(A_k h_{<k}+A_{<k}h_k)\Vert _{l^2_kL^{\infty }L^2}\\&\lesssim 2^{\sigma k}(\Vert A_k\Vert _{l^2_kL^{\infty }L^2}\Vert h_{<k}\Vert _{L^{\infty }L^{\infty }}+\Vert A_{<k}\Vert _{L^{\infty }L^{\infty }}\Vert h_k\Vert _{l^2_kL^{\infty }L^2})\\&\lesssim \Vert A_k\Vert _{Z^{1,\sigma }}\Vert h\Vert _{Z^{1,s}}+\Vert A\Vert _{Z^{1,\sigma }}\Vert h_k\Vert _{Z^{1,s}}, \end{aligned}$$

which is acceptable. We bound the high-high interaction by

$$\begin{aligned}&2^{\sigma k}\sum _{j>k}\Vert S_k(A_j h_{j})\Vert _{l^2_kL^{\infty }L^2}\\&\lesssim \sum _{j>k} 2^{(\sigma +d/2)k}\Vert A_j h_{j}\Vert _{l^2_kL^{\infty }L^1}\\&\lesssim \sum _{j>k} 2^{\sigma (k-j)}2^{(\sigma +d/2)j}\Vert A_j \Vert _{l^2_jL^{\infty }L^2}\Vert h_j \Vert _{L^{\infty }L^2}, \end{aligned}$$

which is also acceptable. Hence, we conclude the proof of the bound (4.21). $\square$

We now turn our attention to the proof of (4.18).

*Step 1. Proof of the elliptic estimates for* $\lambda$ *equations.* By the $\lambda$-equations and Proposition 4.4, it suffices to consider the following simplified form of the equations:

$$\begin{aligned}&\partial _\alpha \delta \lambda _{\alpha {\beta }}= \partial _{\beta }\delta \psi + \delta A\psi +A \delta \psi +\delta h\nabla \lambda +h\nabla \delta \lambda +\nabla \delta h\lambda +\nabla h\delta \lambda ,\\&\partial _\alpha \delta \lambda _{{\beta }\gamma }-\partial _{\beta }\delta \lambda _{\alpha \gamma }= \delta A\lambda +A\delta \lambda +\nabla \delta h\lambda +\nabla h \delta \lambda . \end{aligned}$$

By the relation (4.13) we have for any $k>0$

$$\begin{aligned} \Vert S_k\delta \lambda \Vert _{Z^{0,\sigma }}&\lesssim \ \Vert S_k{\mathcal {R}}\delta \psi \Vert _{Z^{0,\sigma }}+\Vert S_k|D|^{-1}[\delta A(\psi +\lambda )+A (\delta \psi +\delta \lambda )\\&+\delta h\nabla \lambda +h\nabla \delta \lambda +\nabla \delta h\lambda +\nabla h\delta \lambda ] \Vert _{Z^{0,\sigma }}\\&\lesssim \ \tilde{p}_k+\tilde{s}_k \Vert {{\mathcal {S}}}\Vert _{{{\mathcal {E}}}^s}+s_k \Vert \delta {{\mathcal {S}}}\Vert _{{{\mathcal {E}}}^\sigma }. \end{aligned}$$

In order to bound the low frequency part $k=0$, we use the relation

4.22 $$\begin{aligned} f(t)=f(0)+\int _0^t \partial _s f(s)ds. \end{aligned}$$

Then we have

$$\begin{aligned} \Vert f\Vert _{l_0^2 L^{\infty }L^2}\lesssim \Vert f(0)\Vert _{L^2}+\Vert \partial _t f\Vert _{L^2L^2}. \end{aligned}$$

Using this idea, by Sobolev embeddings we have

$$\begin{aligned} \Vert S_0 \delta \lambda \Vert _{l^2_0L^{\infty }L^2}&\lesssim \ \Vert S_0{\mathcal {R}}\delta \psi \Vert _{l^2_0L^{\infty }L^2} +\Vert S_0|D|^{-1}[\delta A(\psi +\lambda )+A (\delta \psi +\delta \lambda )\\&\qquad \qquad +\delta h\nabla \lambda +h\nabla \delta \lambda +\nabla \delta h\lambda +\nabla h\delta \lambda ] \Vert _{l^2_0L^{\infty }L^2}\\&\lesssim \ \Vert S_0\delta \psi \Vert _{{\mathbf {Z}}^{0,\sigma }}\\&\ +\Vert S_0|D|^{-1}[\delta A(\psi +\lambda )+A (\delta \psi +\delta \lambda ) +\delta h\nabla \lambda \\&\qquad \qquad +h\nabla \delta \lambda +\nabla \delta h\lambda +\nabla h\delta \lambda ](0) \Vert _{L^2}\\&\qquad \qquad +\Vert S_0|D|^{-1}\partial _t [\delta A(\psi +\lambda )+A (\delta \psi +\delta \lambda )+\delta h\nabla \lambda \\&\qquad \qquad +h\nabla \delta \lambda +\nabla \delta h\lambda +\nabla h\delta \lambda ] \Vert _{L^2L^2}\\&\lesssim \ \tilde{p}_0+\tilde{s}_0\Vert {{\mathcal {S}}}\Vert _{{\varvec{ {\mathcal {E}}}}^s}. \end{aligned}$$

The high frequency part is obtained by a standard Littlewood-Paley decomposition and Bernstein inequality. This gives the elliptic estimate for the $\delta \lambda$-equation.

*Step 2. Proof of the elliptic estimates for* *V*, *A* *and* *B* *equations.* By the *V*, *A*, *B*-equations and Proposition 4.4, it suffices to consider the following form

$$\begin{aligned} \Delta V&= \ h\nabla ^2 V+\nabla h\nabla V+\nabla h\nabla h V+\lambda ^2 (A+V+\nabla h)+\lambda \nabla \lambda ,\\ \Delta A&= \ h\nabla ^2 A+\nabla h\nabla A+\nabla h\nabla h A+\lambda ^2( A+\nabla h)+\nabla (\lambda ^2),\\ \Delta B&= \ h\nabla ^2B+\nabla (\lambda \nabla \lambda +(V+A)\lambda ^2)+\lambda ^2\nabla A+\nabla h (\lambda \nabla \lambda +(V+A)\lambda ^2)\\&\quad +\nabla V\nabla A+\nabla h V\nabla A. \end{aligned}$$

The proofs of the three elliptic estimates for the above equations are similar, so we only prove the elliptic estimate for the linearization of *A*-equation in detail, i.e.

$$\begin{aligned} \Delta \delta A&= \delta h\nabla ^2 A+h\nabla ^2\delta A+\nabla \delta h\nabla A+\nabla h\nabla \delta A+\nabla \delta h\nabla h A+(\nabla h)^2\delta A\\&\quad +\delta \lambda \lambda (A+\nabla h)+\lambda ^2 (\delta A+\nabla \delta h)+\nabla \lambda \delta \lambda +\lambda \nabla \delta \lambda . \end{aligned}$$

We bound all the nonlinearities except $\nabla \lambda \delta \lambda$ and $\lambda \nabla \delta \lambda$ by

$$\begin{aligned}&\Vert |D|^{-2}S_k(\delta h\nabla ^2 A+h\nabla ^2\delta A+\nabla \delta h\nabla A+\nabla h\nabla \delta A+\nabla \delta h\nabla h A+(\nabla h)^2\delta A)\Vert _{Z^{1,\sigma +1}}\\&\quad +\Vert |D|^{-2}S_k(\delta \lambda \lambda (A+\nabla h)+\lambda ^2 (\delta A+\nabla \delta h))\Vert _{Z^{1,\sigma +1}}\\&\lesssim \ (\tilde{s}_k\Vert {{\mathcal {S}}}\Vert _{{\varvec{ {\mathcal {E}}}}^{s}}+s_k\Vert \delta {{\mathcal {S}}}\Vert _{{\varvec{ {\mathcal {E}}}}^{\sigma }} ) (1+\Vert {{\mathcal {S}}}\Vert _{{\varvec{ {\mathcal {E}}}}^{s}})^N, \end{aligned}$$

for $\sigma \in (d/2-1,s]$. All terms are estimated in a similar fashion, so we only bound the first term $\delta h\nabla ^2 A$.

For the low-frequency part we use the relation (4.22) to bound the second term $\delta h\nabla ^2A$ by

$$\begin{aligned}&\Vert \nabla ^{-1}(\delta h\nabla ^2A)_{\le 0}\Vert _{l_0^2L^{\infty }L^2}\\&\lesssim \ \Vert \nabla ^{-1}(\delta h\nabla ^2 A)_{\le 0}(0)\Vert _{L^2}+\Vert \nabla ^{-1}\partial _t(\delta h\nabla ^2 A)_{\le 0}\Vert _{L^2L^2}\\&\lesssim \ \Vert (\delta h\nabla ^2 A)_{\le 0}(0)\Vert _{L^{2d/(d+2)}} +\quad \Vert \partial _t(\delta h\nabla ^2 A)_{\le 0}\Vert _{L^2L^{2d/(d+2)}}\\&\lesssim \ \Vert \delta h\Vert _{Z^{1,1}}\Vert A\Vert _{Z^{1,1}}+\Vert \nabla \partial _t \delta h\Vert _{L^2H^{\sigma -1}}\Vert A\Vert _{Z^{1,s+1}}+\Vert \delta h\Vert _{Z^{1,\sigma +2}}\Vert \nabla \partial _t A\Vert _{L^2H^{s-3}}\\&\lesssim \ \Vert \delta h\Vert _{{\mathbf {Z}}^{1,\sigma +2}}\Vert A\Vert _{{\mathbf {Z}}^{1,s+1}}. \end{aligned}$$

A minor modification of this argument also yields

$$\begin{aligned} \Vert \nabla ^{-1}(\delta h\nabla ^2 A)_{\le 0}\Vert _{l_0^2L^{\infty }L^2} \lesssim \tilde{s}_0\Vert {{\mathcal {S}}}\Vert _{{\varvec{ {\mathcal {E}}}}^{s}}. \end{aligned}$$

For the high-frequency part, by Littlewood-Paley dichotomy and Bernstein’s inequality (3.1), we have

$$\begin{aligned}&2^{\sigma j}\Vert |D|^{-1}(\delta h\nabla ^2A)_j\Vert _{l^2_jL^{\infty }L^2}\\&\lesssim 2^{(\sigma -1)j}(\Vert \delta h_{<j}\nabla ^2 A_j\Vert _{l^2_jL^{\infty }L^2}+\Vert \delta h_{j}\nabla ^2A_{<j}\Vert _{l^2_jL^{\infty }L^2}+\sum _{l>j}\Vert \delta h_{l}\nabla ^2A_l\Vert _{l^2_jL^{\infty }L^2})\\&\lesssim \Vert \delta h\Vert _{L^{\infty }L^{\infty }}2^{(\sigma +1) j}\Vert A_j\Vert _{l^2_jL^{\infty }L^2}+\sum _{l<j} 2^{(l-j)+\sigma j}\Vert \delta h_j\Vert _{l^2_jL^{\infty }L^2}\Vert \nabla ^{d/2+1}A_l\Vert _{L^{\infty }L^2}\\&\quad +\sum _{l>j}2^{(\sigma -1)j}2^{(d/2+2)l}\Vert \delta h_l\Vert _{L^{\infty }L^2}\Vert A_l\Vert _{l^2_lL^{\infty }L^2}\\&\lesssim s_j\Vert \delta {{\mathcal {S}}}\Vert _{{\varvec{ {\mathcal {E}}}}^{\sigma }}+ \tilde{s}_j\Vert {{\mathcal {S}}}\Vert _{{\varvec{ {\mathcal {E}}}}^{s}}. \end{aligned}$$

Finally, we bound the last two terms $\nabla \lambda \delta \lambda$ and $\lambda \nabla \delta \lambda$. For low-frequency part, using $d\ge 4$ we have

$$\begin{aligned}&\Vert |D|^{-1}(\nabla \lambda \delta \lambda )_{\le 0}\Vert _{l^2_0L^{\infty }L^2}\\&\lesssim \Vert |D|^{-1}(\nabla \lambda \delta \lambda )_{\le 0}(0)\Vert _{L^2}+\Vert |D|^{-1}\partial _t(\nabla \lambda \delta \lambda )_{\le 0}\Vert _{L^2L^2}\\&\lesssim \Vert (\nabla \lambda \delta \lambda )_{\le 0}(0)\Vert _{L^1}+\Vert \partial _t(\nabla \lambda \delta \lambda )_{\le 0}\Vert _{L^2L^1}\\&\lesssim \Vert \delta \lambda \Vert _{{\mathbf {Z}}^{0,\sigma }}\Vert \lambda \Vert _{{\mathbf {Z}}^{0,s}}. \end{aligned}$$

We also obtain

$$\begin{aligned} \Vert |D|^{-1}(\nabla \lambda \delta \lambda )_{\le 0}\Vert _{l^2_0L^{\infty }L^2} \lesssim \tilde{s}_0 \Vert {{\mathcal {S}}}\Vert _{{\varvec{ {\mathcal {E}}}}^{s}}. \end{aligned}$$

For the high-frequency part, we have

$$\begin{aligned} \Vert \Delta ^{-1}(\nabla \lambda \delta \lambda )_j\Vert _{Z^{1,\sigma +1}}\lesssim \tilde{s}_j \Vert {{\mathcal {S}}}\Vert _{{\varvec{ {\mathcal {E}}}}^{s}}+s_j \Vert \delta {{\mathcal {S}}}\Vert _{{\varvec{ {\mathcal {E}}}}^{\sigma }}. \end{aligned}$$

We can also bound the term $\lambda \nabla \delta \lambda$ similarly. This gives the elliptic estimate for $\delta A$-equation.

*Step 3. Proof of the elliptic estimate for* *h*-*equation.* By *h*-equation in (4.5) and Proposition 4.4, it suffices to consider a more general equation of the form

$$\begin{aligned} \Delta \delta h=\delta h\nabla ^2 h+h\nabla ^2 \delta h+\nabla \delta h\nabla h+\delta h\nabla h\nabla h+h\nabla h\nabla \delta h+\delta \lambda \lambda . \end{aligned}$$

The proof of the $Z^{1,\sigma +2}$ bound is similar to the estimates for *V*, *A*, *B* equations in *Step 2*, hence we only bound of the $Y^{d/2-1-\delta ,\sigma +2}$-norm. We prove that the following frequency envelope version holds:

$$\begin{aligned} \Vert S_j \delta h\Vert _{Y^{d/2-1-\delta ,\sigma +2}}\lesssim (\tilde{s}_j \Vert {{\mathcal {S}}}\Vert _{{\varvec{ {\mathcal {E}}}}^{s}}+s_j \Vert \delta {{\mathcal {S}}}\Vert _{{\varvec{ {\mathcal {E}}}}^{\sigma }})(1+\Vert {{\mathcal {S}}}\Vert _{{\varvec{ {\mathcal {E}}}}^{s}})^N. \end{aligned}$$

*Case 1. The contribution of* $\delta \lambda \lambda$. By the Littlewood-Paley dichotomy, it suffices to consider the high-low, low-high and high-high cases for any $j\in {{\mathbb {Z}}}$

$$\begin{aligned} \sum _{l<j+O(1)}P_j(P_j\delta \lambda P_{l}\lambda ),\quad \sum _{l<j+O(1)}P_j(P_{l}\delta \lambda P_j \lambda ),\quad \sum _{l>j+O(1)}P_j(P_{l}\delta \lambda P_{l}\lambda ). \end{aligned}$$

*Case 1(a). The contribution of high-low and low-high interaction.* The two cases are proved similarly, so we only consider the worst case, namely the low-high interaction. When $j\le 0$, by the definition of the $Y_j$-norm we have

$$\begin{aligned} 2^{(d/2-1-\delta )j}\Vert \Delta ^{-1}\sum _{l<j}P_j(P_l\delta \lambda \cdot P_{j}\lambda )\Vert _{Y_j}&\lesssim \ 2^{(d/2-3-\delta )j}\sum _{l<j}\Vert (P_l\delta \lambda \cdot P_{j}\lambda )\Vert _{l^1_{|l|}L^{\infty }L^2} \\&\lesssim 2^{(d-3-\delta )j}\sum _{l<j} \Vert P_l\delta \lambda \Vert _{l^2_{|l|}L^{\infty }L^2} \Vert P_j\lambda \Vert _{l^2_{|l|}L^{\infty }L^2}\\&\lesssim 2^{(d-3-3\delta )j} \Vert |D|^{\delta }\delta \lambda _{\le 0}\Vert _{l^2_0L^{\infty }L^2}\Vert |D|^{\delta }\lambda _{\le 0}\Vert _{l^2_0L^{\infty }L^2}\\&\lesssim 2^{(d-3-3\delta )j} \tilde{s}_0\Vert {{\mathcal {S}}}\Vert _{{\varvec{ {\mathcal {E}}}}^{s}}. \end{aligned}$$

When $j>0$, we further divide the low-high interaction into

$$\begin{aligned} \sum _{l<j}P_j(P_l\delta \lambda \cdot P_j\lambda )=\sum _{-j\le l<j}P_j(P_l\delta \lambda \cdot P_j\lambda )+\sum _{l<-j}P_j(P_l\delta \lambda \cdot P_j\lambda ). \end{aligned}$$

For the first term, by Bernstein’s inequality we have

$$\begin{aligned} 2^{(\sigma +2)j}\Vert \Delta ^{-1}\sum _{-j\le l<j}P_j(P_l\delta \lambda \cdot P_j\lambda )\Vert _{Y_j}&\lesssim \ 2^{\sigma j}\sum _{-j\le l\le j} \Vert P_l\delta \lambda \cdot P_j\lambda \Vert _{l^1_j L^{\infty }L^2}\\&\lesssim \ 2^{\sigma j}\sum _{-j\le l\le j} 2^{dl/2}\Vert P_l\delta \lambda \Vert _{l^2_j L^{\infty }L^2}\Vert P_j\lambda \Vert _{l^2_j L^{\infty }L^2} \\&\lesssim \ s_j \Vert \delta {{\mathcal {S}}}\Vert _{{\varvec{ {\mathcal {E}}}}^{\sigma }}. \end{aligned}$$

For the second term we have

$$\begin{aligned}&2^{(\sigma +2)j}\Vert \Delta ^{-1}\sum _{l<-j}P_j(P_l\delta \lambda \cdot P_j\lambda )\Vert _{Y_j}\\&\quad \lesssim \ 2^{\sigma j}\sum _{l<-j} 2^{|l|-j}\Vert P_l\delta \lambda \cdot P_j\lambda \Vert _{l^1_{|l|}L^{\infty }L^2} \\&\quad \lesssim 2^{(\sigma -1)j}\sum _{l<-j} 2^{(d/2-1)l}\Vert P_l\delta \lambda \Vert _{l^2_{|l|}L^{\infty }L^2} \Vert P_j\lambda \Vert _{l^2_{|l|}L^{\infty }L^2}\\&\quad \lesssim \Vert |D|^{\delta }\delta \lambda _{\le 0} \Vert _{l^2_0L^{\infty }L^2} 2^{(\sigma -1)j} \Vert P_j\lambda \Vert _{l^2_jL^{\infty }L^2}\\&\quad \lesssim s_j \Vert \delta {{\mathcal {S}}}\Vert _{{\varvec{ {\mathcal {E}}}}^{\sigma }}. \end{aligned}$$

*Case 1(b). The contribution of high-high interactions.* When $j<0$, we divide this into

$$\begin{aligned} \sum _{l>j} P_j(P_l\delta \lambda \cdot P_l\lambda )=\sum _{-j\ge l>j} P_j(P_l\delta \lambda \cdot P_l\lambda )+\sum _{l>-j} P_j(P_l\delta \lambda \cdot P_l\lambda ). \end{aligned}$$

Then we bound the first term by

$$\begin{aligned}&2^{(d/2-1-\delta )j}\Vert \Delta ^{-1}\sum _{-j\ge l>j} P_j(P_l\delta \lambda \cdot P_l\lambda )\Vert _{Y_j}\\&\lesssim 2^{(d-3-\delta )j}\sum _{-j\ge l>j}\Vert P_l\delta \lambda \cdot P_l\lambda \Vert _{l^1_{|j|}L^{\infty }L^1}\\&\lesssim 2^{(d-3-2\delta )j}(\sum _{0\ge l>j}2^{\delta l}\Vert \delta \lambda _{\le 0}\Vert _{l^2_0L^{\infty }L^2}\Vert \lambda _{\le 0}\Vert _{l^2_0L^{\infty }L^2}+\sum _{-j\ge l>0}\Vert \delta \lambda _{l}\Vert _{l^2_{l}L^{\infty }L^2}\Vert \lambda _{l}\Vert _{l^2_{l}L^{\infty }L^2})\\&\lesssim 2^{(d-3-2\delta )j} \tilde{s}_0\Vert {{\mathcal {S}}}\Vert _{{\varvec{ {\mathcal {E}}}}^{s}}. \end{aligned}$$

Using the $Y_j$ norm we can also bound the second term by

$$\begin{aligned} 2^{(d/2-1-\delta )j}\Vert \Delta ^{-1}\sum _{l>-j}P_j(P_l\delta \lambda \cdot P_l\lambda )\Vert _{Y_j}&\lesssim 2^{(d-3-\delta )j}\sum _{l>-j}2^{l-|j|}\Vert (P_l\delta \lambda \cdot P_l\lambda )\Vert _{l^1_{l}L^{\infty }L^1}\\&\lesssim 2^{(d-2-\delta )j}\sum _{l>-j}2^{l}\Vert P_l\delta \lambda \Vert _{l^2_{l}L^{\infty }L^2}\Vert P_l\lambda \Vert _{l^2_{l}L^{\infty }L^2}\\&\lesssim 2^{(d-2-4\delta )j} \tilde{s}_0\Vert {{\mathcal {S}}}\Vert _{{\varvec{ {\mathcal {E}}}}^{s}}. \end{aligned}$$

Finally, when $j>0$, using again the $Y_j$ norm we have

$$\begin{aligned}&2^{(\sigma +2)j}\Vert \Delta ^{-1}\sum _{l>j}P_j(P_l\delta \lambda \cdot P_l\lambda )\Vert _{Y_j}\\&\quad \lesssim 2^{(\sigma +d/2)j}\sum _{l>j}2^{l-j}\Vert (\delta \lambda _{l}\cdot \lambda _{l})_j\Vert _{l^1_{l}L^{\infty }L^1}\\&\quad \lesssim \sum _{j_1>j}2^{(\sigma +d/2-1)(j-l)}2^{(\sigma +d/2)l}\Vert \delta \lambda _{l}\Vert _{l^2_{l}L^{\infty }L^2}\Vert \lambda _{l}\Vert _{l^2_{l}L^{\infty }L^2}\\&\quad \lesssim \tilde{s}_j \Vert {{\mathcal {S}}}\Vert _{{\varvec{ {\mathcal {E}}}}^{s}}. \end{aligned}$$

*Case 2. The contribution of* $\delta h\nabla ^2 h$, $h\nabla ^2 \delta h$ *and* $\nabla \delta h\nabla h$. It suffices to prove that

$$\begin{aligned} \Vert \Delta ^{-1}S_j(\delta h\nabla ^2 h+\nabla ^2\delta h\cdot h+\nabla \delta h\nabla h)\Vert _{Y^{d/2-1-\delta ,\sigma +2}}\lesssim \tilde{s}_j \Vert {{\mathcal {S}}}\Vert _{{\varvec{ {\mathcal {E}}}}^{s}}+s_j \Vert \delta {{\mathcal {S}}}\Vert _{{\varvec{ {\mathcal {E}}}}^{\sigma }}. \end{aligned}$$

For the high-low interactions, it suffices to consider the worst case $\nabla ^2 P_j\delta h\cdot P_{\le j} h$. For any decomposition $P_j\delta h=\sum _{l\ge |j|} \delta h_{j,l}$, we have

$$\begin{aligned} \Vert \Delta ^{-1}\sum _{l\ge |j|}(\nabla ^2 \delta h_{j,l} P_{\le j}h )\Vert _{Y_j}&\lesssim \sum _{l\ge |j|} 2^{l-|j|-2j}\Vert (\nabla ^2 \delta h_{j,l} P_{\le j}h )\Vert _{l^1_lL^{\infty }L^2}\\&\lesssim \sum _{l\ge |j|} 2^{l-|j|}\Vert \delta h_{j,l}\Vert _{l^1_lL^{\infty }L^2}\Vert P_{\le j}h\Vert _{L^{\infty }L^{\infty }} \end{aligned}$$

Taking the infimum over the decomposition of $P_jh$ yields

$$\begin{aligned} \Vert \Delta ^{-1}(\nabla ^2P_j \delta hP_{\le j}h)\Vert _{Y_j}\lesssim \Vert P_j\delta h\Vert _{Y_j} \Vert P_{\le j}h\Vert _{L^{\infty }L^{\infty }}, \end{aligned}$$

which is acceptable. The low-high interactions is similar and omitted.

For the high-high interaction, it suffices to estimate $\sum _{l>j}P_j(P_l\nabla \delta hP_l\nabla h)$. By Bernstein’s inequality we have

$$\begin{aligned}&2^{(d/2-1-\delta )j^-+(\sigma +2)j^+}\Vert \Delta ^{-1}\sum _{l>j}P_j(P_l\nabla \delta hP_l\nabla h)\Vert _{Y_j}\\&\lesssim 2^{(d-3-\delta )j^-+(\sigma +d/2)j^+}\sum _{l>j}\Vert P_j(\nabla P_l\delta hP_l\nabla h)\Vert _{l^1_{|j|}L^{\infty }L^1}\\&\lesssim 2^{(d-3-\delta )j^-+(\sigma +d/2)j^+}\sum _{l>j}\Vert P_l\nabla \delta h\Vert _{l^2_{|j|}L^{\infty }L^2}\Vert P_l\nabla h\Vert _{l^2_{|j|}L^{\infty }L^2}\\&\lesssim 2^{(d-3-2\delta )j^-}(\Vert \nabla \delta h_{\le 0}\Vert _{l^2_0L^{\infty }L^2}\Vert \nabla h_{\le 0}\Vert _{l^2_0L^{\infty }L^2}+\sum _{l>0}2^{dl}\Vert \nabla \delta h_{l}\Vert _{l^2_{l}L^{\infty }L^2}\Vert \nabla h_{l}\Vert _{l^2_{l}L^{\infty }L^2})\\&\quad +\sum _{l>j,j>0}2^{(\sigma -d/2)(j-l)}2^{(\sigma +d/2)l}\Vert \nabla \delta h_{l}\Vert _{l^2_{l}L^{\infty }L^2}\Vert \nabla h_{l}\Vert _{l^2_{l}L^{\infty }L^2}\\&\lesssim 2^{(d-3-2\delta )j^-} \tilde{s}_0 \Vert {{\mathcal {S}}}\Vert _{{\varvec{ {\mathcal {E}}}}^{s}}+\tilde{s}_j \Vert {{\mathcal {S}}}\Vert _{{\varvec{ {\mathcal {E}}}}^{s}}. \end{aligned}$$

*Case 3. The contribution of* $\delta h\nabla h\nabla h$ *and* $h\nabla h\nabla \delta h$. It suffices to prove that

$$\begin{aligned} \Vert \Delta ^{-1}S_j(\delta h\nabla h\nabla h+h\nabla h\nabla \delta h)\Vert _{Y^{d/2-1-\delta ,\sigma +2}}\lesssim \tilde{s}_j\Vert {{\mathcal {S}}}\Vert _{{\varvec{ {\mathcal {E}}}}^{s}}^2. \end{aligned}$$

For the low-frequency part, By Bernstein’s inequality and $d\ge 4$ we have

$$\begin{aligned}&\Vert \Delta ^{-1}(\delta h\nabla h\nabla h)_{\le 0}\Vert _{Y^{d/2-1-\delta ,\sigma +2}}\\&\lesssim \Vert (\delta h\nabla h\nabla h)_{\le 0}\Vert _{l^1_0L^{\infty }L^1}\\&\lesssim \Vert \delta h_{\le 0}\Vert _{L^{\infty }L^{\infty }}\Vert (\nabla h\nabla h)_{\le 0}\Vert _{l^1_0L^{\infty }L^1}+\sum _{j>0}\Vert \delta h_j\Vert _{l^2_0L^{\infty }L^2}\Vert (\nabla h\nabla h)_j\Vert _{l^2_0L^{\infty }L^2}\\&\lesssim \tilde{s}_0\Vert {{\mathcal {S}}}\Vert _{{\varvec{ {\mathcal {E}}}}^{s}}^2. \end{aligned}$$

For the high-frequency part, by Bernstein’s inequality we also have

$$\begin{aligned} 2^{(\sigma +2)j}\Vert \Delta ^{-1}(\delta h\nabla h\nabla h)_j\Vert _{Y_j} \lesssim 2^{\sigma j}\Vert (\delta h\nabla h\nabla h)_j\Vert _{l^1_jL^{\infty }L^2} \lesssim \tilde{s}_j\Vert {{\mathcal {S}}}\Vert _{{\varvec{ {\mathcal {E}}}}^{s}}^2. \end{aligned}$$

Thus this completes the proof of $Y^{d/2-1-\delta ,\sigma +2}$ bound. $\square$

## Multilinear and Nonlinear Estimates

This section contains our main multilinear estimates which are needed for the analysis of the Schrödinger equation in (2.35). We begin with the following low-high bilinear estimates of $\nabla h\nabla \psi$.

### Lemma 5.1

Let $s>\frac{d}{2}$, $d\ge 2$ and $k\in {{\mathbb {N}}}$. Suppose that $\nabla a(x)\lesssim \langle x\rangle ^{-1}$, $h\in {\mathbf {Y}}^{\sigma +2}$ and $\psi _k\in l^2X^s$. Then for $-s\le \sigma \le s$ we have

5.1 $$\begin{aligned}&\Vert \nabla h_{\le k}\cdot \nabla \psi _k\Vert _{l^2N^{\sigma }}\lesssim \min \{ \Vert h\Vert _{{\mathbf {Y}}^{\sigma +2}}\Vert \psi _k\Vert _{l^2X^s},\Vert h\Vert _{{\mathbf {Y}}^{s+2}}\Vert \psi _k\Vert _{l^2X^{\sigma }}\}, \end{aligned}$$

5.2 $$\begin{aligned}&\Vert h_{\le k}\nabla a\nabla \psi _k\Vert _{l^2N^{\sigma }}\lesssim \min \{ \Vert h\Vert _{{\mathbf {Y}}^{\sigma +2}}\Vert \psi _k\Vert _{l^2X^s},\Vert h\Vert _{{\mathbf {Y}}^{s+2}}\Vert \psi _k\Vert _{l^2X^{\sigma }}\}. \end{aligned}$$

In addition, if $-s\le \sigma \le s-1$ then we have

5.3 $$\begin{aligned} \Vert h_{\le k}\nabla ^2\psi _k\Vert _{l^2N^{\sigma }}\lesssim \Vert h\Vert _{{\mathbf {Y}}^{\sigma +2}}\Vert \psi _k\Vert _{l^2X^{s}}. \end{aligned}$$

### Proof

*a) The estimates* (5.1) *and* (5.3). The proof of second bound (5.3) is similar to the first, so we only prove the first bound in detail. By duality, it suffices to estimate

$$\begin{aligned} I_j\,{:}{=}\,\langle \nabla P_j h\nabla \psi _k,z_k\rangle ,\quad j\le k,\ j\in {{\mathbb {Z}}},\ k\in {\mathbb {N}}, \end{aligned}$$

for any $z_k\,{:}{=}\,S_kz\in l_k^2X_k$ with $\Vert z_k\Vert _{l^2_kX_k}\le 1$. For $I_j$ and any decomposition $P_jh=\sum _{l\ge |j|}h_{j,l}$, by duality and Bernstein inequality, we have

$$\begin{aligned} I_j&\lesssim \sum _{l\ge |j|} \sup _{\Vert z_k\Vert _{l^2_k X_k}\le 1}\langle \nabla h_{j,l}\nabla \psi _k,z_k\rangle \\&\lesssim \sum _{l\ge |j|} \sup _{\Vert z_k\Vert _{l^2_k X_k}\le 1}\Vert \nabla h_{j,l}\Vert _{l^1_l L^{\infty }L^{\infty }} \Vert \nabla \psi _k\Vert _{l^{\infty }_lL^2L^2}\Vert z_k\Vert _{l^{\infty }_lL^2L^2}\\&\lesssim \sum _{l\ge |j|} 2^l\Vert \nabla h_{j,l}\Vert _{l^1_l L^{\infty }L^{\infty }} \Vert \psi _k\Vert _{X_k}\\&\lesssim 2^{(\frac{d}{2}+1)j+|j|}\sum _{l\ge |j|}2^{l-|j|}\Vert h_{j,l}\Vert _{l^1_lL^{\infty }L^2}\Vert \psi _k\Vert _{X_k}. \end{aligned}$$

Then taking the infimum over the decomposition of $P_jh$ and incorporating the summation over *j* yield

$$\begin{aligned} \sum _{j\le k} 2^{\sigma k}I_j&\lesssim \Vert h\Vert _{{\mathbf {Y}}^{d/2+2+\epsilon }}\Vert \psi _k\Vert _{X^{\sigma }}, \end{aligned}$$

for any $\epsilon >0$. If $-s\le \sigma \le d/2$, we also have

$$\begin{aligned} \sum _{j\le k} 2^{\sigma k}I_j&\lesssim \sum _{j\le 0}2^{dj/2}\Vert P_j h\Vert _{Y_j}\Vert \psi _k\Vert _{X^{\sigma }}+\sum _{j> 0}2^{(d/2+\epsilon -\sigma )(j-k)}2^{(\sigma +2)j}\Vert P_j h\Vert _{Y_j}\Vert \psi _k\Vert _{X^{s}}\\&\lesssim \Vert h\Vert _{{\mathbf {Y}}^{\sigma +2}}\Vert \psi _k\Vert _{X^{s}}. \end{aligned}$$

Thus the bound (5.1) follows.

*Estimate* (5.2). By duality, it suffices to bound

$$\begin{aligned} II_j= \langle P_jh\nabla a\nabla \psi _k,z_k\rangle ,\ \ j\le k,\ j\in {{\mathbb {Z}}}, \end{aligned}$$

for any $z_k\in l^2_k X_k$ with $\Vert z_k\Vert _{l^2_k X_k}\le 1$. For any decomposition $P_jh=\sum _{l\ge |j|}h_{j,l}$, by $|\nabla a|(x)\lesssim \langle x\rangle ^{-1}$, we consider the two cases $|x|\ge 2^{j/2}$ and $|x|<2^{j/2}$ respectively and then obtain

$$\begin{aligned} II_j&\lesssim \sum _{l\ge |j|} \sup _{\Vert z_k\Vert _{l^2_k X_k}\le 1}\langle h_{j,l}\langle x\rangle ^{-1}{\mathbf {1}}_{\le 2^{l/2}}(x)\nabla \psi _k,z_k\rangle \\&\quad +\sum _{l\ge |j|} \sup _{\Vert z_k\Vert _{l^2_k X_k}\le 1}\langle h_{j,l}\langle x\rangle ^{-1}{\mathbf {1}}_{>2^{l/2}}(x)\nabla \psi _k,z_k\rangle \\&= II_{j1}+II_{j2}. \end{aligned}$$

The first term is bounded by

$$\begin{aligned} II_{j1}&\lesssim \sum _{l\ge |j|} \sup _{\Vert z_k\Vert _{l^2_k X_k}\le 1}\Vert h_{j,l}\Vert _{l^1_l L^{\infty }L^{\infty }} \Vert \nabla \psi _k\Vert _{l^{\infty }_{l/2}L^2L^2}\Vert z_k\Vert _{l^{\infty }_{l/2}L^2L^2}\\&\lesssim \sum _{l\ge |j|} 2^{l/2}\Vert h_{j,l}\Vert _{l^1_l L^{\infty }L^{\infty }} \Vert \psi _k\Vert _{X_k}\\&\lesssim 2^{dj/2+|j|/2}\sum _{l\ge |j|} 2^{l-|j|}\Vert h_{j,l}\Vert _{l^1_l L^{\infty }L^2} \Vert \psi _k\Vert _{X_k} \end{aligned}$$

The second term is bounded by

$$\begin{aligned} II_{j2}&\lesssim \sum _{l\ge |j|} 2^{-l/2}\sup _{\Vert z_k\Vert _{l^2_k X_k}\le 1}\Vert h_{j,l}\Vert _{l^1_l L^{\infty }L^{\infty }} \Vert \nabla \psi _k\Vert _{l^{\infty }_{l}L^2L^2}\Vert z_k\Vert _{l^{\infty }_{l}L^2L^2}\\&\lesssim \sum _{l\ge |j|} 2^{l/2}\Vert h_{j,l}\Vert _{l^1_l L^{\infty }L^{\infty }} \Vert \psi _k\Vert _{X_k}\\&\lesssim 2^{dj/2+|j|/2}\sum _{l\ge |j|} 2^{l-|j|}\Vert h_{j,l}\Vert _{l^1_l L^{\infty }L^2} \Vert \psi _k\Vert _{X_k}. \end{aligned}$$

Then we obtain

$$\begin{aligned} \sum _{j\le k} 2^{\sigma k}II_j&\lesssim (\sum _{j\le 0}2^{(d-1)j/2}\Vert P_j h\Vert _{Y_j}+\sum _{j>0}2^{(d+1)j/2}\Vert h_j\Vert _{Y_j})\Vert \psi _k\Vert _{X^{\sigma }}\\&\lesssim \min \{ \Vert h\Vert _{{\mathbf {Y}}^{\sigma +2}}\Vert \psi _k\Vert _{l^2X^s},\Vert h\Vert _{{\mathbf {Y}}^{s+2}}\Vert \psi _k\Vert _{l^2X^{\sigma }}\}. \end{aligned}$$

Thus the bound (5.2) follows. $\square$

We next prove the remaining bilinear estimates and trilinear estimates.

### Proposition 5.2

(Nonlinear estimates). a) Let $s>\frac{d}{2}$ and $d\ge 3$, assume that $p_k$ and $s_k$ are admissible frequency envelopes for $\psi \in l^2X^{s}$, ${{\mathcal {S}}}\in {{\mathcal {E}}}^{s}$ respectively. Then we have

5.4 $$\begin{aligned}&\Vert S_k(B\psi )\Vert _{l^2N^{s}}\lesssim s_k\Vert \psi \Vert _{l^2 X^{s}}+p_k\Vert B\Vert _{Z^{1,s}} , \end{aligned}$$

5.5 $$\begin{aligned}&\Vert S_k(A^2\psi )\Vert _{l^2N^{s}}\lesssim s_k\Vert A\Vert _{Z^{1,s}}\Vert \psi \Vert _{l^2X^{s}}+ p_k\Vert A\Vert _{Z^{1,s}}^2, \end{aligned}$$

5.6 $$\begin{aligned}&\Vert S_k(\lambda ^3)\Vert _{l^2N^{s}}\lesssim s_k \Vert \lambda \Vert _{Z^{0,s}}^2. \end{aligned}$$

b) Assume that $\tilde{p}_k$ and $\tilde{s}_k$ are admissible frequency envelopes for $\psi \in l^2X^{\sigma }$, ${{\mathcal {S}}}\in {{\mathcal {E}}}^{\sigma }$ respectively. Then for $-s\le \sigma \le s$ we have

5.7 $$\begin{aligned}&\Vert S_k\nabla (h_{\ge k-4}\nabla \psi )\Vert _{l^2N^{\sigma }}\lesssim \min \{\tilde{s}_k\Vert \psi \Vert _{l^2X^{s}},\tilde{p}_k\Vert h\Vert _{Z^{1,s+2}}\}, \end{aligned}$$

5.8 $$\begin{aligned}&\Vert S_k(A_{\ge k-4}\nabla \psi )\Vert _{l^2N^{\sigma }}\lesssim \min \{\tilde{s}_k\Vert \psi \Vert _{l^2X^{s}},\tilde{p}_k\Vert A\Vert _{Z^{1,s+1}}\}, \end{aligned}$$

and for $-s\le \sigma \le s-\delta$ we have

5.9 $$\begin{aligned}&\Vert S_k(B\psi )\Vert _{l^2N^{\sigma }}\lesssim \min \{\tilde{s}_k\Vert \psi \Vert _{l^2 X^{s}},\tilde{p}_k\Vert B\Vert _{Z^{1,s}} \}, \end{aligned}$$

5.10 $$\begin{aligned}&\Vert S_k(A^2\psi )\Vert _{l^2N^{\sigma }}\lesssim \min \{\tilde{s}_k\Vert A\Vert _{Z^{1,s}}\Vert \psi \Vert _{l^2X^{s}}, \tilde{p}_k\Vert A\Vert _{Z^{1,s}}^2\}, \end{aligned}$$

5.11 $$\begin{aligned}&\Vert S_k(\lambda ^3)\Vert _{l^2N^{\sigma }}\lesssim \tilde{s}_k \Vert \lambda \Vert _{Z^{0,s}}^2. \end{aligned}$$

If $$-s\le \sigma \le s-1$$, then

5.12 $$\begin{aligned} \Vert S_k(A_{< k-4}\nabla \psi )\Vert _{l^2N^{\sigma }}\lesssim p_k\Vert A\Vert _{Z^{1,\sigma +1}}. \end{aligned}$$

### Proof

We first prove (5.7) and (5.8). These two bounds are proved similarly, here we only prove the first bound in detail. For the high-low case, by (3.1) we have

$$\begin{aligned} \sum _{j_2\le k+C}\Vert S_k \nabla ( h_{j_1}\nabla \psi _{j_2})\Vert _{l^2N^{\sigma }}&\lesssim \sum _{j_1=k+O(1),j_2\le k+C} 2^{(\sigma +1) k}\Vert h_{j_1}\Vert _{l_k^2L^2L^2}\Vert \nabla \psi _{j_2}\Vert _{l^2_kL^{\infty }L^{\infty }}\\&\lesssim \sum _{j_2\le k+C} 2^{\sigma k+(d/2+1)j_2}\Vert \nabla h_k\Vert _{L^2L^2}\Vert \psi _{j_2}\Vert _{l_{j_2}^2L^{\infty }L^2}\\&\lesssim \min \{\tilde{s}_k\Vert \psi \Vert _{l^2X^{s}},\tilde{p}_k\Vert h\Vert _{Z^{1,s+2}}\}. \end{aligned}$$

For the high-high case, when $\sigma +d/2+1>\delta$ we have

$$\begin{aligned}&\sum _{j_1= j_2+O(1),j_1>k}\Vert S_k \nabla ( h_{j_1}\nabla \psi _{j_2})\Vert _{l^2N^{\sigma }}\\&\lesssim \sum _{j_1= j_2+O(1),j_1>k}2^{(\sigma +1) k+dk/2}\Vert S_k ( h_{j_1}\nabla \psi _{j_2})\Vert _{L^2L^1}\\&\lesssim \sum _{j_1= j_2+O(1),j_1>k}2^{(\sigma +1+d/2)(k-j_1)+(\sigma +2+d/2)j_1}\Vert h_{j_1}\Vert _{L^2L^2}\Vert \psi _{j_2}\Vert _{L^{\infty }L^2}\\&\lesssim \min \{\tilde{s}_k\Vert \psi \Vert _{l^2X^{s}},\tilde{p}_k\Vert h\Vert _{Z^{1,s+2}}\}, \end{aligned}$$

and when $\sigma +d/2+1\le \delta$ we have

$$\begin{aligned}&\sum _{j_1= j_2+O(1),j_1>k}\Vert S_k \nabla ( h_{j_1}\nabla \psi _{j_2})\Vert _{l^2N^{\sigma }}\\&\lesssim \sum _{j_1= j_2+O(1),j_1>k}2^{(\sigma +1+d/2-2\delta )k+(2\delta +1) j_1}\Vert h_{j_1}\Vert _{L^2L^2}\Vert \psi _{j_2}\Vert _{L^{\infty }L^2}\\&\lesssim \min \{\tilde{s}_k\Vert \psi \Vert _{l^2X^{s}},\tilde{p}_k\Vert h\Vert _{Z^{1,s+2}}\}, \end{aligned}$$

Next, we prove the bounds (5.4)–(5.6) and (5.9)–(5.11). These bounds can be estimated similarly, we only prove (5.4) and (5.9) in detail. Indeed, by duality we have

$$\begin{aligned} \Vert S_k(B\psi )\Vert _{l^2N^{\sigma }}\lesssim 2^{\sigma k}\Vert S_k(B\psi )\Vert _{L^2L^2}. \end{aligned}$$

Then using Littlewood–Paley dichotomy to divide this into low-high, high-low and high-high cases. For the low-high case, by Sobolev embedding we have

$$\begin{aligned} 2^{\sigma k}\Vert S_k(B_{<k}\psi _k)\Vert _{L^2L^2}&\lesssim \Vert B_{<k}\Vert _{L^{\infty }L^{\infty }}2^{\sigma k}\Vert \psi _k\Vert _{L^2L^2}\lesssim \tilde{p}_k\Vert B\Vert _{Z^{1,s}} . \end{aligned}$$

If $-s\le \sigma \le s-\delta$ we also have

$$\begin{aligned} 2^{\sigma k}\Vert S_k(B_{<k}\psi _k)\Vert _{L^2L^2}&\lesssim \mathbf{1 }_{[-s,\frac{d}{2})}(\sigma )\sum _{0\le l<k}2^{(d/2+2\delta -\sigma )(l-k)} \Vert \nabla B_l\Vert _{L^{\infty }H^{\sigma -1}}2^{(d/2+2\delta ) k}\Vert \psi _k\Vert _{L^2L^2}\\&\quad +\mathbf{1 }_{[\frac{d}{2},s-\delta ]}(\sigma )\sum _{0\le l<k}\Vert \nabla B_l\Vert _{L^{\infty }H^{\sigma -1}}2^{\sigma k}\Vert \psi _k\Vert _{L^2L^2}\\&\lesssim \mathbf{1 }_{[-s,\frac{d}{2})}(\sigma )\sum _{0\le l<k}2^{(d/2+2\delta -\sigma )(l-k)} 2^{\delta (k-l)}\tilde{s}_k 2^{(d/2+2\delta ) k}\Vert \psi _k\Vert _{L^2L^2}\\&\quad +\mathbf{1 }_{[\frac{d}{2},s-\delta ]}(\sigma )\sum _{0\le l<k}2^{\delta (k-l)}\tilde{s}_k2^{\sigma k}\Vert \psi _k\Vert _{L^2L^2}\\&\lesssim \tilde{s}_k \Vert \psi \Vert _{l^2X^{s}}. \end{aligned}$$

The high-low case can be estimated similarly. For the high-high case, by Sobolev embedding when $\sigma +d/2\ge 0$ we have

$$\begin{aligned} 2^{\sigma k}\Vert S_k(B_l\psi _l)\Vert _{L^2L^2}&\lesssim \sum _{l>k} 2^{(\sigma +d/2+\delta )(k-l)} 2^{(\sigma +d/2+\delta )l} \Vert B_l\Vert _{L^{\infty }L^2}\Vert \psi _l\Vert _{L^2L^2}\\&\lesssim \min \{\tilde{s}_k\Vert \psi \Vert _{l^2 X^{s}},\tilde{p}_k\Vert B\Vert _{Z^{1,s}} \}, \end{aligned}$$

and when $\sigma +d/2<0$ we have

$$\begin{aligned} 2^{\sigma k}\Vert S_k(B_l\psi _l)\Vert _{L^2L^2}&\lesssim \sum _{l>k} 2^{(\sigma +d/2)k} \Vert B_l\Vert _{L^{\infty }L^2}\Vert \psi _l\Vert _{L^2L^2}\\&\lesssim \min \{\tilde{s}_k\Vert \psi \Vert _{l^2 X^{s}},\tilde{p}_k\Vert B\Vert _{Z^{1,s}} \}, \end{aligned}$$

These imply the bound (5.4) and (5.9).

Finally, we prove the bound (5.12). If $\sigma >d/2-1+\delta$, by duality and Sobolev embedding, we have

$$\begin{aligned} 2^{\sigma k}\Vert A_{<k}\nabla \psi _k\Vert _{L^2L^2}&\lesssim \sum _{l\le k} 2^{(d/2-1)l} \Vert \nabla A_{l}\Vert _{L^\infty L^2} 2^{(\sigma +1) k}\Vert \psi _k\Vert _{L^2L^2}\lesssim p_k\Vert A\Vert _{Z^{1,\sigma +1}}. \end{aligned}$$

If $\sigma \le d/2-1+\delta$, we have

$$\begin{aligned} 2^{\sigma k}\Vert A_{<k}\nabla \psi _k\Vert _{L^2L^2}&\lesssim \sum _{0\le l<k} 2^{(d/2-1-\sigma +\delta )(l-k)}\Vert \nabla A_l\Vert _{L^{\infty }H^\sigma } 2^{(d/2+\delta )k} \Vert \psi _k\Vert _{L^2L^2}\\&\lesssim p_k\Vert A\Vert _{Z^{1,\sigma +1}}. \end{aligned}$$

Then the bound (5.12) follows. Hence this completes the proof of the lemma. $\square$

We shall also require the following bounds on commutators.

### Proposition 5.3

(Commutator bounds). Let $s>\frac{d}{2},d\ge 2$. Let *m*(*D*) be a multiplier with symbol $m\in S^0$. Assume $h\in {\mathbf {Y}}^{s+2}$, $A\in Z^{1,s+1}$ and $\psi _k \in l^2X^s$, frequency localized at frequency $2^k$. If $-s\le \sigma \le s$ we have

5.13 $$\begin{aligned}&\Vert \nabla [S_{<k-4}h,m(D)]\nabla \psi _k\Vert _{l^2N^{\sigma }}\lesssim \min \{ \Vert h\Vert _{{\mathbf {Y}}^{\sigma +2}}\Vert \psi _k\Vert _{l^2X^s},\Vert h\Vert _{{\mathbf {Y}}^{s+2}}\Vert \psi _k\Vert _{l^2X^{\sigma }}\}, \nonumber \\ \end{aligned}$$

5.14 $$\begin{aligned}&\Vert [S_k,A_{<k-4}]\nabla \psi _k\Vert _{l^2N^{\sigma }}\lesssim \min \{\Vert A\Vert _{Z^{1,s+1}}\Vert \psi _k\Vert _{l^2X^{\sigma }},\Vert A\Vert _{Z^{1,\sigma +1}}\Vert \psi _k\Vert _{l^2X^{s}}\}.\nonumber \\ \end{aligned}$$

### Proof

First we estimate (5.13). In [21, Proposition 3.2], it was shown that

$$\begin{aligned} \nabla [S_{<k-4}g,m(D)]\nabla S_k \psi =L(\nabla S_{<k-4}g,\nabla S_k \psi ), \end{aligned}$$

where *L* is a translation invariant operator satisfying

$$\begin{aligned} L(f,g)(x)=\int f(x+y)g(x+z)\tilde{m}(y+z)dydz,\ \ \ \tilde{m}\in L^1. \end{aligned}$$

Given this representation, as we are working in translation-invariant spaces, by (5.1) the bound (5.13) follows.

Next, for the bound (5.14). Since

$$\begin{aligned}{}[S_k,A_{<k}]\nabla \psi =\int _0^1 \int 2^{kd}{\check{\varphi }}(2^k y)2^k y \nabla A_{<k}(x-sy) 2^{-k}\nabla \psi _{[k-3,k+3]}(x-y)dyds, \end{aligned}$$

By translation-invariance and the similar argument to (5.9), the bound (5.14) follows. This completes the proof of the lemma. $\square$

## Local Energy Decay and the Linearized Problem

In this section, we consider a linear Schrödinger equation

6.1 $$\begin{aligned} \left\{ \begin{aligned}&i\partial _t \psi +\partial _{\alpha }g^{\alpha {\beta }}\partial _{{\beta }}\psi +2i A^{\alpha }\partial _{\alpha }\psi =F,\\&\psi (0)=\psi _0, \end{aligned}\right. \end{aligned}$$

and, under suitable assumptions on the coefficients, we prove that the solution satisfies suitable energy and local energy bounds.

### The linear paradifferential Schrödinger flow

As an intermediate step, here we prove energy and local energy bounds for a frequency localized linear paradifferential Schrödinger equation

6.2 $$\begin{aligned} i\partial _t\psi _k+\partial _{\alpha }(g^{\alpha {\beta }}_{<k-4}\partial _{{\beta }}\psi _k)+2iA^{\alpha }_{<k-4}\partial _{\alpha }\psi _k=f_k. \end{aligned}$$

We begin with the energy estimates, which are fairly standard:

#### Lemma 6.1

(Energy-type estimate). Let $d\ge 2$, $\psi _k$ solves the equation (6.2) with initial data $\psi _k(0)$ in the time interval [0, 1]. For a fixed $s>\frac{d}{2}$, assume that $A\in Z^{1,s+1}$, $\psi _k\in l^2_kX_k$, $f_{1k}\in N$ and $f_{2k}\in L^1L^2$, where $f_k=f_{1k}+f_{2k}$. Then we have

6.3 $$\begin{aligned} \begin{aligned} \Vert \psi _k\Vert _{L^{\infty }_tL^2_x}^2 \lesssim&\Vert \psi _k(0)\Vert _{L^2}^2+\Vert A\Vert _{Z^{1,s+1}}\Vert \psi _k\Vert _{X_k}^2 +\Vert \psi _k\Vert _{X_k}\Vert f_{1k}\Vert _{N_k}\\&+\Vert \psi _k\Vert _{L^{\infty }L^2}\Vert f_{2k}\Vert _{L^1L^2}. \end{aligned} \end{aligned}$$

#### Proof

By (6.2), we have

$$\begin{aligned} \frac{1}{2}\frac{d}{dt}\Vert \psi _k\Vert _{L^2}^2&= \mathop {\mathrm{Re}}\nolimits \langle \psi _k,\partial _t \psi _k\rangle \\&= \mathop {\mathrm{Re}}\nolimits \langle \psi _k,i\partial _{\alpha }g^{\alpha {\beta }}_{<k-4}\partial _{{\beta }}\psi _k-2A^{\alpha }_{<k-4}\partial _{\alpha }\psi _k-if_k\rangle \\&= -\mathop {\mathrm{Re}}\nolimits \langle \partial _{\alpha }\psi _k,ig^{\alpha {\beta }}_{<k-4}\partial _{{\beta }}\psi _k\rangle -\mathop {\mathrm{Re}}\nolimits \int _{{{\mathbb {R}}}^d} A^{\alpha }_{<k-4}\partial _{\alpha }|\psi _k|^2 dx-\mathop {\mathrm{Re}}\nolimits \langle \psi _k,if_k\rangle \\&= \mathop {\mathrm{Re}}\nolimits \int _{{{\mathbb {R}}}^d} \partial _{\alpha }A^{\alpha }_{<k-4}|\psi _k|^2 dx-\mathop {\mathrm{Re}}\nolimits \langle \psi _k,if_k\rangle , \end{aligned}$$

and notice that for each $t\in [0,1]$ we have by duality and Sobolev embedding

$$\begin{aligned} \Vert \psi _k(t)\Vert _{L^2}^2&\lesssim \Vert \psi _k(0)\Vert _{L^2}^2+\int _0^1\int _{{{\mathbb {R}}}^d} |\partial _{\alpha }A^{\alpha }_{<k-4}||\psi _k|^2 dxdt +\Vert \psi _k\Vert _{X_k}\Vert f_{1k}\Vert _{N_k}\\&\quad +\Vert \psi _k\Vert _{L^{\infty }L^2}\Vert f_{2k}\Vert _{L^1L^2}\\&\lesssim \Vert \psi _k(0)\Vert _{L^2}^2+\Vert A\Vert _{Z^{1,s+1}}\Vert \psi _k\Vert _{X_k}^2\\&\quad +\Vert \psi _k\Vert _{X_k}\Vert f_{1k}\Vert _{N_k}+\Vert \psi _k\Vert _{L^{\infty }L^2}\Vert f_{2k}\Vert _{L^1L^2} \end{aligned}$$

We take the supremum over *t* on the left hand side and the conclusion follows. $\square$

Next, we prove the main result of this section, namely the local energy estimates for solutions to (6.2):

#### Proposition 6.2

(Local energy decay). Let $d\ge 3$, assume that the coefficients $g^{\alpha {\beta }}=\delta ^{\alpha {\beta }}+h^{\alpha {\beta }}$ and $A^{\alpha }$ in (6.2) satisfy

6.4 $$\begin{aligned} \Vert h\Vert _{{\mathbf {Y}}^{s+2}},\ \Vert A\Vert _{Z^{1,s+1}}\ll 1 \end{aligned}$$

for some $s>\frac{d}{2}$. Let $\psi _k$ be a solution to (6.2) which is localized at frequency $2^k$. Then the following estimate holds:

6.5 $$\begin{aligned} \Vert \psi _k\Vert _{l^2_k X_k}\lesssim \Vert \psi _{0k}\Vert _{L^2}+\Vert f_k\Vert _{l^2_kN_k} \end{aligned}$$

#### Proof

The proof is closely related to that given in [21, 22]. However, here we are able to relax the assumptions both on the metric *g* and on the magnetic potential *A*. In the latter case, unlike in [21, 22], we treat the magnetic term $2i A^{\alpha }_{<k-4}\partial _{\alpha }\psi _k$ as a part of the linear equation, which allows us to avoid bilinear estimates for this term and use only the bound for *A* in ${\mathbf {Z}}^{1,s+1}$.

As an intermediate step in the proof, we will establish a local energy decay bound in a cube $Q\in {{\mathcal {Q}}}_l$ with $0\le l\le k$:

6.6 $$\begin{aligned} \begin{aligned} 2^{k-l}\Vert \psi _k\Vert _{L^2L^2([0,1]\times Q)}^2&\lesssim \ \Vert \psi _k\Vert _{L^{\infty }L^2}^2+ \Vert f_k\Vert _{N_k} \Vert \psi _k \Vert _{X_k} \\&\quad +(2^{-k}+\Vert A\Vert _{Z^{1,s+1}}+\Vert h\Vert _{{\mathbf {Y}}^{s+2}})\Vert \psi _k\Vert _{l^2_kX_k}^2. \end{aligned} \end{aligned}$$

The proof of this bound is based on a positive commutator argument using a well chosen multiplier ${{\mathcal {M}}}$. This will be first-order differential operator with smooth coefficients which are localized at frequency $\lesssim 1$. Precisely, we will use a multiplier ${{\mathcal {M}}}$ which is a sef-adjoint differential operator having the form

6.7 $$\begin{aligned} i2^k{{\mathcal {M}}}=a^{\alpha }(x)\partial _{\alpha }+\partial _{\alpha }a^{\alpha }(x) \end{aligned}$$

with uniform bounds on *a* and its derivatives.

Before proving (6.5), we need the following lemma which is used to dismiss the $(g-I_d)$ contribution to the commutator $[\partial _{\alpha }g^{\alpha {\beta }}\partial _{{\beta }},{{\mathcal {M}}}]$.

#### Lemma 6.3

Let $s>\frac{d}{2}$ and $d\ge 3$, assume that $h\in {\mathbf {Y}}^{s+2}$, $A\in Z^{1,s+1}$ and $\psi \in l^2_kX_k$, let ${{\mathcal {M}}}$ be as (6.7). Then we have

6.8 $$\begin{aligned}&\int _0^1\langle [\partial _{\alpha }h^{\alpha {\beta }}_{\le k}\partial _{{\beta }},{{\mathcal {M}}}]\psi _k,\psi _k\rangle ds\lesssim \Vert h\Vert _{{\mathbf {Y}}^{s+2}}\Vert \psi _k\Vert _{l^2_kX_k}^2, \end{aligned}$$

6.9 $$\begin{aligned}&\int _0^1\mathop {\mathrm{Re}}\nolimits \langle A^{\alpha }_{<k-4}\partial _{\alpha }\psi _k,{{\mathcal {M}}}\psi _k \rangle ds\lesssim \Vert A\Vert _{Z^{1,s+1}}\Vert \psi _k\Vert _{X_k}^2. \end{aligned}$$

#### Proof of Lemma 6.3

By (6.7) and directly computations, we get

$$\begin{aligned}{}[\partial _{\alpha }h^{\alpha {\beta }}\partial _{{\beta }},{{\mathcal {M}}}]\approx 2^{-k}[\nabla (h\nabla a+a\nabla h)\nabla +\nabla h\nabla ^2 a+h\nabla ^3 a]. \end{aligned}$$

Then it suffices to estimate

$$\begin{aligned} 2^{-k}\int _0^1 \langle (h_{\le k}\nabla a+a\nabla h_{\le k})\nabla \psi _k,\nabla \psi _k\rangle dt+2^{-k}\int _0^1\langle (\nabla h_{\le k}\nabla ^2 a+h_{\le k}\nabla ^3 a)\psi _k,\psi _k\rangle dt \end{aligned}$$

The first integral is estimated by (5.1) and (5.2). Using Sobolev embedding, the second integral is bounded by

$$\begin{aligned} 2^{-k}\int _0^1 \langle (\nabla h_{\le k}+h_{\le k})\psi _k,\psi _k\rangle dt \lesssim \Vert \langle \nabla \rangle h_{\le k}\Vert _{L^{\infty }}2^{-k}\Vert \psi _k\Vert _{L^2L^2}^2 \lesssim \Vert \nabla h\Vert _{L^{\infty }H^s}\Vert \psi _k\Vert _{l^2_kX_k}^2. \end{aligned}$$

Hence, the bound (6.8) follows.

For the second bound (6.9), by (6.7) and integration by parts we rewrite the following term as

$$\begin{aligned}&\mathop {\mathrm{Re}}\nolimits \langle A^{\alpha }\partial _{\alpha }\psi ,i\sum _{{\beta }=1}^d(a_{{\beta }}\partial _{{\beta }}+\partial _{{\beta }}a_{{\beta }})\psi \rangle \\&= \mathop {\mathrm{Re}}\nolimits \sum _{{\beta }=1}^d\int _{{{\mathbb {R}}}^d} \Big [ i\partial _{\alpha } ({\bar{\psi }}A^{\alpha }a_{{\beta }}\partial _{{\beta }}\psi )-i{\bar{\psi }}\partial _{\alpha }A^{\alpha }a_{{\beta }}\partial _{{\beta }}\psi -i{\bar{\psi }}A^{\alpha }\partial _{\alpha }a_{{\beta }} \partial _{{\beta }}\psi -i{\bar{\psi }}A^{\alpha }a_{{\beta }}\partial _{\alpha {\beta }}^2\psi \\&\quad +i\partial _{{\beta }}(A^{\alpha }\partial _{\alpha }{\bar{\psi }}a_{{\beta }}\psi )-i\partial _{{\beta }}A^{\alpha }\partial _{\alpha }{\bar{\psi }}a_{{\beta }}\psi -iA^{\alpha }\partial _{\alpha {\beta }}^2{\bar{\psi }}a_{{\beta }}\psi \Big ] dx\\&\approx \int _{{{\mathbb {R}}}^d} \langle \nabla \rangle A\psi \nabla \psi dx. \end{aligned}$$

Then we bound the left-hand side of (6.9) by

$$\begin{aligned} \int _0^1\mathop {\mathrm{Re}}\nolimits \langle A^{\alpha }_{<k-4}\partial _{\alpha }\psi _k,{{\mathcal {M}}}\psi _k \rangle ds&\lesssim 2^{-k} \int _0^1\int _{{{\mathbb {R}}}^d} |\langle \nabla \rangle A_{<k} \psi _k \nabla \psi _k |dxds\\&\lesssim \Vert \nabla A\Vert _{L^{\infty }H^s}\Vert \psi _k\Vert _{L^2L^2}^2. \end{aligned}$$

This implies the bound (6.9), and hence completes the proof of the lemma. $\square$

Returning to the proof of (6.6), for the self-adjoint multiplier ${{\mathcal {M}}}$ we compute

$$\begin{aligned} \frac{d}{dt}\langle \psi _k,{{\mathcal {M}}}\psi _k\rangle&= 2\mathop {\mathrm{Re}}\nolimits \langle \partial _t\psi _k,{{\mathcal {M}}}\psi _k\rangle \\&= 2\mathop {\mathrm{Re}}\nolimits \langle i\partial _{\alpha }(g^{\alpha {\beta }}_{<k-4}\partial _{{\beta }}\psi _k)-2A^{\alpha }_{<k-4}\partial _{\alpha }\psi _k-if_k,{{\mathcal {M}}}\psi _k \rangle \\&= i\langle [-\partial _{\alpha }g^{\alpha {\beta }}_{<k-4}\partial _{{\beta }},{{\mathcal {M}}}]\psi _k,\psi _k\rangle +2\mathop {\mathrm{Re}}\nolimits \langle -2A^{\alpha }_{<k-4}\partial _{\alpha }\psi _k-if_k,{{\mathcal {M}}}\psi _k \rangle \end{aligned}$$

We then use the multiplier ${{\mathcal {M}}}$ as in [21, 22] so that the following three properties hold:

1. Boundedness on frequency $2^k$ localized functions,
  $$\begin{aligned} \Vert {{\mathcal {M}}}u\Vert _{L^2_x}\lesssim \Vert u\Vert _{L^2_x}. \end{aligned}$$
2. Boundedness in *X*,
  $$\begin{aligned} \Vert {{\mathcal {M}}}u\Vert _{X}\lesssim \Vert u\Vert _{X}. \end{aligned}$$
3. Positive commutator,
  $$\begin{aligned} i\langle [-\partial _{\alpha }g^{\alpha {\beta }}_{<k-4}\partial _{{\beta }},{{\mathcal {M}}}]u,u\rangle \gtrsim 2^{k-l}\Vert u\Vert ^2_{L^2_{t,x}([0,1]\times Q)}-O(2^{-k}+\Vert h\Vert _{{\mathbf {Y}}^{s+2}})\Vert u\Vert _{l^2_kX_k}^2. \end{aligned}$$

If these three properties hold for $u=\psi _k$, then by (6.9) and (6.4) the bound (6.6) follows.

We first do this when the Fourier transform of the solution $\psi _k$ is restricted to a small angle

6.10 $$\begin{aligned} \text {supp} \widehat{\psi }_k\subset \{|\xi |\lesssim \xi _1\}. \end{aligned}$$

Without loss of generality due to translation invariance, $Q=\{|x_j|\le 2^l:j=1,\ldots ,d\}$, and we set *m* to be a smooth, bounded, increasing function such that $m'(s)=\varphi ^2(s)$ where $\varphi$ is a Schwartz function localized at frequencies $\lesssim 1$, and $\varphi \approx 1$ for $|s|\le 1$. We rescale *m* and set $m_l(s)=m(2^{-l}s)$. Then, we fix

$$\begin{aligned} {{\mathcal {M}}}=\frac{1}{i2^k} (m_l(x_1)\partial _1+\partial _1 m_l(x_1)). \end{aligned}$$

The properties (1) and (2) are immediate due to the frequency localization of $u=\psi _k$ and $m_l$ as well as the boundedness of $m_l$. By (6.8) it suffices to consider the property (3) for the operator

$$\begin{aligned} -\Delta =-\partial _{\alpha }g^{\alpha {\beta }}_{<k-4}\partial _{{\beta }}+\partial _{\alpha }h^{\alpha {\beta }}_{<k-4}\partial _{{\beta }}. \end{aligned}$$

This yields

$$\begin{aligned} i2^k[-\Delta ,{{\mathcal {M}}}]=-2^{-l+2}\partial _1 \varphi ^2(2^{-l}x_1)\partial _1+O(1), \end{aligned}$$

and hence

$$\begin{aligned} i2^k\langle [-\Delta ,{{\mathcal {M}}}]\psi _k,\psi _k\rangle =2^{-l+2}\Vert \varphi (2^{-l}x_1)\partial _1\psi _k\Vert _{L^2L^2}^2+O(\Vert \psi _k\Vert _{L^2L^2}^2) \end{aligned}$$

Utilizing our assumption (6.10), it follows that

$$\begin{aligned} 2^{k-l}\Vert \varphi (2^{-l}x_1)\psi _k\Vert _{L^2L^2}^2\lesssim i\langle [-\Delta ,{{\mathcal {M}}}]\psi _k,\psi _k\rangle +2^{-k}O(\Vert \psi _k\Vert _{L^2L^2}^2) \end{aligned}$$

which yields (3) when combined with (6.8).

We proceed to reduce the problem to the case when (6.10) holds. We let $\{ \theta _j (\omega ) \}_{j=1}^d$ be a partition of unity,

$$\begin{aligned} \sum _{j}\theta _j(\omega )=1,\ \ \ \ \omega \in {{\mathbb {S}}}^{d-1}, \end{aligned}$$

where $\theta _j(\omega )$ is supported in a small angle about the *j*-th coordinate axis. Then, we can set $\psi _{k,j}=\Theta _{k,j}\psi _k$ where

$$\begin{aligned} {{\mathcal {F}}}\Theta _{k,j}\psi =\theta _j(\frac{\xi }{|\xi |})\sum _{k-1\le l\le k+1}\varphi _l(\xi )\widehat{\psi }(t,\xi ). \end{aligned}$$

We see that

$$\begin{aligned}&(i\partial _t+\partial _{\alpha }g^{\alpha {\beta }}_{<k-4}\partial _{{\beta }})\psi _{k,j}+2iA^{\alpha }_{<k-4}\partial _{\alpha }\psi _{k,j}\\&= \Theta _{k,j}f_k-\partial _{\alpha }[\Theta _{k,j},g^{\alpha {\beta }}_{<k-4}]\partial _{{\beta }}\psi _k-2i[\Theta _{k,j},A^{\alpha }_{\le k-4}]\partial _{\alpha }\psi _k. \end{aligned}$$

By applying ${{\mathcal {M}}}$, suitably adapted to the correct coordinate axis, to $\psi _{k,j}$ and summing over *j*, we obtain

$$\begin{aligned}&2^{k-l}\Vert \psi _k\Vert _{L^2L^2([0,1]\times Q)}^2\\&\lesssim \ \Vert \psi _k\Vert _{L^{\infty }L^2}^2+\sum _{j=1}^d\int _0^1\langle -\Theta _{k,j}f_k,{{\mathcal {M}}}\psi _{k,j}\rangle ds\\&\quad +\sum _{j=1}^d\int \langle [\Theta _{k,j},\partial _{\alpha }g^{\alpha {\beta }}_{<k-4}\partial _{{\beta }}]\psi _k+[\Theta _{k,j},2iA^{\alpha }_{<k-4}]\partial _{\alpha }\psi _k,{{\mathcal {M}}}\psi _{k,j}\rangle ds\\&\quad +(2^{-k}+\Vert A\Vert _{Z^{1,s+1}}+\Vert h\Vert _{{\mathbf {Y}}^{s+2}})\Vert \psi _k\Vert _{l^2_kX_k}^2\\&\lesssim \ \Vert \psi _k\Vert _{L^{\infty }L^2}^2+\Vert f_k\Vert _{N_k}\Vert \psi _k\Vert _{X_k} +(2^{-k}+\Vert A\Vert _{Z^{1,s+1}}+\Vert h\Vert _{{\mathbf {Y}}^{s+2}})\Vert \psi _k\Vert _{l^2_kX_k}^2. \end{aligned}$$

The commutator is done via (5.13) and (5.14). Then (6.6) follows.

Next we use the bound (6.6) to complete the proof of Proposition 6.2. Taking the supremum in (6.6) over $Q\in {{\mathcal {Q}}}_l$ and over *l*, we obtain

$$\begin{aligned} \begin{aligned} 2^k\Vert \psi _k\Vert _{X}^2&\lesssim \Vert \psi _k\Vert _{L^{\infty }L^2}^2+\Vert f_{1k}\Vert _{N_k}\Vert \psi _k\Vert _{X_k}+\Vert f_{2k}\Vert _{L^1L^2}\Vert \psi _k\Vert _{L^{\infty }L^2}\\&\quad +(2^{-k}+\Vert A\Vert _{Z^{1,s+1}}+\Vert h\Vert _{{\mathbf {Y}}^{s+2}})\Vert \psi _k\Vert _{l^2_kX_k}^2\\&\lesssim \Vert \psi _k\Vert _{L^{\infty }L^2}^2+\Vert f_{1k}\Vert _{N_k}\Vert \psi _k\Vert _{X_k}+\Vert f_{2k}\Vert _{L^1L^2}^2\\&\quad +(2^{-k}+\Vert A\Vert _{Z^{1,s+1}}+\Vert h\Vert _{{\mathbf {Y}}^{s+2}})\Vert \psi _k\Vert _{l^2_kX_k}^2. \end{aligned} \end{aligned}$$

Combined with (6.3), we get

6.11 $$\begin{aligned} \begin{aligned} \Vert \psi _k\Vert _{X_k}^2&\lesssim \Vert \psi _k(0)\Vert _{L^2}^2 +\Vert f_{1k}\Vert _{N_k}^2+\Vert f_{2k}\Vert _{L^1L^2}^2\\&\quad +(2^{-k}+\Vert A\Vert _{Z^{1,s+1}}+\Vert h\Vert _{{\mathbf {Y}}^{s+2}})\Vert \psi _k\Vert _{l^2_kX_k}^2. \end{aligned} \end{aligned}$$

We now finish the proof by incorporating the summation over cubes. We let $\{\chi _Q\}$ denote a partition via functions which are localized to frequencies $\lesssim 1$ which are associated to cubes *Q* of scale $M2^k$. We also assume that $|\nabla ^l\chi _Q|\lesssim (2^k M)^{-l}$, $l=1,2$. Thus,

$$\begin{aligned}&(i\partial _t+\partial _{\alpha }g^{\alpha {\beta }}_{<k-4}\partial _{{\beta }})\chi _Q \psi _k+2iA^{\alpha }_{<k-4}\partial _{\alpha }\chi _Q \psi _k\\&= \chi _Q f_k+[\partial _{\alpha }g^{\alpha {\beta }}_{<k-4}\partial _{{\beta }},\chi _Q]\psi _k+2iA^{\alpha }_{<k-4}\partial _{\alpha }\chi _Q\cdot \psi _k \end{aligned}$$

Applying (6.3) to $\chi _Q\psi _k$, we obtain

$$\begin{aligned}&\sum _Q \Vert \chi _Q\psi _k\Vert _{L^{\infty }L^2}^2\\&\lesssim \sum _Q \Vert \chi _Q\psi _k(0)\Vert _{L^2}^2 +\Vert A\Vert _{Z^{1,s+1}}\sum _Q\Vert \chi _Q\psi _k\Vert _{X_k}^2\\&\quad +(\sum _Q\Vert \chi _Qf_k\Vert _{N_k}^2)^{1/2}(\sum _Q\Vert \chi _Q\psi _k\Vert _{X_k}^2)^{1/2}\\&\quad +\sum _Q\Vert [\partial _{\alpha }g^{\alpha {\beta }}_{<k-4}\partial _{{\beta }},\chi _Q]\psi _k+2iA^{\alpha }_{<k-4}\partial _{\alpha }\chi _Q\cdot \psi _k\Vert _{L^1L^2}^2. \end{aligned}$$

But by (6.4) we have

6.12 $$\begin{aligned} \begin{aligned} \sum _Q\Vert [\nabla g\nabla ,\chi _Q]\psi _k \Vert _{L^1L^2}^2&\lesssim \sum _Q\Vert \nabla g\cdot \nabla \chi _Q\cdot \psi _k+g\nabla (\nabla \chi _Q\cdot \psi _k)\Vert _{L^1L^2}^2\\&\lesssim (1+\Vert h\Vert _{Z^{1,s+2}}) M^{-2}\sum _Q \Vert \chi _Q\psi _k\Vert _{L^{\infty }L^2}^2, \end{aligned} \end{aligned}$$

and also

6.13 $$\begin{aligned} \sum _Q\Vert 2iA^{\alpha }_{<k-4}\partial _{\alpha }\chi _Q\cdot \psi _k\Vert _{L^1L^2}^2\lesssim (1+\Vert A\Vert _{Z^{1,s}}) M^{-2}\sum _Q \Vert \chi _Q\psi _k\Vert _{L^{\infty }L^2}^2. \end{aligned}$$

For *M* sufficiently large, we can bootstrap the commutator terms, and, after a straightforward transition to cubes of scale $2^k$ rather than $M2^k$, we observe that

6.14 $$\begin{aligned} \begin{aligned} \Vert \psi _k\Vert _{l^2_kL^{\infty }L^2}^2&\lesssim \Vert \psi _k(0)\Vert _{L^2}^2 +\Vert A\Vert _{Z^{1,s+1}}\Vert \psi _k\Vert _{l_k^2X_k}^2 +\Vert f_k\Vert _{l^2_kN_k}\Vert \psi _k\Vert _{l^2_kX_k}. \end{aligned} \end{aligned}$$

We now apply (6.11) to $\chi _Q\psi _k$, and then by (6.12) and (6.13) we see that

$$\begin{aligned} \sum _Q \Vert \chi _Q \psi _k\Vert _{X_k}^2 \lesssim&\Vert \psi _k(0)\Vert _{L^2}^2+\sum _Q\Vert \chi _Q f_k\Vert _{N_k}^2+M^{-2}\sum _Q\Vert \chi _Q\psi _k\Vert _{X_k}^2\\&+(2^{-k}+\Vert h\Vert _{{\mathbf {Y}}^{s+2}}+\Vert A\Vert _{Z^{1,s+1}})\sum _Q\Vert \chi _Q\psi _k\Vert _{l^2_kX_k}^2. \end{aligned}$$

For $M\gg 1$, we have

$$\begin{aligned} M^{-d}\Vert \psi _k\Vert _{l^2_kX_k}^2&\lesssim \Vert \psi _k(0)\Vert _{L^2}^2+\Vert f_k\Vert _{l^2_kN_k}^2 +(2^{-k}+\Vert h\Vert _{{\mathbf {Y}}^{s+2}}+\Vert A\Vert _{Z^{1,s+1}})\Vert \psi _k\Vert _{l^2_kX_k}^2. \end{aligned}$$

By (6.4), for *k* sufficiently large (depending on *M*), we may absorb the last terms in the right-hand side into the left, i.e

$$\begin{aligned} \Vert \psi _k\Vert _{l^2_kX_k}^2\lesssim \Vert \psi _k(0)\Vert _{L^2}^2+\Vert f_k\Vert _{l^2_kN_k}^2. \end{aligned}$$

On the other hand, for the remaining bounded range of *k*, we have

$$\begin{aligned} \Vert \psi \Vert _{X_k}\lesssim \Vert \psi \Vert _{L^{\infty }L^2}, \end{aligned}$$

and then (6.14) and (6.4) gives

$$\begin{aligned} \Vert \psi _k\Vert _{l^2_kX_k}^2&\lesssim \Vert \psi _k(0)\Vert _{L^2}^2 +\Vert A\Vert _{Z^{1,s+1}}\Vert \psi _k\Vert _{l_k^2X_k}^2 +\Vert f_k\Vert _{l^2_kN_k}\Vert \psi _k\Vert _{l^2_kX_k}\\&\lesssim \Vert \psi _k(0)\Vert _{L^2}^2 +\Vert f_k\Vert _{l^2_kN_k}^2, \end{aligned}$$

which finishes the proof of (6.5). $\square$

### The full linear problem

Here we use the bounds for the paradifferential equation in the previous subsection in order to prove similar bounds for the full equation (6.1):

#### Proposition 6.4

(Well-posedness). Let $s>\frac{d}{2}$, $d\ge 3$ and $h=g-I_d\in {\mathbf {Y}}^{s+2}$, assume that the metric *g*, and the magnetic potential *A* satisfy

$$\begin{aligned} \Vert h\Vert _{{\mathbf {Y}}^{s+2}},\ \Vert A\Vert _{Z^{1,s+1}}\ll 1. \end{aligned}$$

Then the equation (6.1) is well-posed for initial data $\psi _0\in H^{\sigma }$ with $-s\le \sigma \le s$, and we have the estimate

6.15 $$\begin{aligned} \Vert \psi \Vert _{l^2X^{\sigma }}\lesssim \Vert \psi _0\Vert _{H^{\sigma }}+\Vert F\Vert _{l^2N^{\sigma }}. \end{aligned}$$

Moreover, for $0\le \sigma \le s$ we have the estimate

6.16 $$\begin{aligned} \Vert \psi \Vert _{l^2{\mathbf {X}}^{\sigma }}\lesssim \Vert \psi _0\Vert _{H^{\sigma }}+\Vert F\Vert _{l^2N^{\sigma }\cap L^2H^{\sigma -2}}. \end{aligned}$$

#### Proof

The well-posedness follows in a standard fashion from a similar energy estimate for the adjoint equation. Since the adjoint equation has a similar form, with similar bounds on the coefficients, such an estimate follows directly from (6.15). Thus, we now focus on the proof of the bound (6.15). For $\psi$ solving (6.1), we see that $\psi _k$ solves

$$\begin{aligned} \left\{ \begin{aligned}&i\partial _t \psi _k+\partial _{\alpha }g^{\alpha {\beta }}_{<k-4}\partial _{{\beta }}\psi _k+2iA^{\alpha }_{<k-4}\partial _{\alpha }\psi _k=F_k+H_k,\\&\psi _k(0)=\psi _{0k}, \end{aligned}\right. \end{aligned}$$

where

$$\begin{aligned} H_k =&-S_k\partial _{\alpha }g^{\alpha {\beta }}_{\ge k-4}\partial _{{\beta }}\psi -[S_k,\partial _{\alpha }g^{\alpha {\beta }}_{< k-4}\partial _{{\beta }}]\psi -2i[S_k,A^{\alpha }_{<k-4}]\partial _{\alpha }\psi \\&-2iS_k[ A^{\alpha }_{\ge k-4}\partial _{\alpha }\psi _k]. \end{aligned}$$

If we apply Proposition 6.2 to each of these equations, we see that

$$\begin{aligned} \Vert \psi _k\Vert _{l^2X^{\sigma }}^2\lesssim \Vert \psi _{0k}\Vert _{H^{\sigma }}^2+\Vert F_k\Vert _{l^2N^{\sigma }}^2+\Vert H_k\Vert _{l^2N^{\sigma }}^2. \end{aligned}$$

We claim that

$$\begin{aligned} \sum _{k}\Vert H_k\Vert _{l^2N^{\sigma }}^2\lesssim (\Vert h\Vert _{{\mathbf {Y}}^{s+2}}+\Vert A\Vert _{Z^{1,s+1}})^2\Vert \psi \Vert _{l^2X^{\sigma }}^2,\ \text {for }-s\le \sigma \le s. \end{aligned}$$

Indeed, the bound for the terms in $H_k$ follows from (5.7), (5.13), (5.14), (5.8), respectively. Then by the above two bounds, we obtain the estimate (6.15).

Finally, by the $\psi$-equation (6.1), for time derivative bound it suffices to consider the form

$$\begin{aligned} \partial _t\psi =\Delta \psi +\nabla (h\nabla \psi )+A\nabla \psi +F. \end{aligned}$$

Then by the standard Littlewood-Paley dichotomy and Bernstein’s inequality, for $0\le \sigma \le s$ we have the following estimates

$$\begin{aligned} \Vert \partial _t \psi \Vert _{L^2H^{\sigma -2}}\lesssim \Vert \psi \Vert _{L^{\infty }H^{\sigma }}+\Vert F\Vert _{L^2H^{\sigma -2}}, \end{aligned}$$

This, combined with (6.15), yields the bound (6.16), and then completes the proof of the Lemma. $\square$

### The linearized problem

Here we consider the linearized equation:

6.17 $$\begin{aligned} \left\{ \begin{aligned}&i\partial _t \Psi +\partial _{\alpha }g^{\alpha {\beta }}\partial _{{\beta }}\Psi +2iA^{\alpha }\partial _{\alpha }\Psi =F+G,\\&\Psi (0)=\Psi _0, \end{aligned}\right. \end{aligned}$$

where

$$\begin{aligned} G=-\nabla ({\mathcal {G}}\nabla \psi )-2i{\mathcal {A}}^{\alpha }\partial _{\alpha } \psi , \end{aligned}$$

and we prove the following.

#### Proposition 6.5

Let $s>\frac{d}{2}$, $0\le \sigma \le s-1$, $d\ge 3$ and $h=g-I_d\in {\mathbf {Y}}^{s+2}$, assume that $\Psi$ is a solution of (6.17), the metric *g* and *A* satisfy

$$\begin{aligned} \Vert h\Vert _{{\mathbf {Y}}^{s+2}},\ \Vert A\Vert _{Z^{1,s+1}}\ll 1. \end{aligned}$$

Then we have the estimate

6.18 $$\begin{aligned} \Vert \Psi \Vert _{l^2{\mathbf {X}}^{\sigma }}\lesssim \Vert \Psi _0\Vert _{H^{\sigma }}+\Vert F\Vert _{l^2N^{\sigma }\cap L^2H^{\sigma -2}}+(\Vert {\mathcal {G}}\Vert _{{\mathbf {Y}}^{\sigma +2}}+\Vert {\mathcal {A}}\Vert _{Z^{1,\sigma +1}})\Vert \psi \Vert _{l^2X^{s}}. \end{aligned}$$

#### Proof

For $\Psi$ solving (6.17), we see that $\Psi _k$ solves

$$\begin{aligned} \left\{ \begin{aligned}&i\partial _t \Psi _k+\partial _{\alpha }g^{\alpha {\beta }}_{<k-4}\partial _{{\beta }}\Psi _k+2iA^{\alpha }_{<k-4}\partial _{\alpha }\Psi _k=F_k+G_k+H_k,\\&\Psi _k(0)=\Psi _{0k}, \end{aligned}\right. \end{aligned}$$

where

$$\begin{aligned} G_k= & {} -S_k(\nabla ({\mathcal {G}}\nabla \psi )-2i{\mathcal {A}}^{\alpha }\partial _{\alpha } \psi ),\\ H_k= & {} -S_k\partial _{\alpha }g^{\alpha {\beta }}_{\ge k-4}\partial _{{\beta }}\Psi -[S_k,\partial _{\alpha }g^{\alpha {\beta }}_{< k-4}\partial _{{\beta }}]\Psi -2i[S_k,A^{\alpha }_{<k-4}]\partial _{\alpha }\Psi \\&-2iS_k[ A^{\alpha }_{\ge k-4}\partial _{\alpha }\Psi _k]. \end{aligned}$$

The proof of (6.18) is similar to that of (6.16). Here it suffices to prove

$$\begin{aligned} \sum _{k}\Vert G_k\Vert _{l^2N^{\sigma }}^2\lesssim & {} \Vert {\mathcal {G}}\Vert _{{\mathbf {Y}}^{\sigma +2}}^2\Vert \psi \Vert _{l^2X^{s}}^2+\Vert {\mathcal {A}}\Vert _{Z^{1,\sigma +1}}^2\Vert \psi \Vert _{l^2X^{s}}^2,\\ \Vert G\Vert _{L^2H^{\sigma -2}}\lesssim & {} (\Vert {{\mathcal {G}}}\Vert _{{\mathbf {Y}}^{\sigma +2}}+\Vert {{\mathcal {A}}}\Vert _{Z^{1,\sigma +1}})\Vert \psi \Vert _{l^2X^{s}}. \end{aligned}$$

Indeed, the bound for the terms in $G_k$ follows from (5.7), (5.3), (5.8) and (5.12). The second bound follows from a standard Littlewood-Paley decomposition and Bernstein’s inequality. This completes the proof of the Lemma. $\square$

## Well-Posedness in the Good Gauge

In this section we use the elliptic results in Sect. 4, the multilinear estimates in Sect. 5 and the linear local energy decay bounds in Sect. 6 in order to prove the good gauge formulation of our main result, namely Theorem 2.7.

### The iteration scheme: uniform bounds

Here we seek to construct solutions to (2.35) iteratively, based on the scheme

7.1 $$\begin{aligned} \left\{ \begin{aligned}&(i\partial _t+\partial _{\alpha }g^{(n)\alpha {\beta }}\partial _{{\beta }})\psi ^{(n+1)}+2i(A^{(n)\alpha }-\frac{1}{2}V^{(n)\alpha })\partial _{\alpha }\psi ^{(n+1)}=F^{(n)},\\&\psi (0)=\psi _0, \end{aligned}\right. \end{aligned}$$

with the trivial initialization

$$\begin{aligned} \psi ^{(0)}=0, \end{aligned}$$

where the nonlinearities are

7.2 $$\begin{aligned} F^{(n)}&= \partial _{\alpha }g^{(n)\alpha {\beta }}\cdot \partial _{{\beta }}\psi ^{(n)}+(B^{(n)}+A_{\alpha }^{(n)}A^{(n)\alpha }-V^{(n)\alpha }A^{(n)}_{\alpha })\psi ^{(n)}\nonumber \\&\quad -i\lambda _{\sigma }^{(n)\gamma }\mathop {\mathrm{Im}}\nolimits (\psi ^{(n)}{\bar{\lambda }}^{(n)\sigma }_{\gamma }), \end{aligned}$$

and ${{\mathcal {S}}}^{(n)}=(\lambda ^{(n)},h^{(n)},V^{(n)},A^{(n)},B^{(n)})$ are the solutions of elliptic equations (2.36) with $\psi =\psi ^{(n)}$.

We assume that $\psi _0$ is small in $H^s$. Due to the above trivial initialization, we also inductively assume that

$$\begin{aligned} \Vert \psi ^{(n)}\Vert _{l^2{\mathbf {X}}^s}\le C\Vert \psi _0\Vert _{H^s}, \end{aligned}$$

where *C* is a big constant.

Applying the elliptic estimate (4.14) to (2.36) with $\psi =\psi ^{(n)}$ at each step, we obtain

$$\begin{aligned} \Vert {{\mathcal {S}}}^{(n)}\Vert _{{\varvec{ {\mathcal {E}}}}^{s}}\lesssim \Vert \psi ^{(n)}\Vert _{l^2 {\mathbf {X}}^s}\lesssim \Vert \psi _0\Vert _{H^s}, \end{aligned}$$

Applying at each step the local energy bound (6.16) with $\sigma =s$ we obtain the estimate

$$\begin{aligned} \Vert \psi ^{(n+1)}\Vert _{l^2{\mathbf {X}}^s}&\lesssim \Vert \psi _0\Vert _{H^s}+\Vert F^{(n)}\Vert _{l^2N^s\cap L^2H^{s-2}}\\&\lesssim \Vert \psi _0\Vert _{H^s}+\Vert {{\mathcal {S}}}^{(n)}\Vert _{{\varvec{ {\mathcal {E}}}}^{s}}(1+\Vert {{\mathcal {S}}}^{(n)}\Vert _{{\varvec{ {\mathcal {E}}}}^{s}})\Vert \psi ^{(n)}\Vert _{l^2X^s}\\&\lesssim \Vert \psi _0\Vert _{H^s}+(C\Vert \psi _0\Vert _{H^s})^2(1+C\Vert \psi _0\Vert _{H^s}). \end{aligned}$$

Here the nonlinear terms in $F^{(n)}$ are estimated using (5.1), (5.7), (5.4), (5.5) and (5.6) with $\sigma =s$. Since $\psi _0$ is small in $H^s$, the above bound gives

7.3 $$\begin{aligned} \begin{aligned} \Vert \psi ^{(n+1)}\Vert _{l^2{\mathbf {X}}^s}\le C\Vert \psi _0\Vert _{H^s}, \end{aligned} \end{aligned}$$

which closes our induction.

### The iteration scheme: weak convergence

Here we prove that our iteration scheme converges in the weaker $H^{s-1}$ topology. We denote the differences by

$$\begin{aligned} \Psi ^{(n+1)}= & {} \psi ^{(n+1)}-\psi ^{(n)},\\ \delta {{\mathcal {S}}}^{(n+1)}= & {} (\Lambda ^{(n+1)},{{\mathcal {G}}}^{(n+1)},{{\mathcal {V}}}^{(n+1)},{{\mathcal {A}}}^{(n+1)},{{\mathcal {B}}}^{(n+1)})={{\mathcal {S}}}^{(n+1)}-{{\mathcal {S}}}^{(n)} \end{aligned}$$

Then from (7.1) we obtain the system

$$\begin{aligned} \left\{ \begin{aligned}&i\partial _t \Psi ^{(n+1)}+\partial _\alpha (g^{(n)\alpha {\beta }}\partial _{{\beta }}\Psi ^{(n+1)})+2i(A^{(n)\alpha }-\frac{1}{2}V^{(n)\alpha })\partial _{\alpha }\Psi ^{(n+1)}=F^{(n)}-F^{(n-1)}+G^{(n)},\\&\Psi ^{(n+1)}(0,x)=0, \end{aligned}\right. \end{aligned}$$

where the nonlinearities $G^{(n)}$ have the form

$$\begin{aligned} G^{(n)}&= -\partial _\alpha ({{\mathcal {G}}}^{(n)}\partial _{{\beta }}\psi ^{(n)})-2i({{\mathcal {A}}}^{(n)\alpha }-\frac{1}{2}{{\mathcal {V}}}^{(n)\alpha })\partial _{\alpha }\psi ^{(n)}, \end{aligned}$$

By (4.16) we obtain

$$\begin{aligned} \Vert \delta {{\mathcal {S}}}^{(n)}\Vert _{{\varvec{ {\mathcal {E}}}}^{s-1}}\lesssim \Vert \Psi ^{(n)}\Vert _{l^2{\mathbf {X}}^{s-1}}. \end{aligned}$$

Applying (6.18) with $\sigma =s-1$ for the $\Psi ^{(n+1)}$ equation we have

$$\begin{aligned} \Vert \Psi ^{(n+1)}\Vert _{l^2{\mathbf {X}}^{s-1}}&\lesssim \Vert F^{(n)}-F^{(n-1)}\Vert _{l^2N^{s-1}\cap L^2H^{s-3}}+\big (\Vert {{\mathcal {G}}}^{(n)}\Vert _{{\mathbf {Y}}^{s+1}}\\&\quad +\Vert ({{\mathcal {V}}}^{(n)},{{\mathcal {A}}}^{(n)})\Vert _{{\mathbf {Z}}^{1,s}}\big )\Vert \psi ^{(n)}\Vert _{l^2X^{s}}. \end{aligned}$$

Then by (5.1), (5.7), (5.9), (5.10) and (5.11) with $\sigma =s-1$ we bound the right hand side above by

$$\begin{aligned} \Vert \Psi ^{(n+1)}\Vert _{l^2{\mathbf {X}}^{s-1}}&\lesssim C\Vert \psi _0\Vert _{H^s}\Vert (\Psi ^{(n)},\delta {{\mathcal {S}}}^{(n)})\Vert _{l^2{\mathbf {X}}^{s-1}\times {\varvec{ {\mathcal {E}}}}^{s-1}}\ll \Vert \Psi ^{(n)}\Vert _{l^2{\mathbf {X}}^{s-1}}. \end{aligned}$$

This implies that our iterations $\psi ^{(n)}$ converge in $l^2{\mathbf {X}}^{s-1}$ to some function $\psi$. Furthermore, by the uniform bound (7.3) it follows that

7.4 $$\begin{aligned} \Vert \psi \Vert _{l^2{\mathbf {X}}^s}\lesssim \Vert \psi _0\Vert _{H^s}. \end{aligned}$$

Interpolating, it follows that $\psi ^{(n)}$ converges to $\psi$ in $l^2{\mathbf {X}}^{s-\epsilon }$ for all $\epsilon > 0$. This allows us to conclude that the auxiliary functions ${{\mathcal {S}}}^{(n)}$ associated to $\psi ^{(n)}$ converge to the functions ${{\mathcal {S}}}$ associated to $\psi$, and also to pass to the limit and conclude that $\psi$ solves the (SMCF) equation (2.35). Thus we have established the existence part of our main theorem.

### Uniqueness via weak Lipschitz dependence

Consider the difference of two solutions

$$\begin{aligned} (\Psi ,\delta {{\mathcal {S}}})=(\psi ^{(1)}-\psi ^{(2)},{{\mathcal {S}}}^{(1)}-{{\mathcal {S}}}^{(2)}). \end{aligned}$$

The $\Psi$ solves an equation of this form

$$\begin{aligned} \left\{ \begin{aligned}&i\partial _t \Psi +\partial _{\alpha }g^{(1)\alpha {\beta }}\partial _{{\beta }}\Psi +2i(A^{(1)\alpha }-\frac{1}{2}V^{(1)\alpha })\partial _{\alpha }\Psi =F^{(1)}-F^{(2)}+G,\\&\Psi (0,x)=\psi ^{(1)}_0(x)-\psi ^{(2)}_0(x), \end{aligned}\right. \end{aligned}$$

where the nonlinearity *G* is

$$\begin{aligned} G&= -\partial _{\alpha }({{\mathcal {G}}}\partial _{{\beta }}\psi ^{(2)})-2i({{\mathcal {A}}}^{\alpha }-\frac{1}{2}{{\mathcal {V}}}^{\alpha })\partial _{\alpha }\psi ^{(2)}. \end{aligned}$$

By (4.16), we have

7.5 $$\begin{aligned} \Vert \delta {{\mathcal {S}}}\Vert _{{\varvec{ {\mathcal {E}}}}^{s-1}}\lesssim \Vert \Psi \Vert _{l^2{\mathbf {X}}^{s-1}}. \end{aligned}$$

Applying (6.18) with $\sigma =s-1$ to the $\Psi$ equation, we obtain the estimate

$$\begin{aligned} \Vert \Psi \Vert _{l^2{\mathbf {X}}^{s-1}}&\lesssim \ \Vert \Psi _0\Vert _{H^{s-1}}+\Vert F^{(1)}-F^{(2)}\Vert _{l^2N^{s-1}\cap L^2H^{s-3}}+(\Vert {{\mathcal {G}}}\Vert _{{\mathbf {Y}}^{s+1}}+\Vert ({{\mathcal {V}}},{{\mathcal {A}}})\Vert _{{\mathbf {Z}}^{1,s}})\Vert \psi ^{(2)}\Vert _{l^2X^{s}}\\&\lesssim \ \Vert \Psi _0\Vert _{H^{s-1}}+C\Vert (\psi ^{(1)}_0,\psi ^{(2)}_0)\Vert _{H^{s}}\Vert (\Psi ,\delta {{\mathcal {S}}})\Vert _{l^2{\mathbf {X}}^{s-1}\times {\varvec{ {\mathcal {E}}}}^{s-1}}. \end{aligned}$$

Then, by the above bound (7.5), we further have

$$\begin{aligned} \Vert \Psi \Vert _{l^2{\mathbf {X}}^{s-1}}\lesssim \Vert \Psi _0\Vert _{H^{s-1}}+C\Vert (\psi ^{(1)}_0,\psi ^{(2)}_0)\Vert _{H^{s}}\Vert \Psi \Vert _{l^2{\mathbf {X}}^{s-1}} \end{aligned}$$

Since the initial data $\psi ^{(1)}_0$ and $\psi ^{(2)}_0$ are sufficiently small, we obtain

7.6 $$\begin{aligned} \Vert \Psi \Vert _{l^2{\mathbf {X}}^{s-1}}\lesssim \Vert \Psi _0\Vert _{H^{s-1}}. \end{aligned}$$

This gives the weak Lipschitz dependence, as well as the uniqueness of solutions for (2.35).

### Frequency envelope bounds

Here we prove a stronger frequency envelope version of estimate (7.4).

#### Proposition 7.1

Let $\psi \in l^2{\mathbf {X}}^s$ be a small data solution to (2.35), which satisfies (7.4). Let $\{p_{0k}\}$ be an admissible frequency envelope for the initial data $\psi _0\in H^s$. Then $\{p_{0k}\}$ is also frequency envelope for $\psi$ in $l^2{\mathbf {X}}^s$.

#### Proof

Let $p_k$ and $s_k$ be the admissible frequency envelopes for solution $(\psi ,{{\mathcal {S}}})\in l^2{\mathbf {X}}^s\times {\varvec{ {\mathcal {E}}}}^s$. Applying $S_k$ to the Schrödinger equation (2.35), we obtain the paradifferential equation

$$\begin{aligned} \left\{ \begin{aligned}&(i\partial _t+\partial _{\alpha }g_{<k-4}^{\alpha {\beta }}\partial _{{\beta }})\psi _k+2i(A-\frac{1}{2}V)_{<k-4}^{\alpha }\partial _{\alpha }\psi _k=F_k+J_k,\\&\psi (0,x)=\psi _0(x), \end{aligned}\right. \end{aligned}$$

where

$$\begin{aligned} J_k&= -S_k\partial _{\alpha }g^{\alpha {\beta }}_{\ge k-4}\partial _{{\beta }}\psi -[S_k,\partial _{\alpha }g^{\alpha {\beta }}_{< k-4}\partial _{{\beta }}]\psi -2i[S_k,(A-\frac{1}{2}V)^{\alpha }_{<k-4}]\partial _{\alpha }\psi \\&-2iS_k[ (A-\frac{1}{2}V)^{\alpha }_{\ge k-4}\partial _{\alpha }\psi _k], \end{aligned}$$

and ${{\mathcal {S}}}=(\lambda ,h,V,A,B)$ is the solution to the elliptic system (2.36). We estimate $\psi _k=S_k\psi$ using Proposition 6.4. By Proposition 5.2, Lemmas 5.1 and 5.3 we obtain

$$\begin{aligned} \Vert \psi _k\Vert _{l^2{\mathbf {X}}^s}\lesssim p_{0k}+p_k\Vert {{\mathcal {S}}}\Vert _{{\varvec{ {\mathcal {E}}}}^s}+s_k \Vert \psi \Vert _{l^2{\mathbf {X}}^s}\lesssim p_{0k}+(p_k+s_k) \Vert \psi \Vert _{l^2{\mathbf {X}}^s}. \end{aligned}$$

Then by (4.15), the definition of frequency envelope (3.3) and (7.4), this implies

$$\begin{aligned} p_k\lesssim p_{0k}+p_k\Vert \psi \Vert _{l^2{\mathbf {X}}^s}. \end{aligned}$$

By the smallness of $\psi \in l^2{\mathbf {X}}^s$, this further gives $p_k\lesssim p_{0k}$, and concludes the proof. $\square$

### Continuous dependence on the initial data

Here we show that the map $\psi _0\rightarrow (\psi ,{{\mathcal {S}}})$ is continuous from $H^s$ into $l^2{\mathbf {X}}^s\times {\varvec{ {\mathcal {E}}}}^s$. By (4.16), it suffices to prove $\psi _0\rightarrow \psi$ is continuous from $H^s$ to $l^2{\mathbf {X}}^s$.

Suppose that $\psi _0^{(n)}\rightarrow \psi _0$ in $H^s$. Denote by $p_{0k}^{(n)}$, respectively $p_{0k}$ the frequency envelopes associated to $\psi _0^{(n)}$, respectively $\psi _0$, given by (3.3). If $\psi _0^{(n)}\rightarrow \psi _0$ in $H^s$ then $p_{0k}^{(n)}\rightarrow p_{0k}$ in $l^2$. Then for each $\epsilon >0$ we can find some $N_{\epsilon }$ so that

$$\begin{aligned} \Vert p_{0,>N_{\epsilon }}^{(n)}\Vert _{l^2}\le \epsilon ,\ \text {for all }n. \end{aligned}$$

By Proposition 7.1 we obtain that

7.7 $$\begin{aligned} \Vert \psi _{>N_{\epsilon }}^{(n)}\Vert _{l^2{\mathbf {X}}^s}\le \epsilon ,\ \text {for all }n. \end{aligned}$$

To compare $\psi ^{(n)}$ with $\psi$ we use (7.6) for low frequencies and (7.7) for the high frequencies,

$$\begin{aligned} \Vert \psi ^{(n)}-\psi \Vert _{l^2{\mathbf {X}}^s}&\lesssim \Vert S_{<N_{\epsilon }}(\psi ^{(n)}-\psi )\Vert _{l^2{\mathbf {X}}^s}+\Vert S_{>N_{\epsilon }}\psi ^{(n)}\Vert _{l^2X^s}+\Vert S_{>N_{\epsilon }}\psi \Vert _{l^2X^s}\\&\lesssim 2^{N_{\epsilon }}\Vert S_{<N_{\epsilon }}(\psi ^{(n)}-\psi )\Vert _{l^2{\mathbf {X}}^{s-1}}+2\epsilon \\&\lesssim 2^{N_{\epsilon }}\Vert S_{<N_{\epsilon }}(\psi ^{(n)}_0-\psi _0)\Vert _{H^{s-1}}+2\epsilon . \end{aligned}$$

Letting $n\rightarrow \infty$ we obtain

$$\begin{aligned} \limsup _{n\rightarrow \infty }\Vert \psi ^{(n)}-\psi \Vert _{l^2{\mathbf {X}}^s}\lesssim \epsilon . \end{aligned}$$

Letting $\epsilon \rightarrow 0$ we obtain

$$\begin{aligned} \lim _{n\rightarrow 0}\Vert \psi ^{(n)}-\psi \Vert _{l^2{\mathbf {X}}^s}=0, \end{aligned}$$

which completes the desired result.

### Higher regularity

Here we prove that the solution $(\psi ,{{\mathcal {S}}})$ satisfies the bound

7.8 $$\begin{aligned} \Vert (\psi ,{{\mathcal {S}}})\Vert _{l^2{\mathbf {X}}^{\sigma }\times {\varvec{ {\mathcal {E}}}}^{\sigma }}\lesssim \Vert \psi _0\Vert _{H^{\sigma }},\quad \sigma \ge s, \end{aligned}$$

whenever the right hand side is finite.

Differentiating the original Schrödinger equation (2.35) yields

$$\begin{aligned} (i\partial _t+\partial _{\alpha }g^{\alpha {\beta }}\partial _{{\beta }})\nabla \psi +2i(A-\frac{V}{2})^{\alpha }\partial _{\alpha }\nabla \psi =-\partial _{\alpha }(\nabla g^{\alpha {\beta }}\partial _{{\beta }}\psi )-2i\nabla A^{\alpha }\partial _{\alpha }\psi +\nabla F, \end{aligned}$$

where *F* is defined as in (7.2) without superscript (*n*). Using Proposition 6.5 we obtain

$$\begin{aligned} \Vert \nabla \psi \Vert _{l^2{\mathbf {X}}^s}\lesssim \Vert \nabla \psi _0\Vert _{H^s}+\Vert (\nabla \psi ,\nabla {{\mathcal {S}}})\Vert _{l^2{\mathbf {X}}^s\times {\varvec{ {\mathcal {E}}}}^s}\Vert (\psi ,{{\mathcal {S}}})\Vert _{l^2{\mathbf {X}}^s\times {\varvec{ {\mathcal {E}}}}^s}(1+\Vert (\psi ,{{\mathcal {S}}})\Vert _{l^2{\mathbf {X}}^s\times {\varvec{ {\mathcal {E}}}}^s})^N. \end{aligned}$$

For elliptic equations, by (4.16) we obtain

$$\begin{aligned} \Vert \nabla {{\mathcal {S}}}\Vert _{{\varvec{ {\mathcal {E}}}}^s}\lesssim \Vert \nabla \psi \Vert _{l^2{\mathbf {X}}^s}. \end{aligned}$$

Hence, by (7.4) and the smallness of $\psi _0$ in $H^s$, these imply

$$\begin{aligned} \Vert (\nabla \psi ,\nabla {{\mathcal {S}}})\Vert _{l^2{\mathbf {X}}^{s}\times {\varvec{ {\mathcal {E}}}}^s}\lesssim \Vert \nabla \psi _0\Vert _{H^{s}}. \end{aligned}$$

Inductively, we can obtain the system for $(\nabla ^n\psi ,\nabla ^n{{\mathcal {S}}})$. This leads to

$$\begin{aligned} \Vert (\nabla ^n\psi ,\nabla ^n{{\mathcal {S}}})\Vert _{l^2{\mathbf {X}}^s\times {\varvec{ {\mathcal {E}}}}^s}\lesssim \Vert \psi _0\Vert _{H^{s+n}}+\Vert \psi \Vert _{l^2{\mathbf {X}}^{s+n}}\Vert \psi \Vert _{l^2 {\mathbf {X}}^s}(1+\Vert \psi \Vert _{l^2 {\mathbf {X}}^s})^N, \end{aligned}$$

which shows that

$$\begin{aligned} \Vert (\psi ,{{\mathcal {S}}})\Vert _{l^2{\mathbf {X}}^{s+n}\times {\varvec{ {\mathcal {E}}}}^{s+n}}\lesssim \Vert \psi _0\Vert _{H^{s+n}}+\Vert \psi \Vert _{l^2{\mathbf {X}}^{s+n}}\Vert \psi \Vert _{l^2 {\mathbf {X}}^s}(1+\Vert \psi \Vert _{l^2 {\mathbf {X}}^s})^N, \end{aligned}$$

and hence gives the bound (7.8) by the smallness of $\psi$ in $l^2{\mathbf {X}}^s$.

### The time evolution of $(\lambda ,g,A)$

As part of our derivation of the (SMCF) equations (2.35) for the mean curvature $\psi$ in the good gauge, coupled with the elliptic system (2.36), we have seen that the time evolution of $(\lambda ,g,A)$ is described by the equations (2.31), (2.26) and (2.32). However, our proof of the well-posedness result for the Schrödinger evolution (2.35) does not apriori guarantee that (2.31), (2.26) and (2.32) hold. Here we rectify this omission:

#### Lemma 7.2

Assume that $\psi \in C[0,T;H^s]$ solves the SMCF equation (2.35) coupled with the elliptic system (2.36). Then the relations (2.26), (2.31) and (2.32) hold.

#### Proof

We recall that, by Theorem 4.1, the solution ${{\mathcal {S}}}= (\lambda ,h,V,A,B)$ in ${\mathcal {H}}^s$ for the system (2.36) satisfies the fixed time constraints (2.4), (2.8), (2.15), (2.13), (2.19) and (2.16). On the other hand, in terms of the time evolution, at this point we only have the equation (2.35) for the mean curvature $\psi$. We will show that this implies (2.26), (2.31) and (2.32).

To shorten the notations, we define the tensors

$$\begin{aligned}&T^1_{\alpha {\beta }}=\partial _t g_{\alpha {\beta }}-2\mathop {\mathrm{Im}}\nolimits (\psi {\bar{\lambda }}_{\alpha {\beta }})-\nabla _{\alpha }V_{{\beta }}-\nabla _{{\beta }}V_{\alpha },\\&T^{2,\sigma }_{\alpha }=(\partial ^{B}_t-V^\gamma \nabla ^A_\gamma )\lambda ^{\sigma }_{\alpha }-i\nabla ^A_{\alpha }\nabla ^{A,\sigma } \psi +\lambda ^{\gamma }_{\alpha }\mathop {\mathrm{Im}}\nolimits (\psi {\bar{\lambda }}^{\sigma }_{\gamma }) +\lambda _{\alpha \gamma }\nabla ^\gamma V^\sigma -\lambda _\gamma ^\sigma \nabla _\alpha V^\gamma ,\\&T^3_\alpha =\partial _t A_{\alpha }-\partial _{\alpha } B-\mathop {\mathrm{Re}}\nolimits (\lambda _{\alpha }^{\gamma }{\bar{\partial }}^A_{\gamma }{\bar{\psi }})+\mathop {\mathrm{Im}}\nolimits (\lambda ^\gamma _\alpha {\bar{\lambda }}_{\gamma \sigma })V^\sigma . \end{aligned}$$

We need to show that $T^1=0$, $T^2 = 0$, $T^3 = 0$. To do this, we will show that $(T^1,T^2,T^3)$ solve a linear homogeneous coupled elliptic system of the form

$$\begin{aligned} \left\{ \begin{aligned}&\Delta _g T^1 = \nabla (\Gamma T^1)+ \lambda ^2 T^1 + \lambda T^2 , \\&\nabla ^{A,\alpha } T_\alpha ^{2,\sigma } = \lambda T^3 + \lambda \nabla T^1 + T^1 \nabla \lambda , \\&\nabla ^{A}_{\alpha } T_{\beta }^{2,\sigma }- \nabla ^{A}_{{\beta }} T_\alpha ^{2,\sigma } = \lambda T^3 + \lambda \nabla T^1 + T^1 \nabla \lambda , \\&\nabla ^\alpha T^3_\alpha = T^1 \nabla A, \\&\nabla _\alpha T^3_{\beta }-\nabla ^{\beta }T^3_\alpha = \lambda T^2. \end{aligned} \right. \end{aligned}$$

Considering this system for $(T^1,T^2,T^3) \in \dot{H}^1 \times L^2 \times L^2$, the smallness condition on the coefficients $(\lambda ,h,V,A,B ) \in {{\mathcal {S}}}$ insures that this system has the unique solution $(T^1,T^2,T^3)=0$. It remains to derive the system for $(T^1,T^2,T^3)$.

*The equation for* $T^1$ This has the form

7.9 $$\begin{aligned} \begin{aligned} \Delta _g T^1_{\alpha {\beta }}&= \ T^1_{\delta {\beta }} {{{\,\mathrm{Ric}\,}}^\delta }_\alpha +T^1_{\delta \alpha } {{{\,\mathrm{Ric}\,}}^\delta }_{\beta }+2T^{1,\mu \nu }R_{\alpha \mu {\beta }\nu }-\nabla _{\beta }(T^{1,\mu \nu }\Gamma _{\mu \nu ,\alpha })-\nabla _\alpha (T^{1,\mu \nu }\Gamma _{\mu \nu ,{\beta }})\\&-2\mathop {\mathrm{Re}}\nolimits (g_{\sigma {\beta }}T^{2,\sigma }_\alpha {\bar{\psi }}+T^1_{\sigma {\beta }}\lambda ^\sigma _\alpha \psi +{\bar{\lambda }}_{\alpha {\beta }}T^{2,\sigma }_\sigma -g_{\sigma \mu }T^{2,\sigma }_\alpha {\bar{\lambda }}^\mu _{\beta }-T^1_{\sigma \mu }\lambda ^\sigma _\alpha {\bar{\lambda }}^\mu _{\beta }-{\bar{\lambda }}_{\alpha \sigma }T^{2,\sigma }_{\beta }). \end{aligned}\nonumber \\ \end{aligned}$$

We start with the first term in $T^1$, and compute the expression $\Delta _g \partial _t g_{\alpha {\beta }}$. We have

$$\begin{aligned} \Delta _g \partial _t g_{\alpha {\beta }}&= \ g^{\mu \nu }(\partial _\mu \nabla _\nu \partial _t g_{\alpha {\beta }}-\Gamma ^\delta _{\nu \alpha }\nabla _\nu \partial _t g_{\delta {\beta }}-\Gamma ^\delta _{\mu {\beta }}\nabla _\nu \partial _t g_{\alpha \delta })\\&= \ [\partial _t (g^{\mu \nu }\partial _\mu \partial _\nu g_{\alpha {\beta }})-\partial _tg^{\mu \nu }\partial _\mu \partial _\nu g_{\alpha {\beta }}] +[-g^{\mu \nu }\Gamma ^\delta _{\nu \alpha }\partial _\mu \partial _t g_{\delta {\beta }}-g^{\mu \nu }\Gamma ^\delta _{\nu {\beta }}\partial _\mu \partial _t g_{\delta \alpha }\\&\ -g^{\mu \nu }\partial _\mu \Gamma ^\delta _{\nu \alpha }\partial _t g_{\delta {\beta }}-g^{\mu \nu }\partial _\mu \Gamma ^\delta _{\nu {\beta }}\partial _t g_{\delta \alpha }-g^{\mu \nu }(\Gamma ^\delta _{\mu \alpha }\nabla _\nu \partial _t g_{\delta {\beta }}+\Gamma ^\delta _{\mu {\beta }}\nabla _\nu \partial _t g_{\delta \alpha })]\\ :&= \ I+II. \end{aligned}$$

We then use covariant derivatives to write *II* as

$$\begin{aligned} II&= -g^{\mu \nu }\Gamma ^\delta _{\mu \alpha }(2\nabla _\nu \partial _t g_{\delta {\beta }}+\Gamma ^\sigma _{\nu \delta }\partial _t g_{\sigma {\beta }}+\Gamma ^\sigma _{\nu {\beta }}\partial _t g_{\sigma \delta })\\&\quad -g^{\mu \nu }\Gamma ^\delta _{\mu {\beta }}(2\nabla _\nu \partial _t g_{\delta \alpha }+\Gamma ^\sigma _{\nu \delta }\partial _t g_{\sigma \alpha }+\Gamma ^\sigma _{\nu \alpha }\partial _t g_{\sigma \delta })\\&\quad -g^{\mu \nu }\partial _\mu \Gamma ^\delta _{\nu \alpha }\partial _t g_{\delta {\beta }}-g^{\mu \nu }\partial _\mu \Gamma ^\delta _{\nu {\beta }}\partial _t g_{\delta \alpha }\\&= -2g^{\mu \nu }\Gamma ^\delta _{\mu \alpha }\nabla _\nu \partial _t g_{\delta {\beta }}-2g^{\mu \nu }\Gamma ^\delta _{\mu {\beta }}\nabla _\nu \partial _t g_{\delta \alpha }\\&\quad -\partial _t g_{\delta {\beta }} g^{\mu \nu }(\partial _\mu \Gamma ^\delta _{\nu \alpha }+\Gamma ^\sigma _{\mu \alpha }\Gamma ^\delta _{\nu \sigma })-\partial _t g_{\delta \alpha } g^{\mu \nu }(\partial _\mu \Gamma ^\delta _{\nu {\beta }}+\Gamma ^\sigma _{\mu {\beta }}\Gamma ^\delta _{\nu \sigma })\\&\quad -2\partial _t g_{\sigma \delta }g^{\mu \nu }\Gamma ^\delta _{\mu \alpha }\Gamma ^\sigma _{\nu {\beta }}. \end{aligned}$$

For *I*, by the *g* equation (2.22) we have

$$\begin{aligned} I&= \ \partial _t[-\partial _\alpha g^{\mu \nu }\partial _\mu g_{\nu {\beta }}-\partial _{\beta }g^{\mu \nu }\partial _\mu g_{\nu \alpha }+\partial _\alpha g^{\mu \nu }\partial _{\beta }g_{\mu \nu }]\\&\quad +[2\partial _t(g^{\mu \nu }\Gamma _{\mu \alpha ,\delta }\Gamma ^\delta _{\nu {\beta }})-\partial _t g^{\mu \nu }\partial _\mu \partial _\nu g_{\alpha {\beta }}]-2\partial _t {{\,\mathrm{{\widetilde{Ric}}}\,}}_{\alpha {\beta }}\\&: = \ I_1+I_2+I_3. \end{aligned}$$

The expression $I_1$ is written as

$$\begin{aligned} I_1&= -\partial _\alpha \partial _t g^{\mu \nu }\Gamma _{\mu \nu ,{\beta }}-\partial _{\beta }\partial _t g^{\mu \nu }\Gamma _{\mu \nu ,\alpha }-\frac{1}{2}\partial _{\beta }\partial _t g^{\mu \nu }\partial _\alpha g_{\mu \nu }+\frac{1}{2}\partial _\alpha \partial _t g^{\mu \nu }\partial _{\beta }g_{\mu \nu }\\&\quad -\partial _\alpha g^{\mu \nu }\partial _\mu \partial _t g_{\nu {\beta }}-\partial _{\beta }g^{\mu \nu }\partial _\mu \partial _t g_{\nu \alpha }+\partial _\alpha g^{\mu \nu }\partial _{\beta }\partial _t g_{\mu \nu }\\&= -(\nabla _\alpha \partial _t g^{\mu \nu }-2\Gamma ^\mu _{\alpha \delta }\partial _t g^{\delta \nu })\Gamma _{\mu \nu ,{\beta }}-(\nabla _{\beta }\partial _t g^{\mu \nu }-2\Gamma ^\mu _{{\beta }\delta }\partial _t g^{\delta \nu })\Gamma _{\mu \nu ,\alpha }\\&\quad +\frac{1}{2}[\nabla _\alpha (\partial _t g^{\mu \nu }\partial _{\beta }g_{\mu \nu }-\nabla _{\beta }(\partial _t g^{\mu \nu }\partial _\alpha g_{\mu \nu })]\\&\quad -\partial _\alpha g^{\mu \nu }(\nabla _\mu \partial _t g_{\nu {\beta }}+\Gamma ^\delta _{\mu \nu }\partial _t g_{\delta {\beta }})-\partial _{\beta }g^{\mu \nu }(\nabla _\mu \partial _t g_{\nu \alpha }+\Gamma ^\delta _{\mu \nu }\partial _t g_{\delta \alpha }+\Gamma ^\delta _{\mu \alpha }\partial _t g_{\nu \delta })\\&\quad +\partial _\alpha g^{\mu \nu }(\nabla _{\beta }\partial _t g_{\mu \nu }+\Gamma ^\delta _{{\beta }\nu }\partial _t g_{\mu \delta })\\&= \ \nabla _\alpha \partial _t g^{\mu \nu } (-\Gamma _{\mu \nu ,{\beta }}+\Gamma _{\mu {\beta },\nu })+\nabla _{\beta }\partial _t g^{\mu \nu }(-\Gamma _{\mu \nu ,\alpha }+\Gamma _{\mu \alpha ,\nu })\\&\quad -\nabla _\mu \partial _t g_{\nu {\beta }}\partial _\alpha g^{\mu \nu }-\nabla _\mu \partial _t g_{\nu \alpha }\partial _{\beta }g^{\mu \nu }\\&\quad +2\partial _t g^{\mu \nu }(\Gamma ^\delta _{\alpha \mu }\Gamma _{\delta \nu ,{\beta }}+\Gamma ^\delta _{{\beta }\mu }\Gamma _{\delta \nu ,\alpha })+\partial _t g^{\mu \nu }(-\Gamma ^\delta _{\alpha \mu }\partial _{\beta }g_{\delta \nu }+\Gamma ^\delta _{{\beta }\mu }\partial _\alpha g_{\delta \nu })\\&\quad +\partial _t g^{\mu \nu }(-\partial _{\beta }g_{\mu \sigma } g^{\sigma \delta }\Gamma _{\delta \alpha ,\nu }+\partial _\alpha g_{\mu \sigma } g^{\sigma \delta }\Gamma _{\delta {\beta },\nu })\\&\quad -\partial _t g_{\delta {\beta }}\partial _\alpha g^{\mu \nu }\Gamma ^\delta _{\mu \nu }-\partial _t g_{\delta \alpha }\partial _{\beta }g^{\mu \nu }\Gamma ^\delta _{\mu \nu } \end{aligned}$$

For $I_2$, we first compute

$$\begin{aligned} 2g^{\mu \nu }\partial _t(\Gamma _{\mu \alpha ,\delta }\Gamma ^\delta _{\nu {\beta }})&= g^{\mu \nu }\Gamma ^\delta _{\nu {\beta }}(\nabla _\mu \partial _t g_{\alpha \delta }+\nabla _\alpha \partial _t g_{\mu \delta }-\nabla _\delta \partial _t g_{\mu \alpha })+4g^{\mu \nu }\Gamma ^\delta _{\nu {\beta }}\Gamma ^\sigma _{\alpha \mu }\partial _t g_{\sigma \delta }\\&\quad +g^{\mu \nu }\Gamma ^\delta _{\nu \alpha }(\nabla _\mu \partial _t g_{{\beta }\delta }+\nabla _{\beta }\partial _t g_{\mu \delta }-\nabla _\delta \partial _t g_{\mu {\beta }})+2\partial _t g^{\sigma \delta }g^{\mu \nu }\Gamma _{\mu \alpha ,\delta }\Gamma _{\nu {\beta },\sigma } \end{aligned}$$

By the above computations, we collect the $\nabla \partial _t g$ terms from $I_1$, $I_2$ and *II*

$$\begin{aligned}&\nabla _\alpha \partial _t g^{\mu \nu } (-\Gamma _{\mu \nu ,{\beta }}+\Gamma _{\mu {\beta },\nu })+\nabla _{\beta }\partial _t g^{\mu \nu }(-\Gamma _{\mu \nu ,\alpha }+\Gamma _{\mu \alpha ,\nu })-\nabla _\mu \partial _t g_{\nu {\beta }}\partial _\alpha g^{\mu \nu }-\nabla _\mu \partial _t g_{\nu \alpha }\partial _{\beta }g^{\mu \nu }\\&\quad +g^{\mu \nu }\Gamma ^\delta _{\nu {\beta }}(\nabla _\mu \partial _t g_{\alpha \delta }+\nabla _\alpha \partial _t g_{\mu \delta }-\nabla _\delta \partial _t g_{\mu \alpha }) +g^{\mu \nu }\Gamma ^\delta _{\nu \alpha }(\nabla _\mu \partial _t g_{{\beta }\delta }+\nabla _{\beta }\partial _t g_{\mu \delta }-\nabla _\delta \partial _t g_{\mu {\beta }})\\&\quad -2g^{\mu \nu }\Gamma ^\delta _{\mu \alpha }\nabla _\nu \partial _t g_{\delta {\beta }}-2g^{\mu \nu }\Gamma ^\delta _{\mu {\beta }}\nabla _\nu \partial _t g_{\delta \alpha }, \end{aligned}$$

where the terms containing $\nabla \partial _t g_{\nu \alpha }$ and $\nabla \partial _t g_{\nu {\beta }}$ vanish, i.e.

$$\begin{aligned} \nabla _\mu \partial _t g_{\nu {\beta }}(-\partial _\alpha g^{\mu \nu }-g^{\nu \delta }\Gamma ^\mu _{\delta \alpha }-g^{\delta \mu }\Gamma ^\nu _{\delta \alpha })+\nabla _\mu \partial _t g_{\nu \alpha }(-\partial _{\beta }g^{\mu \nu }-g^{\nu \delta }\Gamma ^\mu _{\delta {\beta }}-g^{\mu \delta }\Gamma ^\nu _{\delta {\beta }})=0, \end{aligned}$$

and the terms with $\nabla \partial _t g^{\mu \nu }$ were rewritten as

7.10 $$\begin{aligned} \begin{aligned}&-\nabla _{\alpha }\partial _t g^{\mu \nu }\Gamma _{\mu \nu ,{\beta }}-\nabla _{{\beta }}\partial _t g^{\mu \nu }\Gamma _{\mu \nu ,\alpha }\\&= -\nabla _{\alpha }(\partial _t g^{\mu \nu }\Gamma _{\mu \nu ,{\beta }})-\nabla _{{\beta }}(\partial _t g^{\mu \nu }\Gamma _{\mu \nu ,\alpha })+\partial _t g^{\mu \nu }(\nabla _{\alpha }\Gamma _{\mu \nu ,{\beta }}+\nabla _{\beta }\Gamma _{\mu \nu ,\alpha }) \end{aligned} \end{aligned}$$

We collect the $\partial _t g$ terms from *I* and *II* into

$$\begin{aligned}&2\partial _t g^{\mu \nu }(\Gamma ^\delta _{\alpha \mu }\Gamma _{\delta \nu ,{\beta }}+\Gamma ^\delta _{{\beta }\mu }\Gamma _{\delta \nu ,\alpha }) -\partial _t g_{\delta {\beta }}\partial _\alpha g^{\mu \nu }\Gamma ^\delta _{\mu \nu }-\partial _t g_{\delta \alpha }\partial _{\beta }g^{\mu \nu }\Gamma ^\delta _{\mu \nu }\\&+\partial _t g^{\mu \nu }(2\Gamma _{\mu \alpha ,\delta }\Gamma ^\delta _{\nu {\beta }}-\partial _\mu \partial _\nu g_{\alpha {\beta }})\\&-\partial _t g_{\delta {\beta }} g^{\mu \nu }(\partial _\mu \Gamma ^\delta _{\nu \alpha }+\Gamma ^\sigma _{\mu \alpha }\Gamma ^\delta _{\nu \sigma })-\partial _t g_{\delta \alpha } g^{\mu \nu }(\partial _\mu \Gamma ^\delta _{\nu {\beta }}+\Gamma ^\sigma _{\mu {\beta }}\Gamma ^\delta _{\nu \sigma }). \end{aligned}$$

Adding the $\partial _t g$ terms together with the third term in (7.10) we obtain

$$\begin{aligned}&\partial _t g^{\mu \nu }(\nabla _{\alpha }\Gamma _{\mu \nu ,{\beta }}+\nabla _{\beta }\Gamma _{\mu \nu ,\alpha }+2\Gamma ^\delta _{\alpha \mu }\Gamma _{\delta \nu ,{\beta }}+2\Gamma ^\delta _{{\beta }\mu }\Gamma _{\delta \nu ,\alpha }+2\Gamma _{\mu \alpha ,\delta }\Gamma ^\delta _{\nu {\beta }}-\partial _\mu \partial _\nu g_{\alpha {\beta }})\\&= \partial _t g^{\mu \nu }(\partial _{\alpha }\Gamma _{\mu \nu ,{\beta }}+\partial _{\beta }\Gamma _{\mu \nu ,\alpha }-\partial _\nu (\Gamma _{{\beta }\mu ,\alpha }+\Gamma _{\alpha \mu ,{\beta }})+2\Gamma _{\mu \alpha ,\delta }\Gamma ^\delta _{\nu {\beta }}-2\Gamma _{\alpha {\beta }}^\delta \Gamma _{\mu \nu ,\delta })\\&= 2\partial _t g^{\mu \nu }R_{\alpha \mu {\beta }\nu }. \end{aligned}$$

Finally, using the harmonic coordinate condition $g^{\mu \nu }\Gamma ^\delta _{\mu \nu }=0$, the terms containing the $\partial _t g_{\delta \alpha }$ expression are written as

$$\begin{aligned}&-\partial _t g_{\delta {\beta }}\partial _\alpha g^{\mu \nu }\Gamma ^\delta _{\mu \nu }-\partial _t g_{\delta \alpha }\partial _{\beta }g^{\mu \nu }\Gamma ^\delta _{\mu \nu } -\partial _t g_{\delta {\beta }} g^{\mu \nu }(\partial _\mu \Gamma ^\delta _{\nu \alpha }+\Gamma ^\sigma _{\mu \alpha }\Gamma ^\delta _{\nu \sigma })\\&\quad -\partial _t g_{\delta \alpha } g^{\mu \nu }(\partial _\mu \Gamma ^\delta _{\nu {\beta }}+\Gamma ^\sigma _{\mu {\beta }}\Gamma ^\delta _{\nu \sigma })\\&\qquad \qquad = \partial _t g_{\delta {\beta }} {{{\,\mathrm{Ric}\,}}^\delta }_\alpha +\partial _t g_{\delta \alpha }{{{\,\mathrm{Ric}\,}}^\delta }_{\beta }. \end{aligned}$$

Hence, the expression $\Delta _g\partial _t g_{\alpha {\beta }}$ is written as

7.11 $$\begin{aligned} \Delta _g \partial _t g_{\alpha {\beta }}&= -\nabla _{\alpha }(\partial _t g^{\mu \nu }\Gamma _{\mu \nu ,{\beta }})-\nabla _{{\beta }}(\partial _t g^{\mu \nu }\Gamma _{\mu \nu ,\alpha })+ \partial _t g_{\delta {\beta }}{{{\,\mathrm{Ric}\,}}^\delta }_\alpha +\partial _t g_{\delta \alpha }{{{\,\mathrm{Ric}\,}}^\delta }_{\beta }\nonumber \\&\quad +2\partial _t g^{\mu \nu }R_{\alpha \mu {\beta }\nu }-2\partial _t {{\,\mathrm{{\widetilde{Ric}}}\,}}_{\alpha {\beta }}. \end{aligned}$$

For the last term $-2\partial _t {{\,\mathrm{{\widetilde{Ric}}}\,}}_{\alpha {\beta }}$, using the expression $T^2$ we have 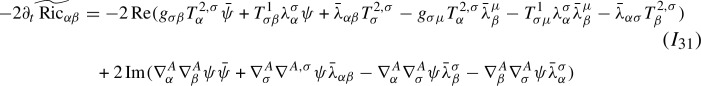

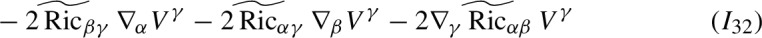

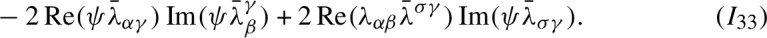


Next, we compute

$$\begin{aligned} III:&= \ -\Delta _g(2\mathop {\mathrm{Im}}\nolimits (\psi {\bar{\lambda }}_{\alpha {\beta }})+\nabla _\alpha V_{\beta }+\nabla _{\beta }V_\alpha )\\&= \ -2\nabla ^\sigma \nabla _\sigma \mathop {\mathrm{Im}}\nolimits (\psi {\bar{\lambda }}_{\alpha {\beta }})+[\Delta _g,\nabla _\alpha ] V_{\beta }-[\Delta _g,\nabla _{\beta }] V_\alpha -\nabla _\alpha \Delta _g V_{\beta }-\nabla _{\beta }\Delta _g V_\alpha \\&= \ -2\nabla ^\sigma \nabla _\sigma \mathop {\mathrm{Im}}\nolimits (\psi {\bar{\lambda }}_{\alpha {\beta }})-\nabla _{\beta }{{\,\mathrm{Ric}\,}}_{\alpha \gamma }V^\gamma -\nabla _\alpha {{\,\mathrm{Ric}\,}}_{{\beta }\gamma }V^\gamma -2\nabla _\gamma {{\,\mathrm{Ric}\,}}_{\alpha {\beta }} V^\gamma \\&\quad -{{\,\mathrm{Ric}\,}}_{\alpha \gamma }\nabla ^\gamma V_{\beta }-{{\,\mathrm{Ric}\,}}_{{\beta }\gamma }\nabla ^\gamma V_\alpha +2R_{\alpha \sigma {\beta }\delta }(\nabla ^\sigma V^\delta +\nabla ^\delta V^\sigma ) -\nabla _\alpha \Delta _g V_{\beta }-\nabla _{\beta }\Delta _g V_\alpha \end{aligned}$$

Using the *V*-equation (2.30) we write the last two terms as

$$\begin{aligned}&-\nabla _\alpha \Delta _g V_{\beta }-\nabla _{\beta }\Delta _g V_\alpha \\&= 2\nabla _\alpha \nabla _\sigma \mathop {\mathrm{Im}}\nolimits (\psi {\bar{\lambda }}^\sigma _{\beta })+2\nabla _{\beta }\nabla _\sigma \mathop {\mathrm{Im}}\nolimits (\psi {\bar{\lambda }}^\sigma _\alpha )+\nabla _\alpha {{\,\mathrm{Ric}\,}}_{\sigma {\beta }} V^\sigma +\nabla _{\beta }{{\,\mathrm{Ric}\,}}_{\sigma \alpha }V^\sigma \\&\quad + {{\,\mathrm{Ric}\,}}_{\sigma {\beta }}\nabla _\alpha V^\sigma +{{\,\mathrm{Ric}\,}}_{\sigma \alpha }\nabla _{\beta }V^\sigma +\nabla _\alpha (\widetilde{\partial _t g^{\mu \nu }}\Gamma _{\mu \nu ,{\beta }})+\nabla _{\beta }(\widetilde{\partial _t g^{\mu \nu }}\Gamma _{\mu \nu ,\alpha }), \end{aligned}$$

where $\widetilde{\partial _t g^{\mu \nu }}$ denotes the expression

$$\begin{aligned} \widetilde{\partial _t g^{\mu \nu }}\,{:}{=}\,\partial _t g^{\mu \nu }-T^{1,\mu \nu }. \end{aligned}$$

We then add $I_{31}$ together with $\nabla ^2\mathop {\mathrm{Im}}\nolimits (\psi \lambda )$ in *III* to get

$$\begin{aligned}&I_{31}-2\nabla ^\sigma \nabla _\sigma \mathop {\mathrm{Im}}\nolimits (\psi {\bar{\lambda }}_{\alpha {\beta }})+2\nabla _\alpha \nabla _\sigma \mathop {\mathrm{Im}}\nolimits (\psi {\bar{\lambda }}^\sigma _{\beta })+2\nabla _{\beta }\nabla _\sigma \mathop {\mathrm{Im}}\nolimits (\psi {\bar{\lambda }}^\sigma _\alpha )\\&= -2{{\,\mathrm{Ric}\,}}_{{\beta }\delta }\mathop {\mathrm{Im}}\nolimits (\psi {\bar{\lambda }}^\delta _\alpha )+2R_{\alpha \sigma {\beta }\delta }\mathop {\mathrm{Im}}\nolimits (\psi {\bar{\lambda }}^{\sigma \delta })+2\mathop {\mathrm{Re}}\nolimits (\lambda _\alpha ^\sigma {\bar{\psi }})\mathop {\mathrm{Im}}\nolimits (\lambda _{\sigma \mu }{\bar{\lambda }}^\mu _{\beta }). \end{aligned}$$

The last term and $I_{33}$ can be further written as

$$\begin{aligned}&2\mathop {\mathrm{Re}}\nolimits (\lambda _\alpha ^\sigma {\bar{\psi }})\mathop {\mathrm{Im}}\nolimits (\lambda _{\sigma \mu }{\bar{\lambda }}^\mu _{\beta })+I_{33}\\&= 2R_{\alpha \sigma {\beta }\delta }\mathop {\mathrm{Im}}\nolimits (\psi {\bar{\lambda }}^{\sigma \delta })-2{{\,\mathrm{Ric}\,}}_{\alpha \delta }\mathop {\mathrm{Im}}\nolimits (\psi {\bar{\lambda }}^\delta _{\beta }). \end{aligned}$$

Hence, given the expressions of $I_{3}$ and *III*, we obtain

$$\begin{aligned}&I_3+III\\&= -2\mathop {\mathrm{Re}}\nolimits (g_{\sigma {\beta }}T^{2,\sigma }_\alpha {\bar{\psi }}+T^1_{\sigma {\beta }}\lambda ^\sigma _\alpha \psi +{\bar{\lambda }}_{\alpha {\beta }}T^{2,\sigma }_\sigma -g_{\sigma \mu }T^{2,\sigma }_\alpha {\bar{\lambda }}^\mu _{\beta }-T^1_{\sigma \mu }\lambda ^\sigma _\alpha {\bar{\lambda }}^\mu _{\beta }-{\bar{\lambda }}_{\alpha \sigma }T^{2,\sigma }_{\beta })\\&\quad -{{{\,\mathrm{Ric}\,}}^\sigma }_{\beta }\widetilde{\partial _t g_{\alpha \sigma }}-{{{\,\mathrm{Ric}\,}}^\sigma }_\alpha \widetilde{\partial _t g_{{\beta }\sigma }}-2R_{\alpha \sigma {\beta }\delta }\widetilde{\partial _t g^{\sigma \delta }}+\nabla _\alpha (\widetilde{\partial _t g^{\mu \nu }}\Gamma _{\mu \nu ,{\beta }})+\nabla _{\beta }(\widetilde{\partial _t g^{\mu \nu }}\Gamma _{\mu \nu ,\alpha }), \end{aligned}$$

which combined with (7.11) yields the $T^1$-equation (7.9). $\square$

*The equation for* $T^2$ This has the form

7.12 $$\begin{aligned} \left\{ \begin{aligned}&\begin{aligned} \nabla ^{A,\alpha }T^{2,\sigma }_\alpha&= ig^{\sigma \mu }\psi T^3_\mu -i\lambda ^{{\beta }\sigma }T^3_{\beta }+g^{\sigma \delta }\lambda ^{\alpha {\beta }} (-\nabla _\alpha T^1_{{\beta }\delta }+\frac{1}{2}\nabla _\delta T^1_{\alpha {\beta }})\\&\quad -T^{1,\alpha {\beta }}(\nabla ^A_{\beta }\lambda ^\sigma _\alpha +\Gamma ^\mu _{\alpha {\beta }} \lambda ^\sigma _\mu )+T^{1,\sigma \mu }\nabla ^A_\mu \psi , \end{aligned}\\&\begin{aligned} \nabla ^A_\alpha T^{2,\sigma }_{\beta }-\nabla ^A_{\beta }T^{2,\sigma }_\alpha&= \frac{1}{2} g^{\sigma \gamma }[-\lambda ^\mu _{\beta }(\nabla _\alpha T^1_{\mu \gamma }+\nabla _\mu T^1_{\alpha \gamma }-\nabla _\gamma T^1_{\alpha \mu })\\&\quad +\lambda ^\mu _\alpha (\nabla _{\beta }T^1_{\mu \gamma }+\nabla _\mu T^1_{{\beta }\gamma }-\nabla _\gamma T^1_{{\beta }\mu })]-iT^3_\alpha \lambda ^\sigma _{\beta }+iT^3_{\beta }\lambda ^\sigma _\alpha . \end{aligned} \end{aligned}\right. \end{aligned}$$

We compute the divergence of $T^2$ in (7.12) first. Applying $\nabla ^{A,\alpha }$ to $T^{2,\sigma }_\alpha$, we have

$$\begin{aligned} \nabla ^{A,\alpha }T^{2,\sigma }_\alpha&= [\nabla ^{A,\alpha },\partial _t^B-V^\gamma \nabla ^A_\gamma ]\lambda ^\sigma _\alpha +[\partial _t^B-V^\gamma \nabla ^A_\gamma ,\nabla ^{A,\sigma }]\psi +\nabla ^{A,\sigma }(\partial _t^B-V^\gamma \nabla ^A_\gamma ) \psi \\&\quad +\nabla ^{A,\alpha }(\lambda _\alpha ^\gamma \mathop {\mathrm{Im}}\nolimits (\psi {\bar{\lambda }}^\sigma _\gamma ))-i\nabla ^{A,\alpha }\nabla ^A_\alpha \nabla ^{A,\sigma }\psi \\&\quad +\nabla ^{A}_\gamma \psi \nabla ^\gamma V^\sigma -\nabla ^{A,\sigma }\lambda _{\alpha \gamma }\nabla ^\alpha V^\gamma +\lambda ^{\alpha \gamma }\nabla _\alpha \nabla _\gamma V^\sigma -\lambda ^\sigma _\gamma \Delta _g V^\gamma . \end{aligned}$$

Three of the terms on the right-hand side are written as

$$\begin{aligned}&[\nabla ^{A,\alpha },\partial _t^B-V^\gamma \nabla ^A_\gamma ]\lambda ^\sigma _\alpha -\nabla ^{A,\sigma }\lambda _{\alpha \gamma }\nabla ^\alpha V^\gamma +\lambda ^{\alpha \gamma }\nabla _\alpha \nabla _\gamma V^\sigma \\&= g^{\alpha {\beta }}(\nabla _{\beta }\partial _t \lambda ^\sigma _\alpha -\partial _t \nabla _{\beta }\lambda ^\sigma _\alpha )+ig^{\alpha {\beta }}(\nabla _{\beta }B-\partial _t A_{\beta })\lambda ^\sigma _\alpha -\partial _t g^{\alpha {\beta }} \nabla ^A_{{\beta }} \lambda ^\sigma _\alpha \\&\quad +\lambda ^{\alpha \gamma }\nabla _\alpha \nabla _\gamma V^\sigma -2\nabla ^{A,\sigma }\lambda _{\alpha \gamma }\nabla ^\alpha V^\gamma -V^\gamma [\nabla ^{\alpha },\nabla _\gamma ]\lambda ^\sigma _\alpha -iV_\gamma {\mathbf {F}}^{\alpha \gamma }\lambda ^\sigma _\alpha \\&= -\partial _t g^{\alpha {\beta }} (\nabla ^{A,\sigma } \lambda _{\alpha {\beta }}+\Gamma ^\mu _{\alpha {\beta }} \lambda ^\sigma _\mu )-\partial _t\Gamma ^\sigma _{{\beta }\mu }\lambda ^{{\beta }\mu }+\lambda ^{\alpha \gamma }\nabla _\alpha \nabla _\gamma V^\sigma \\&\quad -i(\partial _t A_{\beta }-\nabla _{\beta }B)\lambda ^{{\beta }\sigma }-iV_\gamma {\mathbf {F}}^{\alpha \gamma }\lambda ^\sigma _\alpha -2\nabla ^{A,\sigma }\lambda _{\alpha \gamma }\nabla ^\alpha V^\gamma -V^\gamma [\nabla ^{\alpha },\nabla _\gamma ]\lambda ^\sigma _\alpha \end{aligned}$$

We can further use $T^1$ to rewrite the last two terms on the first line above as

$$\begin{aligned}&-\partial _t \Gamma ^\sigma _{\alpha {\beta }}\lambda ^{\alpha {\beta }}+\lambda ^{\alpha \gamma }\nabla _\alpha \nabla _\gamma V^\sigma \\&= -\partial _t g^{\sigma \delta }\Gamma _{\alpha {\beta },\delta }\lambda ^{\alpha {\beta }}-g^{\sigma \delta }\partial _t(\partial _\alpha g_{{\beta }\delta }-\frac{1}{2}\partial _\delta g_{\alpha {\beta }})\lambda ^{\alpha {\beta }}+\lambda ^{\alpha \gamma }\nabla _\alpha \nabla _\gamma V^\sigma \\&= g^{\sigma \delta }\lambda ^{\alpha {\beta }}(\partial _t g_{\mu \delta }\Gamma _{\alpha {\beta }}^\mu -\partial _{\alpha }\partial _t g_{{\beta }\delta }+\frac{1}{2}\partial _\delta \partial _t g_{\alpha {\beta }})+\lambda ^{\alpha \gamma }\nabla _\alpha \nabla _\gamma V^\sigma \\&= g^{\sigma \delta }\lambda ^{\alpha {\beta }} (-\nabla _\alpha \partial _t g_{{\beta }\delta }+\frac{1}{2}\nabla _\delta \partial _t g_{\alpha {\beta }})+\lambda ^{\alpha \gamma }\nabla _\alpha \nabla _\gamma V^\sigma \\&= \lambda _{\mu \nu } (\nabla ^\mu T^{1,\nu \sigma }-\frac{1}{2}\nabla ^\sigma T^{1,\mu \nu })\\&\quad +\lambda ^{\alpha {\beta }}[-2\nabla _\alpha \mathop {\mathrm{Im}}\nolimits (\psi {\bar{\lambda }}^{\sigma }_{\beta })+\nabla ^\sigma \mathop {\mathrm{Im}}\nolimits (\psi {\bar{\lambda }}_{\alpha {\beta }})-[\nabla _\alpha ,\nabla ^{\sigma }]V_{{\beta }}] \end{aligned}$$

and the following term as

$$\begin{aligned} -i(\partial _t A_{\beta }-\nabla _{\beta }B)\lambda ^{{\beta }\sigma }-iV_\gamma {\mathbf {F}}^{\alpha \gamma }\lambda ^\sigma _\alpha&= -i\lambda ^{{\beta }\sigma }T^3_{\beta }-i\lambda ^{{\beta }\sigma }\mathop {\mathrm{Re}}\nolimits (\lambda _{\beta }^\gamma \overline{\nabla ^A_\gamma \psi }). \end{aligned}$$

Similarly, we compute the second commutator by

$$\begin{aligned}{}[\partial _t^B-V^\gamma \nabla ^A_\gamma ,\nabla ^{A,\sigma }]\psi +\nabla ^{A}_\gamma \psi \nabla ^\gamma V^\sigma&= \partial _t g^{\sigma \mu } \nabla ^A_\mu \psi +ig^{\sigma \mu }\psi T^3_\mu +i\psi \mathop {\mathrm{Re}}\nolimits (\lambda ^{\sigma \gamma }\overline{\nabla ^A_\gamma \psi })\\&\quad +\nabla ^A_\gamma \psi (\nabla ^\gamma V^\sigma +\nabla ^\sigma V^\gamma ). \end{aligned}$$

Hence, using $T^{2,\alpha }_\alpha$ and the *V* equation (2.30) we reorganize the expression of $\nabla ^{A,\alpha }T^{2,\sigma }_\alpha$ and obtain

$$\begin{aligned} \nabla ^{A,\alpha }T^{2,\sigma }_\alpha&= ig^{\sigma \mu }\psi T^3_\mu -i\lambda ^{{\beta }\sigma }T^3_{\beta }+g^{\sigma \delta }\lambda ^{\alpha {\beta }} (-\nabla _\alpha T^1_{{\beta }\delta }+\frac{1}{2}\nabla _\delta T^1_{\alpha {\beta }})\\&\quad -\partial _t g^{\alpha {\beta }} (\nabla ^A_{\beta }\lambda ^\sigma _\alpha +\Gamma ^\mu _{\alpha {\beta }} \lambda ^\sigma _\mu ) +\lambda ^{\alpha {\beta }}[-2\nabla _\alpha \mathop {\mathrm{Im}}\nolimits (\psi {\bar{\lambda }}^{\sigma }_{\beta })+\nabla ^\sigma \mathop {\mathrm{Im}}\nolimits (\psi {\bar{\lambda }}_{\alpha {\beta }})]\\&\quad -i\lambda ^{{\beta }\sigma }\mathop {\mathrm{Re}}\nolimits (\lambda _{\beta }^\gamma \overline{\nabla ^A_\gamma \psi }) +\partial _t g^{\sigma \mu } \nabla ^A_\mu \psi +i\psi \mathop {\mathrm{Re}}\nolimits (\lambda ^{\sigma \gamma }\overline{\nabla ^A_\gamma \psi })\\&\quad -\nabla ^{A,\sigma }(\lambda ^\gamma _\alpha \mathop {\mathrm{Im}}\nolimits (\psi {\bar{\lambda }}^\alpha _\gamma )) +\nabla ^{A,\alpha }(\lambda _\alpha ^\gamma \mathop {\mathrm{Im}}\nolimits (\psi {\bar{\lambda }}^\sigma _\gamma ))\\&\quad -i{{{\,\mathrm{Ric}\,}}^\sigma }_\delta \nabla ^{A,\delta }\psi -\nabla _\alpha {\mathbf {F}}^{\sigma \alpha }\psi -2{\mathbf {F}}^{\sigma \alpha }\nabla ^A_\alpha \psi \\&\quad +\nabla ^{A}_\gamma \psi (\nabla ^\gamma V^\sigma +\nabla ^\sigma V^\gamma ) -2\nabla ^{A,\sigma }\lambda _{\alpha \gamma }\nabla ^\alpha V^\gamma \\&\quad -2\lambda ^\sigma _\gamma \nabla _\alpha \mathop {\mathrm{Im}}\nolimits (\lambda ^{\alpha \gamma }{\bar{\psi }})+\lambda ^\sigma _\gamma \widetilde{\partial _t g^{\alpha {\beta }}}\Gamma _{\alpha {\beta }}^\gamma . \end{aligned}$$

Using $T^{2,\alpha }_\alpha$ and the *V*-equation (2.30), we have

$$\begin{aligned}&\lambda ^{\alpha {\beta }}[-2\nabla _\alpha \mathop {\mathrm{Im}}\nolimits (\psi {\bar{\lambda }}^{\sigma }_{\beta })+\nabla ^\sigma \mathop {\mathrm{Im}}\nolimits (\psi {\bar{\lambda }}_{\alpha {\beta }})]-i\lambda ^{{\beta }\sigma }\mathop {\mathrm{Re}}\nolimits (\lambda _{\beta }^\gamma \overline{\nabla ^A_\gamma \psi })\\&\quad +i\psi \mathop {\mathrm{Re}}\nolimits (\lambda ^{\sigma \gamma }\overline{\nabla ^A_\gamma \psi })+\nabla ^{A,\alpha }(\lambda _\alpha ^\gamma \mathop {\mathrm{Im}}\nolimits (\psi {\bar{\lambda }}^\sigma _\gamma ))-i{{{\,\mathrm{Ric}\,}}^\sigma }_\delta \nabla ^{A,\delta }\psi -\nabla _\alpha {\mathbf {F}}^{\sigma \alpha }\psi -2{\mathbf {F}}^{\sigma \alpha }\nabla ^A_\alpha \psi \\&\quad +2\lambda ^\sigma _\gamma \nabla ^\alpha \mathop {\mathrm{Im}}\nolimits (\psi {\bar{\lambda }}^\gamma _\alpha )-\nabla ^{A,\sigma }(\lambda ^\gamma _\alpha \mathop {\mathrm{Im}}\nolimits (\psi {\bar{\lambda }}^\alpha _\gamma ))\\&= 2\nabla ^A_\gamma \psi \mathop {\mathrm{Im}}\nolimits (\psi {\bar{\lambda }}^{\sigma \gamma })-2\nabla ^{A,\sigma }\lambda _{\alpha {\beta }}\mathop {\mathrm{Im}}\nolimits (\psi {\bar{\lambda }}^{\alpha {\beta }}) \end{aligned}$$

Combining these two expressions, we obtain

$$\begin{aligned} \nabla ^{A,\alpha }T^{2,\sigma }_\alpha&= ig^{\sigma \mu }\psi T^3_\mu -i\lambda ^{{\beta }\sigma }T^3_{\beta }+g^{\sigma \delta }\lambda ^{\alpha {\beta }} (-\nabla _\alpha T^1_{{\beta }\delta }+\frac{1}{2}\nabla _\delta T^1_{\alpha {\beta }})\\&\quad -T^{1,\alpha {\beta }}(\nabla ^A_{\beta }\lambda ^\sigma _\alpha +\Gamma ^\mu _{\alpha {\beta }} \lambda ^\sigma _\mu )+T^{1,\sigma \mu }\nabla ^A_\mu \psi +\nabla ^{A,\sigma } T^{2,\alpha }_\alpha . \end{aligned}$$

Next we compute the *curl* of $T^2$ in (7.12). By $T^2$ we have

$$\begin{aligned}&\nabla ^A_\alpha T^{2,\sigma }_{\beta }-\nabla ^A_{\beta }T^{2,\sigma }_\alpha \\&= [\nabla ^A_\alpha ,\partial _t^B-V^\gamma \nabla ^A_\gamma ]\lambda ^\sigma _{\beta }-[\nabla ^A_{\beta },\partial _t^B-V^\gamma \nabla ^A_\gamma ]\lambda ^\sigma _\alpha +\lambda ^\gamma _{\beta }\nabla _\alpha \mathop {\mathrm{Im}}\nolimits (\psi {\bar{\lambda }}^\sigma _\gamma )-\lambda ^\gamma _\alpha \nabla _{\beta }\mathop {\mathrm{Im}}\nolimits (\psi {\bar{\lambda }}^\sigma _\gamma )\\&\quad -i[\nabla ^A_\alpha ,\nabla ^A_{\beta }]\nabla ^{A,\sigma }\psi +\lambda ^\gamma _{\beta }\nabla _\alpha \nabla _\gamma V^\sigma -\lambda ^\gamma _\alpha \nabla _{\beta }\nabla _\gamma V^\sigma \\&\quad -\lambda ^\sigma _\gamma [\nabla _\alpha ,\nabla _{\beta }]V^\gamma -\nabla ^A_\gamma \lambda ^\sigma _\alpha \nabla _{\beta }V^\gamma +\nabla ^A_\gamma \lambda ^\sigma _{\beta }\nabla _\alpha V^\gamma . \end{aligned}$$

We use $T^1$ and $T^3$ to rewrite six of the terms on the right-hand side as

$$\begin{aligned}&[\nabla ^A_\alpha ,\partial _t^B-V^\gamma \nabla ^A_\gamma ]\lambda ^\sigma _{\beta }-[\nabla ^A_{\beta },\partial _t^B-V^\gamma \nabla ^A_\gamma ]\lambda ^\sigma _\alpha +\lambda ^\gamma _{\beta }\nabla _\alpha \nabla _\gamma V^\sigma -\lambda ^\gamma _\alpha \nabla _{\beta }\nabla _\gamma V^\sigma \\&\quad -\nabla ^A_\gamma \lambda ^\sigma _\alpha \nabla _{\beta }V^\gamma +\nabla ^A_\gamma \lambda ^\sigma _{\beta }\nabla _\alpha V^\gamma \\&= \frac{1}{2} g^{\sigma \gamma }[-\lambda ^\mu _{\beta }(\nabla _\alpha T^1_{\mu \gamma }+\nabla _\mu T^1_{\alpha \gamma }-\nabla _\gamma T^1_{\alpha \mu })+\lambda ^\mu _\alpha (\nabla _{\beta }T^1_{\mu \gamma }+\nabla _\mu T^1_{{\beta }\gamma }-\nabla _\gamma T^1_{{\beta }\mu })]\\&\quad -iT^3_\alpha \lambda ^\sigma _{\beta }+iT^3_{\beta }\lambda ^\sigma _\alpha \\&\quad -\lambda ^\mu _{\beta }\nabla _\alpha \mathop {\mathrm{Im}}\nolimits (\psi {\bar{\lambda }}^\sigma _\mu )+\lambda ^\mu _\alpha \nabla _{\beta }\mathop {\mathrm{Im}}\nolimits (\psi {\bar{\lambda }}^\sigma _\mu )+I_1+I_2, \end{aligned}$$

where $I_1$ and $I_2$ are

$$\begin{aligned} I_1:&= \lambda ^\mu _{\beta }(-\nabla _\mu \mathop {\mathrm{Im}}\nolimits (\psi {\bar{\lambda }}^\sigma _\alpha )+\nabla ^\sigma \mathop {\mathrm{Im}}\nolimits (\psi {\bar{\lambda }}_{\alpha \mu }))-\lambda ^\mu _\alpha (-\nabla _\mu \mathop {\mathrm{Im}}\nolimits (\psi {\bar{\lambda }}^\sigma _{\beta })+\nabla ^\sigma \mathop {\mathrm{Im}}\nolimits (\psi {\bar{\lambda }}_{{\beta }\mu }))\\&\quad -i\mathop {\mathrm{Re}}\nolimits (\lambda ^\gamma _\alpha \overline{\nabla ^A_\gamma \psi })\lambda ^\sigma _{\beta }+i\mathop {\mathrm{Re}}\nolimits (\lambda ^\gamma _{\beta }\overline{\nabla ^A_\gamma \psi })\lambda ^\sigma _\alpha ,\\ I_2:&= \frac{1}{2}\lambda ^\mu _{\beta }(R_{\alpha \mu \sigma \delta }+R^\sigma _{\alpha \mu \delta }+R^\sigma _{\mu \alpha \delta })V^\delta -\frac{1}{2}\lambda ^\mu _\alpha (R_{{\beta }\mu \sigma \delta }+R^\sigma _{{\beta }\mu \delta }+R^\sigma _{\mu {\beta }\delta })V^\delta \\&\quad -V^\gamma R_{\alpha \gamma \sigma \delta }\lambda ^\delta _{\beta }-V^\gamma R_{\alpha \gamma {\beta }\delta }\lambda ^{\sigma \delta }+V^\gamma R_{{\beta }\gamma \sigma \delta }\lambda ^\delta _\alpha +V^\gamma R_{{\beta }\gamma \alpha \delta }\lambda ^{\sigma \delta } . \end{aligned}$$

Then we use Bianchi identities and compatibility conditions to compute $I_1$ and $I_2$ by

$$\begin{aligned} I_1=i[\nabla ^A_\alpha ,\nabla ^A_{\beta }]\nabla ^{A,\sigma }\psi \end{aligned}$$

and

$$\begin{aligned} I_2=V^\gamma R_{{\beta }\gamma \sigma \delta }\lambda ^\delta _\alpha +V^\gamma R_{{\beta }\gamma \alpha \delta }\lambda ^{\sigma \delta }=\lambda ^\sigma _\gamma [\nabla _\alpha ,\nabla _{\beta }]V^\gamma . \end{aligned}$$

Hence, we obtain

$$\begin{aligned} \nabla ^A_\alpha T^{2,\sigma }_{\beta }-\nabla ^A_{\beta }T^{2,\sigma }_\alpha&= \frac{1}{2} g^{\sigma \gamma }[-\lambda ^\mu _{\beta }(\nabla _\alpha T^1_{\mu \gamma }+\nabla _\mu T^1_{\alpha \gamma }-\nabla _\gamma T^1_{\alpha \mu })\\&\quad +\lambda ^\mu _\alpha (\nabla _{\beta }T^1_{\mu \gamma }+\nabla _\mu T^1_{{\beta }\gamma }-\nabla _\gamma T^1_{{\beta }\mu })]-iT^3_\alpha \lambda ^\sigma _{\beta }+iT^3_{\beta }\lambda ^\sigma _\alpha . \end{aligned}$$

This completes the derivation of (7.12). $\square$

*The equation for* $T^3$ This has the form

$$\begin{aligned} \left\{ \begin{aligned}&\nabla ^\alpha T^3_{\alpha }=-T^{1,\alpha {\beta }}\partial _\alpha A_{\beta },\\&\nabla _\alpha T^3_{\beta }-\nabla _{\beta }T^3_\alpha =\mathop {\mathrm{Im}}\nolimits (T^{2,\sigma }_{\ \alpha }{\bar{\lambda }}_{\sigma {\beta }}+\lambda ^\sigma _\alpha \overline{T^{2}_{\sigma {\beta }}}). \end{aligned}\right. \end{aligned}$$

Applying $\nabla ^\alpha$ to $T^3_\alpha$, we then use the Coulomb condition $\nabla ^\alpha A_\alpha =0$ and the *B*-equation (2.33) to get

$$\begin{aligned} \nabla ^\alpha T^3_\alpha&= \nabla ^\alpha \partial _t A_\alpha -\Delta _g B-\nabla ^\alpha \mathop {\mathrm{Re}}\nolimits (\lambda ^\sigma _\alpha \overline{\nabla ^A_\sigma \psi }+i\lambda ^\sigma _\alpha {\bar{\lambda }}_{\sigma \gamma }V^\gamma )\\&= g^{\alpha {\beta }}\partial _{\beta }\partial _t A_\alpha +\partial _t g^{{\beta }\gamma }\partial _{\beta }A_\gamma -T^{1,{\beta }\gamma }\partial _{\beta }A_\gamma =-T^{1,\alpha {\beta }}\partial _\alpha A_{\beta }. \end{aligned}$$

The *curl* of $T^3$ is obtained by (2.13) directly. $\square$

$$\square $$

## The Reconstruction of the Flow

In this last section we close the circle of ideas in this paper, and prove that one can start from the good gauge solution given by Theorem 2.7, and reconstruct the flow at the level of *d*-dimensional embedded submanifolds. For completeness, we provide here another, more complete statement of our main theorem:

### Theorem 8.1

(Small data local well-posedness). Let $s>\frac{d}{2}$, $d\ge 4$. Consider the skew mean curvature flow (1.1) for maps *F* from ${{\mathbb {R}}}^d$ to the Euclidean space $({{\mathbb {R}}}^{d+2},g_{{{\mathbb {R}}}^{d+2}})$ with initial data $\Sigma _0$ which, in some coordinates, has a metric $g_0$ satisfying $\Vert \partial _x(g_0-I_d)\Vert _{H^{s}}\le \epsilon _0$ and mean curvature $\Vert {\mathbf {H}}_0 \Vert _{H^s(\Sigma _0)}\le \epsilon _0$.

If $\epsilon _0>0$ is sufficiently small, then there exists a unique solution

$$\begin{aligned} F: {{\mathbb {R}}}^d \times [0,1] \rightarrow ({{\mathbb {R}}}^{d+2},g_{{{\mathbb {R}}}^{d+2}}) \end{aligned}$$

which, when represented in harmonic coordinates, has regularity

$$\begin{aligned} \partial _x^2 F,\ \partial _t F \in C([0,1]; H^{s}({{\mathbb {R}}}^d)). \end{aligned}$$

and induced metric and mean curvature

$$\begin{aligned} \partial _x g \in C([0,1]; H^{s+1}({{\mathbb {R}}}^d)), \quad {\mathbf {H}} \in C([0,1]; H^{s}({{\mathbb {R}}}^d)). \end{aligned}$$

In addition the mean curvature satisfies the bounds

$$\begin{aligned} \Vert \psi \Vert _{l^2 {\mathbf {X}}^s} + \Vert (\lambda ,h,V,A,B)\Vert _{{\varvec{ {\mathcal {E}}}}^s}\lesssim \Vert \psi _0\Vert _{H^s}. \end{aligned}$$

where $\psi$ and $\lambda$ are expressed using the Coulomb gauge in the normal bundle $N \Sigma _t$.

We complement the theorem with the following remarks:

### Remark 8.1.1

Here uniqueness should be interpreted in two steps:

(i) If $s > \frac{d}{2}+1$ then we have a direct uniqueness statement for solutions *F* which in some coordinate system are continuous with values in $H^{s+2}$.
(ii) For smaller *s*, then our solutions can be identified as the unique limits of smooth solutions expressed in harmonic coordinates.

### Remark 8.1.2

The only role of the smallness condition on the metric is to exclude large nonflat minimal surfaces; the topology we use there is less essential as long as some critical norm of *F* is made small. This guarantees that (i) we can find harmonic coordinates on the surface $\Sigma _0$ and a Coulomb frame in the normal bundle and (ii) in harmonic coordinates and the Coulomb gauge the surface is uniquely (and smoothly) determined by the mean curvature $\psi$ up to rigid rotations.

We do this in several steps:

###  The starting point

Our evolution begins at time $t=0$, where we need to represent the initial submanifold as parametrized with global harmonic coordinates, represented via the map $F: {{\mathbb {R}}}^d \rightarrow {{\mathbb {R}}}^{d+2}$, and to construct a Coulomb frame in the normal bundle, leading to the complex mean curvature function $\psi$. This is the goal of this subsection, which is carried out in Proposition 8.2.

Once this is done, we have the frame $F_\alpha$ in the tangent space and the frame *m* in the normal bundle. In turn, as described in Sect. 2, these generate the metric *g*, the second fundamental form $\lambda$ with trace $\psi$ and the connection *A*, all at the initial time $t=0$.

Moving forward in time, Theorem 2.7 provides us with the time evolution of $\psi$ via the Schödinger flow (2.35), as well as the functions $(\lambda , g, V, A,B)$ satisfying the elliptic system (2.36) together with the constraints (2.4), (2.8), (2.15), (2.13), (2.16) and (2.19) and the time evolutions (2.26), (2.31) and (2.32). The objective of the rest of this section is then to use these functions in order to reconstruct the map *F* which describes the manifold *F* at later times.

We now return to the question of constructing the harmonic coordinates at the initial time. In order to state the following proposition, we define some notations. Let $F:{{\mathbb {R}}}^d_x \rightarrow ({{\mathbb {R}}}^{d+2},g_{{{\mathbb {R}}}^{d+2}})$ be an immersion with induced metric *g*(*x*). For any change of coordinate $y=x+\phi (x)$, we denote

$$\begin{aligned} \tilde{F}(y)=F(x(y)), \end{aligned}$$

and its induced metric $\tilde{g}_{\alpha {\beta }}(y)=\langle \partial _{y_\alpha }{\tilde{F}},\partial _{y_{\beta }}{\tilde{F}}\rangle$. We also denote its Christoffel symbol as ${\tilde{\Gamma }}$ and $\tilde{h}(y)={\tilde{g}}(y)-I_d$.

#### Proposition 8.2

Let $d\ge 3$, $s>\frac{d}{2}$, and $F:({{\mathbb {R}}}^d_x,g)\rightarrow ({{\mathbb {R}}}^{d+2},g_{{{\mathbb {R}}}^{d+2}})$ be an immersion with induced metric $g=I_d+h$. Assume that $\nabla h(x)$ and mean curvature ${\mathbf {H}}$ are small in $H^{s}(dx)$, namely

$$\begin{aligned} \Vert \partial _x h\Vert _{H^s}\le \epsilon _0,\quad \Vert {\mathbf {H}}\Vert _{H^s}\le \epsilon _0. \end{aligned}$$

Then there exists a unique change of coordinates $y=x+\phi (x)$ with $\lim _{x\rightarrow \infty }\phi (x)=0$ and $\nabla \phi$ uniformly small, such that the new coordinates $\{y_1,\ldots ,y_d\}$ are global harmonic coordinates, namely,

$$\begin{aligned} {\tilde{g}}^{\alpha {\beta }}(y)\tilde{\Gamma }_{\alpha {\beta }}^\gamma (y)=0,\quad \text {for any }y\in {{\mathbb {R}}}^d. \end{aligned}$$

Moreover,

8.1 $$\begin{aligned} \Vert \nabla ^{2}\phi (x)\Vert _{H^{s}(dx)}\lesssim \Vert \nabla h(x)\Vert _{H^{s}(dx)}, \end{aligned}$$

and, in the new coordinates $\{y_1,\ldots ,y_d\}$,

8.2 $$\begin{aligned} \Vert \partial _y {\tilde{h}}\Vert _{H^{s}(dy)}\lesssim \Vert \partial _x h\Vert _{H^s(dx)}. \end{aligned}$$

In addition, for the mean curvature we have equivalent norms,

8.3 $$\begin{aligned} \Vert {\mathbf {H}}\Vert _{H^{s}(dy)}\lesssim \Vert {\mathbf {H}}\Vert _{H^s(dx)}, \end{aligned}$$

and the bound for complex scalar mean curvature $\psi$ in the Coulomb gauge

8.4 $$\begin{aligned} \Vert \psi \Vert _{H^s}\lesssim \epsilon _0. \end{aligned}$$

#### Proof

*Step 1: Derivation of the* $\phi$-*equations.*

We make the following change of coordinates such that the $\{y_1,\ldots ,y_d\}$ is a global harmonic coordinate

$$\begin{aligned} \begin{matrix} {{\mathbb {R}}}^d &{}\longrightarrow &{} {{\mathbb {R}}}^d &{}\longrightarrow &{}{{\mathbb {R}}}^{d+2}\\ y&{}\longmapsto &{} x&{}\longmapsto &{} F(x(y))=\tilde{F}(y) \end{matrix} \end{aligned}$$

where $x+\phi (x)=y$ with $\lim _{x\rightarrow \infty }\phi (x)=0$ and $\nabla \phi$ small.

To determine the function $\phi$, we perform a few computations. For any vector $f=(f_1,\ldots ,f_d)$, we denote

$$\begin{aligned} \frac{\partial f}{\partial x}= \left( \begin{array}{lll} \frac{\partial f_1}{\partial x_1}&{} \ldots &{}\frac{\partial f_1}{\partial x_d}\\ \vdots &{} \ddots &{}\vdots \\ \frac{\partial f_d}{\partial x_1}&{} \ldots &{}\frac{\partial f_d}{\partial x_d} \end{array} \right) . \end{aligned}$$

Then we have

$$\begin{aligned} \frac{\partial x}{\partial y}+\frac{\partial \phi }{\partial x}\frac{\partial x}{\partial y}=I_d. \end{aligned}$$

This implies that

$$\begin{aligned} \frac{\partial x}{\partial y}=I_d-\frac{\partial \phi }{\partial x}+{{\mathcal {C}}}(x), \end{aligned}$$

where the matrix ${\mathcal {C}}(x)$ is a higher order term which satisfies

$$\begin{aligned} {\mathcal {C}}(x)=(\frac{\partial \phi }{\partial x})^2-{\mathcal {C}}(x) \frac{\partial \phi }{\partial x}, \end{aligned}$$

or, equivalently, it is given by

$$\begin{aligned} {\mathcal {C}}(x)=(\frac{\partial \phi }{\partial x})^2(I-\frac{\partial \phi }{\partial x})^{-1}. \end{aligned}$$

We denote

$$\begin{aligned} {{\mathcal {P}}}^\mu _\alpha \,{:}{=}\, -\frac{\partial \phi _\mu }{\partial x_\alpha }+{\mathcal {C}}_{\mu \alpha }(x). \end{aligned}$$

Since $\tilde{F}(y)=F(x(y))$, then we have

8.5 $$\begin{aligned} \begin{aligned} \tilde{g}_{\alpha {\beta }}(y)&= \langle \frac{\partial {\tilde{F}}}{\partial y_\alpha },\frac{\partial {\tilde{F}}}{\partial y_{\beta }}\rangle =\langle \frac{\partial F}{\partial x_\mu }\frac{\partial x_\mu }{\partial y_\alpha },\frac{\partial F}{\partial x_\nu }\frac{\partial x_\nu }{\partial y_{\beta }}\rangle \\&= g_{\mu \nu }(x)(\delta ^\mu _\alpha -\partial _\alpha \phi _\mu +{\mathcal {C}}_{\mu \alpha })(\delta ^\nu _{\beta }-\partial _{\beta }\phi _\nu +{\mathcal {C}}_{\nu {\beta }}) \end{aligned} \end{aligned}$$

and

8.6 $$\begin{aligned} \tilde{g}^{\alpha {\beta }}(y)&= g^{\mu \nu }\frac{\partial y_\alpha }{\partial x_\mu }\frac{\partial y_{\beta }}{\partial x_\nu }=g^{\mu \nu }(\delta ^\alpha _\mu +\partial _\mu \phi _\alpha )(\delta ^{\beta }_\nu +\partial _\nu \phi _{\beta }). \end{aligned}$$

We also have

8.7 $$\begin{aligned} \frac{\partial \tilde{g}_{\alpha {\beta }}(y)}{\partial y_\gamma }=\frac{\partial \tilde{g}_{\alpha {\beta }}(y)}{\partial x_m}\frac{\partial x_m}{\partial y_\gamma } = -g_{\mu {\beta }}\partial ^2_{\alpha \gamma }\phi _\mu -g_{\alpha \nu }\partial ^2_{{\beta }\gamma }\phi _\nu +\partial _\gamma g_{\alpha {\beta }}+{\mathcal {K}}_{\alpha {\beta },\gamma }, \end{aligned}$$

where the higher order terms ${\mathcal {K}}_{\alpha {\beta },\gamma }$ are defined as

$$\begin{aligned} {\mathcal {K}}_{\alpha {\beta },\gamma }:&= -g_{\mu \nu }\partial ^2_{\alpha \gamma }\phi _\mu {\mathcal {P}}^\nu _{\beta }+g_{\mu \nu }\partial _\gamma {\mathcal {C}}_{\mu \alpha }\frac{\partial x_\nu }{\partial y_{\beta }} -g_{\mu \nu }{{\mathcal {P}}}^\mu _\alpha \partial ^2_{{\beta }\gamma }\phi _\nu +g_{\mu \nu }\frac{\partial x_\mu }{\partial y_\alpha } \partial _\gamma {\mathcal {C}}_{\nu {\beta }}\\&+\partial _\gamma g_{\alpha \nu }{{\mathcal {P}}}^\nu _{\beta }+\partial _\gamma g_{\mu \nu }{{\mathcal {P}}}^\mu _\alpha \frac{\partial x_\nu }{\partial y_{\beta }} +\partial _{x_m}[g_{\mu \nu }(x) \frac{\partial x_\mu }{\partial y_\alpha }\frac{\partial x_\nu }{\partial y_{\beta }}]{{\mathcal {P}}}^m_\gamma . \end{aligned}$$

The relation $\tilde{g}^{\alpha {\beta }}\tilde{\Gamma }_{\alpha {\beta },\gamma }=0$ combined with (8.6) and (8.7) implies that

$$\begin{aligned} 0&= g^{mn}(\delta ^\alpha _m+\partial _m \phi _\alpha )(\delta ^{\beta }_n+\partial _n \phi _{\beta })\big [-g_{\mu {\beta }}\partial ^2_{\alpha \gamma }\phi _\mu -g_{\gamma \nu }\partial ^2_{{\beta }\alpha }\phi _\nu +\partial _\alpha g_{\gamma {\beta }}+{\mathcal {K}}_{\gamma {\beta },\alpha }\\&\quad +\frac{1}{2} g_{\mu {\beta }}\partial ^2_{\alpha \gamma }\phi _\mu +\frac{1}{2}g_{\alpha \nu }\partial ^2_{{\beta }\gamma }\phi _\nu -\frac{1}{2}\partial _\gamma g_{\alpha {\beta }}-\frac{1}{2}{\mathcal {K}}_{\alpha {\beta },\gamma }\big ]. \end{aligned}$$

This gives the elliptic equations of $\phi$,

8.8 $$\begin{aligned} \begin{aligned} \Delta \phi _\gamma&= \mathbf {Non}_\gamma (g,\phi ), \end{aligned} \end{aligned}$$

with the boundary condition $\lim _{x\rightarrow \infty }\phi (x)=0$, where the nonlinearities $\mathbf {Non}_\gamma (g,\phi )$ are given by

$$\begin{aligned} \mathbf{Non}_\gamma (g,\phi )&\,{:}{=}\, -h_{\gamma \nu }\Delta \phi _\nu -h^{\alpha {\beta }}g_{\gamma \nu }\partial ^2_{\alpha {\beta }}\phi _\nu + g^{\alpha {\beta }}(\Gamma _{\alpha {\beta },\gamma }+{\mathcal {K}}_{\gamma {\beta },\alpha }-\frac{1}{2}{\mathcal {K}}_{\alpha {\beta },\gamma })\\&\quad +g^{mn}(\delta ^\alpha _m \partial _n \phi _{\beta }+\partial _m \phi _\alpha \delta ^{\beta }_n+\partial _m \phi _\alpha \partial _n \phi _{\beta })\big [-g_{\mu {\beta }}\partial ^2_{\alpha \gamma }\phi _\mu -g_{\gamma \nu }\partial ^2_{{\beta }\alpha }\phi _\nu \\&\quad +\frac{1}{2} g_{\mu {\beta }}\partial ^2_{\alpha \gamma }\phi _\mu +\frac{1}{2}g_{\alpha \nu }\partial ^2_{{\beta }\gamma }\phi _\nu +\Gamma _{\alpha {\beta },\gamma }+\mathcal K_{\gamma {\beta },\alpha }-\frac{1}{2}{\mathcal {K}}_{\alpha {\beta },\gamma }\big ]. \end{aligned}$$

*Step 2: Solve the* $\phi$-*equations* (8.8). By the contraction principle, the existence and uniqueness of solution of (8.8) and the bound (8.1) are obtained by the following Lemma.

#### Lemma 8.3

Let *g* be as in Proposition 8.2. Then the map $\phi \rightarrow \mathbf{Non}_\gamma (g,\phi )$ is Lipschitz from

$$\begin{aligned} H^{s+2} + \dot{H}^2 \rightarrow H^s \end{aligned}$$

with Lipschitz constant $\epsilon$ for $\Vert \nabla ^2 \phi \Vert _{H^s} \lesssim \epsilon$.

#### Proof of Lemma 8.3

In order to prove Lemma 8.3, we consider the following simplified linearization for $\mathbf{Non}_\gamma (g,\phi )$ as a function of $\phi$:

8.9 $$\begin{aligned} \begin{aligned} {\mathcal {T}}(g,\phi ,\Phi )&= h(1+h)\nabla ^2\Phi +g(\nabla h+\delta {\mathcal {K}})\\&\quad +g(\nabla \Phi +\nabla \phi \nabla \Phi )\big [g\nabla ^2\phi +\nabla h+{\mathcal {K}}\big ]\\&\quad +g(\nabla \phi +\nabla \phi \nabla \phi )\big [g\nabla ^2\Phi +\delta {\mathcal {K}}] \end{aligned} \end{aligned}$$

where $\Phi$ is the linearized variable associated to $\phi$, ${\mathcal {K}}$ has the form

$$\begin{aligned} {\mathcal {K}}&\,{:}{=}\, g\nabla ^2\phi {{\mathcal {P}}}+g\nabla {{\mathcal {C}}}(1+{{\mathcal {P}}})+\nabla h {{\mathcal {P}}}(1+{{\mathcal {P}}}) +\nabla [g(1+{{\mathcal {P}}})^2]{{\mathcal {P}}}, \end{aligned}$$

and $$\delta {\mathcal {K}}$$ is

$$\begin{aligned} \delta {\mathcal {K}}&: = g\nabla ^2\Phi {{\mathcal {P}}}+g\nabla ^2\phi \delta {{\mathcal {P}}}+g\nabla \delta {\mathcal {C}}(1+{{\mathcal {P}}})+g\nabla {{\mathcal {C}}}\delta {\mathcal {P}}+\nabla h \delta {\mathcal {P}}(1+{{\mathcal {P}}})\\&\quad +\nabla [g\delta {\mathcal {P}}(1+{{\mathcal {P}}})]{{\mathcal {P}}}+\nabla [g(1+{{\mathcal {P}}})^2]\delta {\mathcal {P}}. \end{aligned}$$

Here ${{\mathcal {C}}}$ and $\delta {{\mathcal {C}}}$ satisfy

$$\begin{aligned} {{\mathcal {C}}}=\nabla \phi \nabla \phi +{{\mathcal {C}}}\nabla \phi ,\quad \delta {{\mathcal {C}}}=\nabla \phi \nabla \Phi +\delta {{\mathcal {C}}}\nabla \phi +{{\mathcal {C}}}\nabla \Phi , \end{aligned}$$

and ${{\mathcal {P}}}$ and $\delta {{\mathcal {P}}}$ are

$$\begin{aligned} {{\mathcal {P}}}=\nabla \phi +{{\mathcal {C}}},\quad \delta {{\mathcal {P}}}=\nabla \Phi +\delta {{\mathcal {C}}}. \end{aligned}$$

Then for the equation (8.9) we have estimates as follows:

#### Lemma 8.4

(Elliptic estimates for (8.9)). Let $d\ge 3$ and $s>d/2$. Assume that $\Vert \nabla h\Vert _{H^s}\lesssim \epsilon$ and $\Vert \nabla ^2\phi \Vert _{H^s}\lesssim \epsilon$, then for the linearized expression (8.9) we have the following estimate

8.10 $$\begin{aligned} \Vert {\mathcal {T}}(g,\phi ,\Phi )\Vert _{H^s}\lesssim \Vert \nabla h\Vert _{H^s}+\epsilon \Vert \nabla ^2 \Phi \Vert _{H^s}. \end{aligned}$$

#### Proof of Lemma 8.4

First, we bound ${{\mathcal {C}}}$, $\delta {\mathcal {C}}$, ${{\mathcal {P}}}$ and $\delta {\mathcal {P}}$. By Sobolev embeddings, using also the smallness condition $\Vert \nabla ^2\phi \Vert _{H^s}\lesssim \epsilon$, we have

$$\begin{aligned} \Vert \nabla {{\mathcal {C}}}\Vert _{H^s}\lesssim \Vert \nabla ^2\phi \Vert _{H^s}^2+\Vert \nabla {{\mathcal {C}}}\Vert _{H^s}\Vert \nabla ^2\phi \Vert _{H^s}\lesssim \epsilon ^2+\epsilon \Vert \nabla {{\mathcal {C}}}\Vert _{H^s}, \end{aligned}$$

and

$$\begin{aligned} \Vert \nabla \delta {\mathcal {C}}\Vert _{H^s}&\lesssim \Vert \nabla ^2\phi \Vert _{H^s}\Vert \nabla ^2\Phi \Vert _{H^s}+\Vert \nabla \delta {\mathcal {C}}\Vert _{H^s}\Vert \nabla ^2\phi \Vert _{H^s}+\Vert \nabla {{\mathcal {C}}}\Vert _{H^s}\Vert \nabla ^2\Phi \Vert _{H^s}\\&\lesssim \epsilon \Vert \nabla ^2\Phi \Vert _{H^s}+\epsilon \Vert \nabla \delta {\mathcal {C}}\Vert _{H^s}+\Vert \nabla {{\mathcal {C}}}\Vert _{H^s}\Vert \nabla ^2\Phi \Vert _{H^s}. \end{aligned}$$

These imply

8.11 $$\begin{aligned} \Vert \nabla {{\mathcal {C}}}\Vert _{H^s}\lesssim \epsilon ^2,\quad \Vert \nabla \delta {\mathcal {C}}\Vert _{H^s}\lesssim \epsilon \Vert \nabla ^2\Phi \Vert _{H^s}. \end{aligned}$$

Similarly we have

8.12 $$\begin{aligned} \Vert \nabla {{\mathcal {P}}}\Vert _{H^s}\lesssim \epsilon ,\quad \Vert \nabla \delta {\mathcal {P}}\Vert _{H^s}\lesssim \Vert \nabla ^2\Phi \Vert _{H^s}. \end{aligned}$$

By Sobolev embedding we bound $\delta {\mathcal {K}}$ by

$$\begin{aligned} \Vert \delta {\mathcal {K}}\Vert _{H^s}&\lesssim (1+\Vert \nabla h\Vert _{H^s})[\Vert \nabla ^2 \Phi \Vert _{H^s}\Vert \nabla {{\mathcal {P}}}\Vert _{H^s}+\Vert \nabla ^2 \phi \Vert _{H^s}\Vert \nabla \delta {\mathcal {P}}\Vert _{H^s}\\&\quad +\Vert \nabla \delta {\mathcal {C}}\Vert _{H^s}(1+\Vert \nabla {{\mathcal {P}}}\Vert _{H^s})+\Vert \nabla {{\mathcal {C}}}\Vert _{H^s}\Vert \nabla \delta {\mathcal {P}}\Vert _{H^s}\\&\quad +\Vert \nabla h\Vert _{H^s}\Vert \nabla \delta {\mathcal {P}}\Vert _{H^s}(1+\Vert \nabla {{\mathcal {P}}}\Vert _{H^s})^2\\&\quad +(1+\Vert \nabla h\Vert _{H^s})\Vert \nabla \delta {\mathcal {P}}\Vert _{H^s}\Vert \nabla {{\mathcal {P}}}\Vert _{H^s}(1+\Vert \nabla {{\mathcal {P}}}\Vert _{H^s})]. \end{aligned}$$

This combined with (8.11) and (8.12) implies

8.13 $$\begin{aligned} \Vert \delta {\mathcal {K}}\Vert _{H^s}\lesssim \epsilon \Vert \nabla ^2\Phi \Vert _{H^s}. \end{aligned}$$

Similarly, we also have

8.14 $$\begin{aligned} \Vert {\mathcal {K}}\Vert _{H^s}\lesssim \epsilon ^2. \end{aligned}$$

Now by Sobolev embedding we bound ${\mathcal {T}}(g,\phi ,\Phi )$ by

$$\begin{aligned} \Vert {\mathcal {T}}\Vert _{H^s}&\lesssim \Vert \nabla h\Vert _{H^s}(1+\Vert \nabla h\Vert _{H^s})(1+\Vert \nabla ^2 \Phi \Vert _{H^s})+(1+\Vert \nabla h\Vert _{H^s})\Vert \delta {\mathcal {K}}\Vert _{H^s}\\&\quad +(1+\Vert \nabla h\Vert _{H^s})\Vert \nabla ^2 \Phi \Vert _{H^s}(1+\Vert \nabla ^2 \phi \Vert _{H^s})\\&\quad \cdot [(1+\Vert \nabla h\Vert _{H^s})\Vert \nabla ^2 \phi \Vert _{H^s}+\Vert \nabla h\Vert _{H^s}+\Vert {\mathcal {K}}\Vert _{H^s}]\\&\quad +(1+\Vert \nabla h\Vert _{H^s})\Vert \nabla ^2 \phi \Vert _{H^s}(1+\Vert \nabla ^2 \phi \Vert _{H^s})[(1+\Vert \nabla h\Vert _{H^s})\Vert \nabla ^2 \Phi \Vert _{H^s}+\Vert \delta {\mathcal {K}}\Vert _{H^s}]. \end{aligned}$$

By the assumptions, (8.14) and (8.13), this gives

$$\begin{aligned} \Vert {\mathcal {T}}(g,\phi ,\Phi )\Vert _{H^s}\lesssim \Vert \nabla h\Vert _{H^s}+\epsilon \Vert \nabla ^2\Phi \Vert _{H^s}. \end{aligned}$$

We conclude the proof of the lemma. $\square$

We continue to prove Lemma 8.3. With small Lipschitz constant $\epsilon$ for $\Vert \nabla ^2\phi \Vert _{H^s}\lesssim \epsilon$, by (8.10) we have

$$\begin{aligned} \Vert \mathbf {Non}_\gamma (g,\phi )\Vert _{H^s}\lesssim \Vert \nabla h\Vert _{H^s}+\epsilon ^2, \end{aligned}$$

and

$$\begin{aligned} \Vert \mathbf {Non}_\gamma (g,\phi )-\mathbf {Non}_\gamma (g,{\tilde{\phi }})\Vert _{H^s}\lesssim \epsilon \Vert \nabla ^2(\phi -{\tilde{\phi }})\Vert _{H^s}. \end{aligned}$$

These give the Lipschitz continuity, completing the proof of Lemma 8.3. $\square$

*Step 3: Prove the bound* (8.2). First we prove the following bound

8.15 $$\begin{aligned} \Vert (\partial _y {{\tilde{h}}})(y(x))\Vert _{H^s(dx)}\lesssim \Vert \partial _x h\Vert _{H^s(dx)}. \end{aligned}$$

By (8.5), it suffices to bound

$$\begin{aligned} \Vert (1+{{\mathcal {P}}})\partial _x[g(1+{{\mathcal {P}}})^2]\Vert _{H^s}&\lesssim \Vert \partial _x[g(1+{{\mathcal {P}}})^2]\Vert _{H^s}(1+\Vert \nabla {{\mathcal {P}}}\Vert _{H^s})\\&\lesssim \Vert \partial _x g\Vert _{H^s}(1+\Vert \nabla {{\mathcal {P}}}\Vert _{H^s})^3\\&\quad +\Vert \partial _x {{\mathcal {P}}}\Vert _{H^s}(1+\Vert \partial _x h\Vert _{H^s})(1+\Vert \nabla {{\mathcal {P}}}\Vert _{H^s})^2\\&\lesssim (\Vert \partial _x g\Vert _{H^s}+\Vert \partial _x {{\mathcal {P}}}\Vert _{H^s})(1+\epsilon )^3\lesssim \Vert \partial _x h\Vert _{H^s}. \end{aligned}$$

This gives the bound (8.15).

In order to complete the proof, we also need the following lemma:

#### Lemma 8.5

Let the change of coordinates $x+\phi (x)=y$ be as in Proposition 8.2. Define the linear operator *T* as $T(f)(y)=f(x(y))$ for any function $f\in L^2(dx)$. Then we have

8.16 $$\begin{aligned} \Vert T(f)(y)\Vert _{H^\sigma (dy)}\lesssim \Vert f(x)\Vert _{H^\sigma (dx)}, \qquad \sigma \in [0,[s]+1]. \end{aligned}$$

Given this lemma, the bound (8.2) is obtained by (8.15) and (8.16) with $\sigma = s$, and the proof of Proposition 8.2 is concluded. It remains to prove the Lemma.

#### Proof of Lemma 8.5

Let *k* be an integer $k\in [0,[s]+1]$, where [*s*] is the integer part of *s*. By the change of coordinates $x+\phi (x)=y$, we have

$$\begin{aligned} \partial _y^k T(f)(y)=[\frac{\partial x}{\partial y}\frac{\partial }{\partial x}]^k f(x)\approx [(1+{{\mathcal {P}}})\partial _x]^k f(x). \end{aligned}$$

It suffices to consider the following forms

$$\begin{aligned} \sum _{\begin{array}{c} 1\le i\le k-1,\ l+l_1+\cdots +l_{i}= k,\\ l\ge 1,\ l_1\ge \cdots \ge l_{i}\ge 1 \end{array}}\partial _x^{l}f\partial _x^{l_1}{{\mathcal {P}}}\cdots \partial _x^{l_{i}}{{\mathcal {P}}}(1+{{\mathcal {P}}})^{k-i}. \end{aligned}$$

By Sobolev embedding, we bound each terms by

$$\begin{aligned} \Vert \partial _x^{l}f\partial _x^{l_1}{{\mathcal {P}}}\cdots \partial _x^{l_{i}}{{\mathcal {P}}}(1+{{\mathcal {P}}})^{k-i}\Vert _{L^2(dy)}&\lesssim \Vert \partial _x^{l}f\partial _x^{l_1}{{\mathcal {P}}}\cdots \partial _x^{l_{i}}{{\mathcal {P}}}(1+{{\mathcal {P}}})^{k-i} \sqrt{\det (I+\partial _x \phi )}\Vert _{L^2(dx)}\\&\lesssim \Vert \partial _x^{l}f\partial _x^{l_1}{{\mathcal {P}}}\cdots \partial _x^{l_{i}}{{\mathcal {P}}}\Vert _{L^2}(1+\Vert \nabla {{\mathcal {P}}}\Vert _{H^s})^{k-i} \Vert 1+\nabla \phi \Vert _{L^\infty }^d\\&\lesssim \Vert f\Vert _{H^k}\Vert \nabla {{\mathcal {P}}}\Vert _{H^s}^i (1+\Vert \nabla h\Vert _{H^s})^{k-i} (1+\Vert \nabla h\Vert _{H^s})^d\\&\lesssim \epsilon ^i\Vert f\Vert _{H^k}. \end{aligned}$$

Then we have

$$\begin{aligned} \Vert \partial _y^k T(f)(y)\Vert _{L^2(dy)}\lesssim \sum _{i=0}^{k-1}\epsilon ^i\Vert f(x)\Vert _{H^k(dx)}\lesssim \Vert f(x)\Vert _{H^k(dx)}. \end{aligned}$$

This implies

$$\begin{aligned} \Vert T(f)(y)\Vert _{H^k(dy)}\lesssim \Vert f(x)\Vert _{H^k(dx)},\quad \text {for any }k\in [0,[s]+1]. \end{aligned}$$

Thus the bound (8.16) is obtained if $\sigma \in [0,[s]+1]$ is an integer. The similar bound for noninteger $\sigma$ follows by interpolation. $\square$

*Step 4: Prove the bound* (8.3). We first prove that the $\partial ^2_{y_\alpha y_{\beta }} {\tilde{F}}\in H^s$ is also small under the above change of coordinates as follows.

#### Proposition 8.6

Let $d\ge 3$, $s>\frac{d}{2}$, and $F:({{\mathbb {R}}}^d_x,g)\rightarrow ({{\mathbb {R}}}^{d+2},g_{{{\mathbb {R}}}^{d+2}})$ be an immersion as in Theorem 8.1. Under the change of coordinates $y=x+\phi (x)$ as in Proposition 8.2, we also have

8.17 $$\begin{aligned} \Vert \partial ^2_{y_\alpha y_{\beta }}{\tilde{F}}\Vert _{H^s(dy)}\lesssim \epsilon _0. \end{aligned}$$

Once the bound (8.17) holds, by (8.2) and Sobolev embedding we obtain the bound (8.3). Here we turn our attention to the proof of Proposition 8.6 and complete the proof of Proposition 8.2.

#### Proof of Proposition 8.6

Here we first prove that $\partial ^2 F$ is also small in $H^s$. Precisely, by the smallness of $\partial _x g$ and Sobolev embedding, we have

$$\begin{aligned} \Vert g^{\alpha {\beta }}\Gamma _{\alpha {\beta }}^\gamma \partial _\gamma F\Vert _{H^s}&\lesssim \Vert g^{\alpha {\beta }}\Gamma _{\alpha {\beta }}^\gamma \Vert _{H^s}+\Vert g^{\alpha {\beta }}\Gamma _{\alpha {\beta }}^\gamma (\partial _\gamma F-\partial _\gamma F(\infty ))\Vert _{H^s}\\&\lesssim \Vert \partial _x h\Vert _{H^s}(1+\Vert \partial _x h\Vert _{H^s})(1+\Vert \partial ^2 F\Vert _{H^s})\lesssim \epsilon _0 (1+\Vert \partial ^2 F\Vert _{H^s}). \end{aligned}$$

Then we can bound $\partial ^2 F$ by

$$\begin{aligned} \Vert \partial ^2 F\Vert _{H^s}&= \Vert {\mathcal {R}} \Delta F\Vert _{H^s}\lesssim \Vert \Delta F\Vert _{H^s}\lesssim \Vert g^{\alpha {\beta }}\partial ^2_{\alpha {\beta }} F\Vert _{H^s}+\epsilon _0 \Vert \partial ^2 F\Vert _{H^s}\\&\lesssim \Vert \Delta _g F\Vert _{H^s}+\Vert g^{\alpha {\beta }} \Gamma _{\alpha {\beta }}^\gamma \partial _\gamma F \Vert _{H^s}+\epsilon _0 \Vert \partial ^2 F\Vert _{H^s}\\&\lesssim \Vert {\mathbf {H}}\Vert _{H^s}+\epsilon _0(1+ \Vert \partial ^2 F\Vert _{H^s})\\&\lesssim \epsilon _0(1+ \Vert \partial ^2 F\Vert _{H^s}), \end{aligned}$$

which implies

8.18 $$\begin{aligned} \Vert \partial ^2 F\Vert _{H^s}\lesssim \epsilon _0. \end{aligned}$$

Next, we turn to prove the bound (8.17). By the change of coordinates, we have the representation $\partial ^2_{y_\alpha y_{\beta }}{\tilde{F}}$ as

$$\begin{aligned} \partial ^2_{y_\alpha y_{\beta }}{\tilde{F}} =\partial _{y_\alpha } (\partial _\gamma F \frac{\partial x_\gamma }{\partial y_{\beta }})=\partial ^2_{\sigma \gamma }F \frac{\partial x_\sigma }{\partial y_\alpha }\frac{\partial x_\gamma }{\partial y_{\beta }}+\partial _\gamma F \frac{\partial }{\partial y_\alpha }\frac{\partial x_\gamma }{\partial y_{\beta }}. \end{aligned}$$

Since $\frac{\partial x_\gamma }{\partial y_{\beta }}$ is a function depending on *x* and has the form $\frac{\partial x}{\partial y}=I_d+{\mathcal {P}}(x)$, we write this as

$$\begin{aligned} \partial ^2_{y_\alpha y_{\beta }}{\tilde{F}}&= \partial ^2_{\sigma \gamma }F (I_d+{\mathcal {P}})^2+\partial _\gamma F \partial _x (I_d+{{\mathcal {P}}}) \cdot (I_d+{{\mathcal {P}}})\\&= \partial ^2_{\sigma \gamma }F (I_d+{\mathcal {P}})^2+\partial _\gamma F \partial _x {{\mathcal {P}}}\cdot (I_d+{{\mathcal {P}}}). \end{aligned}$$

As a vector depends on *x*, by Sobolev embedding, (8.18) and (8.12) we have

$$\begin{aligned} \Vert (\partial ^2_{y_\alpha y_{\beta }}{\tilde{F}})(x)\Vert _{H^s(dx)}&\lesssim \Vert \partial ^2_{\sigma \gamma }F\Vert _{H^s} (1+\Vert \partial _x{\mathcal {P}}\Vert _{H^s}^2)+(1+\Vert \partial ^2 F\Vert _{H^s})\Vert \partial _x {{\mathcal {P}}}\Vert _{H^s}(1+\Vert \partial _x {{\mathcal {P}}}\Vert _{H^s})\\&\lesssim \epsilon _0. \end{aligned}$$

Then by Lemma 8.5, the bound (8.17) follows. $\square$

*Step 5: Prove the bound* (8.4). Finally, we construct the initial data $\psi _0$ in the harmonic coordinates and Coulomb gauge. To obtain the Coulomb gauge, we choose $\tilde{\nu }$ constant uniformly transversal to $T \Sigma _0$; such a $\nu$ exists because, by Sobolev embeddings, $\partial _x F$ has a small variation in $L^\infty$. Projecting $\tilde{\nu }$ on the normal bundle $N \Sigma _0$ and normalizing we obtain some $\tilde{\nu }_1$ with the same regularity as $\partial F$. Then we choose $\tilde{\nu }_2$ in $N \Sigma _0$ perpendicular to $\tilde{\nu }_1$. We obtain the orthonormal frame $(\tilde{\nu }_1,\tilde{\nu }_2)$ in $N \Sigma _0$, which again has the same regularity and bounds as $\partial _x F$. Then we rotate the frame to get a Coulomb frame $(\nu _1,\nu _2)$, i.e. where the Coulomb gauge condition (2.16) is satisfied. Projecting the mean curvature ${\mathbf {H}}$ on the Coulomb frame as in Sect. 2.3 we obtain the complex mean curvature $\psi \in H^s$.

In order to get the bound for $\psi$, we recall that the second fundamental form $\lambda$ satisfies

$$\begin{aligned} \lambda _{\alpha {\beta }}=(\partial ^2_{\alpha {\beta }}F)^{\perp } \cdot \nu _1+i(\partial ^2_{\alpha {\beta }}F)^{\perp } \cdot \nu _2. \end{aligned}$$

We easily have

$$\begin{aligned} \Vert \lambda \Vert _{L^2}\lesssim \Vert \partial ^2 F\Vert _{L^2}\lesssim \epsilon _0. \end{aligned}$$

Then it suffices to bound the ${\dot{H}}^s$ norm of $\lambda$. If $s\in {\mathbb {N}}$, we have

$$\begin{aligned} \Vert \lambda \Vert _{{\dot{H}}^s}&\lesssim \sum _{\nu \in \{\nu _1,\nu _2\};n_1+n_2=s}\Vert \partial ^{n_1}(\partial ^2_{\alpha {\beta }}F-\Gamma _{\alpha {\beta }}^\gamma F_\gamma )\cdot \partial ^{n_2}\nu \Vert _{L^2}\\&\lesssim \sum _{\nu \in \{\nu _1,\nu _2\}}\Vert (\nabla _x+A)^{s}\nabla _\alpha \partial _{\beta }F\cdot \nu \Vert _{L^2}\\&\lesssim \Vert (\nabla _x+A)^{s}\nabla _\alpha \partial _{\beta }F\Vert _{L^2}\\&\lesssim \Vert \partial ^2 F\Vert _{H^s}(1+\Vert \nabla A\Vert _{H^s}^{s}). \end{aligned}$$

If $s \notin {\mathbb {N}}$, let $\frac{1}{p}+\frac{1}{q}=\frac{1}{2}$ we also have

$$\begin{aligned} \Vert \lambda \Vert _{{\dot{H}}^s}&\lesssim \sum _{\nu \in \{\nu _1,\nu _2\};n_1+n_2=[s]-1}\Vert |D|^{1+s-[s]} \big (\partial ^{n_1}(\partial ^2_{\alpha {\beta }}F-\Gamma _{\alpha {\beta }}^\gamma F_\gamma )\cdot \partial ^{n_2}\nu \big )\Vert _{L^2}\\&\lesssim \sum _{\nu \in \{\nu _1,\nu _2\}}\Vert |D|^{1+s-[s]} \big ((\nabla _x+A)^{[s]-1}\nabla _\alpha \partial _{\beta }F\cdot \nu \big )\Vert _{L^2}\\&\lesssim \sum _{\nu \in \{\nu _1,\nu _2\}}\big (\Vert |D|^{1+s-[s]} (\nabla _x+A)^{[s]-1}\nabla _\alpha \partial _{\beta }F\cdot \nu \Vert _{L^2}\\&\quad +\Vert (\nabla _x+A)^{[s]-1}\nabla _\alpha \partial _{\beta }F\cdot |D|^{1+s-[s]}\nu \Vert _{L^2}\big )\\&\lesssim \Vert |D|^{1+s-[s]} (\nabla _x+A)^{[s]-1}\nabla _\alpha \partial _{\beta }F\Vert _{L^2}\\&\quad +\Vert (\nabla _x+A)^{[s]-1}\nabla _\alpha \partial _{\beta }F\Vert _{L^p}\sum _{\nu \in \{\nu _1,\nu _2\}}\Vert |D|^{1+s-[s]}\nu \Vert _{L^q}\\&= I_1+I_2. \end{aligned}$$

We bound the first term by

$$\begin{aligned} I_1\lesssim \Vert \partial ^2 F\Vert _{H^s}(1+\Vert \nabla A\Vert _{H^s}^{[s]})&\lesssim \epsilon _0 (1+\Vert \nabla A\Vert _{H^s}^{[s]}). \end{aligned}$$

For the second term, we choose integer $k=[\frac{d+1}{2}]$ and $\frac{1}{p}=\frac{k-1-(s-[s])}{d}$, then we have

$$\begin{aligned} I_2&\lesssim \Vert (\nabla _x+A)^{[s]-1}\nabla _\alpha \partial _{\beta }F\Vert _{H^{1+s-[s]}}\sum _{\nu \in \{\nu _1,\nu _2\}}\Vert \partial ^k\nu \Vert _{L^2}\\&\lesssim \Vert \partial ^2 F\Vert _{H^s}(1+\Vert \nabla A\Vert _{H^s}^{[s]})(1+\Vert A\Vert _{H^{s}}+\Vert \lambda \Vert _{H^s})^{k}. \end{aligned}$$

Therefore, by the elliptic estimates of the div-curl system (2.13)–(2.16) for *A* we obtain

$$\begin{aligned} \Vert \lambda \Vert _{H^s}&\lesssim \epsilon _0 (1+\Vert \nabla A\Vert _{H^s}^{[s]})(1+\Vert A\Vert _{H^{s}}+\Vert \lambda \Vert _{H^s})^{k}\lesssim \epsilon _0 (1+\Vert \lambda \Vert _{H^s})^{4[s]+1}\\&\lesssim \epsilon _0 +\epsilon _0\Vert \lambda \Vert _{H^s}^{4[s]+1}. \end{aligned}$$

By continuity method, this implies the bound

$$\begin{aligned} \Vert \lambda \Vert _{H^s}\lesssim \epsilon _0. \end{aligned}$$

which combined with the smallness of $\partial _x g\in H^s$ also gives the bound (8.4) for $\psi$. $\square$

###  The moving frame

Once we have the initial data $\psi _0$ which is small in $H^s$, Theorem 2.7 yields the good gauge local solution $\psi$, along with the associated derived variables $(\lambda ,h,V,A,B)$. But this does not yet give us the actual maps *F*.

Here we undertake the task of reconstructing the frame $(F_\alpha , m)$. For this we use the system consisting of (2.6) and (2.25), viewed as a linear ode. We recall these equations here:

8.19 $$\begin{aligned} \left\{ \begin{aligned}&\partial _{\alpha }F_{{\beta }}=\Gamma ^{\gamma }_{\alpha {\beta }}F_{\gamma }+\mathop {\mathrm{Re}}\nolimits (\lambda _{\alpha {\beta }}{\bar{m}}),\\&\partial _{\alpha }^A m=-\lambda ^{\gamma }_{\alpha } F_{\gamma }, \end{aligned}\right. \end{aligned}$$

respectively

8.20 $$\begin{aligned} \left\{ \begin{aligned}&\partial _t F_{\alpha }=-\mathop {\mathrm{Im}}\nolimits (\partial ^A_{\alpha } \psi {\bar{m}}-i\lambda _{\alpha \gamma }V^{\gamma } {\bar{m}})+[\mathop {\mathrm{Im}}\nolimits (\psi {\bar{\lambda }}^{\gamma }_{\alpha })+\nabla _{\alpha } V^{\gamma }]F_{\gamma },\\&\partial ^{B}_t m=-i(\partial ^{A,\alpha } \psi -i\lambda ^{\alpha }_{\gamma }V^{\gamma } )F_{\alpha }, \end{aligned}\right. \end{aligned}$$

where $(\psi ,\lambda ,g,V,A,B)$ is the unique solution of (2.35)–(2.36) with initial data $\psi _0$ small.

We start with the frame at time $t=0$, which already is known to solve (8.19), and has the following properties:

(i) *Orthogonality*, $F_\alpha \perp m$, $\langle m,m\rangle =2$, $\langle m,{\bar{m}}\rangle =0$ and consistency with the metric $g_{\alpha {\beta }} = \langle F_\alpha ,F_{\beta }\rangle$.
(ii) *Integrability*, $$\partial _\beta F_\alpha = \partial _\alpha F_\beta $$.
(iii) *Consistency* with the second fundamental form and the connection *A*:
  $$\begin{aligned} \partial _\alpha F_{\beta }\cdot m=\lambda _{\alpha {\beta }}, \qquad \langle \partial _\alpha m,m\rangle = -2 i A_\alpha . \end{aligned}$$

Next we extend this frame to times $t > 0$ by simultaneously solving the pair of equations (8.19) and (8.20). To avoid some technical difficulties, we first do this for regular solutions, i.e. $s > d/2 +2$, and then pass to the limit to obtain the frame for rough solutions.

#### The frame associated to smooth solutions

The system consisting of (8.19) and (8.20) is overdetermined, and the necessary and sufficient condition for existence of solutions is provided by Frobenius’ theorem. We now verify these compatibility conditions in two steps:

a) Compatibility conditions for the system (8.19) at fixed time. Here, by $C^2_{\alpha {\beta }}=0$, $C^3_{\alpha {\beta }}=0$ and $C^7_{\alpha {\beta }\mu \nu }=0$ we have

$$\begin{aligned}&\partial _{\alpha }(\Gamma ^\sigma _{{\beta }\gamma }F_\sigma +\mathop {\mathrm{Re}}\nolimits (\lambda _{{\beta }\gamma }{\bar{m}}))-\partial _{{\beta }}(\Gamma ^\sigma _{\alpha \gamma }F_\sigma +\mathop {\mathrm{Re}}\nolimits (\lambda _{\alpha \gamma }{\bar{m}})) = C^7_{\sigma \gamma \alpha {\beta }}F^\sigma =0, \end{aligned}$$

and

$$\begin{aligned} \partial _\alpha (iA_{\beta }m+\lambda ^\sigma _{\beta }F_\sigma )-\partial _{\beta }(iA_\alpha m+\lambda ^\sigma _\alpha F_\sigma )=iC^3_{\alpha {\beta }}m=0, \end{aligned}$$

as needed.

b) Between the system (8.19) and (8.20). By (8.19) and (8.20) we have

$$\begin{aligned} \partial _t(iA_\alpha m+\lambda ^\sigma _\alpha F_\sigma )-\partial _\alpha (iB m+i(\partial ^{A,\sigma }\psi -i\lambda ^\sigma _\gamma V^\gamma )F_\sigma ) = iT^3_{\alpha }m +T^{2\sigma }_\alpha F_\sigma \end{aligned}$$

and

8.21 $$\begin{aligned} \begin{aligned}&\partial _{\beta }[-\mathop {\mathrm{Im}}\nolimits (\partial ^A_{\alpha } \psi {\bar{m}}-i\lambda _{\alpha \gamma }V^{\gamma } {\bar{m}})+[\mathop {\mathrm{Im}}\nolimits (\psi {\bar{\lambda }}^{\gamma }_{\alpha })+\nabla _{\alpha } V^{\gamma }]F_{\gamma }]-\partial _t[\Gamma ^\gamma _{{\beta }\alpha }F_\gamma +\mathop {\mathrm{Re}}\nolimits (\lambda _{{\beta }\alpha }{\bar{m}})]\\&= -\mathop {\mathrm{Re}}\nolimits [(g_{\sigma \alpha }T^{2\sigma }_{\beta }+\lambda ^\sigma _{\beta }T^1_{\sigma \alpha }){\bar{m}}] -T^{1\gamma \sigma }\Gamma _{{\beta }\alpha ,\sigma }F_\gamma -\frac{1}{2}(\partial _{\beta }T^1_{\alpha \sigma }+\partial _\alpha T^1_{{\beta }\sigma }-\partial _\sigma T^1_{{\beta }\alpha })F^\sigma . \end{aligned} \end{aligned}$$

The first equality is obtained directly. For the second equality (8.21), by (8.19) and (8.20) we compute this by

$$\begin{aligned} \mathrm{LHS}(8.21) =&-\mathop {\mathrm{Re}}\nolimits [(g_{\sigma \alpha }T^{2\sigma }_{\beta }+\lambda ^\sigma _{\beta }T^1_{\sigma \alpha }){\bar{m}}] +\nabla _{\beta }(\mathop {\mathrm{Im}}\nolimits (\psi {\bar{\lambda }}_{\sigma \alpha })+\nabla _\alpha V_\sigma ) F^\sigma \\&+\mathop {\mathrm{Im}}\nolimits (\nabla ^A_\alpha \psi {\bar{\lambda }}_{\sigma {\beta }}-\nabla ^A_\sigma \psi {\bar{\lambda }}_{\alpha {\beta }})F^\sigma -{\tilde{R}}_{\sigma \alpha {\beta }\gamma }V^\gamma F^\sigma -\partial _t \Gamma ^\gamma _{{\beta }\alpha } F_\gamma . \end{aligned}$$

By $T^1$ and the notation $G_{\alpha {\beta }}$ (2.29) we compute the last term by

$$\begin{aligned} -\partial _t \Gamma ^\gamma _{{\beta }\alpha } F_\gamma =&-(T^{1\gamma \sigma }-2G^{\gamma \sigma })\Gamma _{{\beta }\alpha ,\sigma }F_\gamma -\frac{1}{2}[\partial _{\beta }(T^1_{\alpha \sigma }+2G_{\alpha \sigma })]F^\sigma \\&-\frac{1}{2}[\partial _\alpha (T^1_{{\beta }\sigma }+2G_{{\beta }\sigma })]F^\sigma +\frac{1}{2}[\partial _\sigma (T^1_{{\beta }\alpha }+2G_{{\beta }\alpha })]F^\sigma \\ =&-T^{1\gamma \sigma }\Gamma _{{\beta }\alpha ,\sigma }F_\gamma -\frac{1}{2}(\partial _{\beta }T^1_{\alpha \sigma }+\partial _\alpha T^1_{{\beta }\sigma }-\partial _\sigma T^1_{{\beta }\alpha })F^\sigma \\&+[-\nabla _{\beta }\mathop {\mathrm{Im}}\nolimits (\psi {\bar{\lambda }}_{\alpha \sigma })-\mathop {\mathrm{Im}}\nolimits (\nabla ^A_\alpha \psi {\bar{\lambda }}_{{\beta }\sigma })+\mathop {\mathrm{Im}}\nolimits (\nabla ^A_\sigma \psi {\bar{\lambda }}_{{\beta }\alpha })\\&-\frac{1}{2}(\nabla _\alpha \nabla _{\beta }+\nabla _{\beta }\nabla _\alpha )V_\sigma -\frac{1}{2}[\nabla _{\beta },\nabla _\sigma ]V_\alpha -\frac{1}{2}[\nabla _\alpha ,\nabla _\sigma ]V_{\beta }]F^\sigma . \end{aligned}$$

Then by Bianchi identities and (2.8), we collect the terms above containing *V* and have

$$\begin{aligned}&\frac{1}{2}([\nabla _{\beta },\nabla _\alpha ]V_\sigma -[\nabla _{\beta },\nabla _\sigma ]V_\alpha -[\nabla _\alpha ,\nabla _\sigma ]V_{\beta })-{\tilde{R}}_{\sigma \alpha {\beta }\gamma }V^\gamma \\&= \frac{1}{2}( R_{{\beta }\alpha \sigma \gamma }-R_{{\beta }\sigma \alpha \gamma }-R_{\alpha \sigma {\beta }\gamma }-2R_{\sigma \alpha {\beta }\gamma })V^\gamma =0. \end{aligned}$$

From the above expressions the equality (8.21) follows.

Once the compatibility conditions in Frobenius’ theorem are verified, we obtain the frame $(F_\alpha ,m)$ for $t \in [0,1]$. For this we can easily obtain the regularity

$$\begin{aligned} \partial _x(F_\alpha , m) \in L^\infty H^{s+2}, \qquad \partial _t (F_\alpha , m) \in L^\infty H^{s+1}. \end{aligned}$$

Finally, we show that the properties (i)–(iii) above also extend to all $t \in [0,1]$. The properties (ii) and (iii) follow directly from the equations (8.19) and (8.20) once the orthogonality conditions in (i) are verified. For (i) we denote

$$\begin{aligned} {{\tilde{g}}}_{00}=\langle m,m\rangle ,\quad {{\tilde{g}}}_{\alpha 0}=\langle F_\alpha , m\rangle ,\quad {{\tilde{g}}}_{\alpha {\beta }}=\langle F_\alpha ,F_{\beta }\rangle . \end{aligned}$$

Then by (8.20) and $T^1_{\alpha {\beta }}=0$, we have

$$\begin{aligned}&\begin{aligned} \partial _t {{\tilde{g}}}_{\alpha 0}&= -\frac{i}{2}(\overline{\partial ^A_\alpha \psi }+i {\bar{\lambda }}_{\alpha \gamma }V^\gamma )({{\tilde{g}}}_{00}-2)-i(\overline{\partial ^{A,\sigma } \psi }+i {\bar{\lambda }}^\sigma _{\gamma }V^\gamma )(g_{\alpha \sigma }-{{\tilde{g}}}_{\alpha \sigma })\\&\quad +\frac{i}{2}(\partial ^A_\alpha \psi +i \lambda _{\alpha \gamma }V^\gamma )\langle {\bar{m}},m\rangle +(\mathop {\mathrm{Im}}\nolimits (\psi {\bar{\lambda }}_\alpha ^\gamma )+\nabla _\alpha V^\gamma ){{\tilde{g}}}_{\gamma 0}+iB {{\tilde{g}}}_{\alpha 0}, \end{aligned} \\&\partial _t ({{\tilde{g}}}_{00}-2)=2\mathop {\mathrm{Im}}\nolimits (\partial ^{A,\alpha }\psi -i \lambda ^\alpha _\gamma V^\gamma ) {{\tilde{g}}}_{\alpha 0},\\&\partial _t \langle m,{\bar{m}}\rangle = -iB\langle m,{\bar{m}}\rangle -i (\partial ^{A,\alpha }\psi -i \lambda ^\alpha _\gamma V^\gamma ) {{\tilde{g}}}_{\alpha 0},\\&\begin{aligned} \partial _t(g_{\alpha {\beta }}-{{\tilde{g}}}_{\alpha {\beta }})&= (\mathop {\mathrm{Im}}\nolimits (\psi {\bar{\lambda }}_\alpha ^\gamma )+\nabla _\alpha V^\gamma )(g_{{\beta }\gamma }-{{\tilde{g}}}_{{\beta }\gamma })+(\mathop {\mathrm{Im}}\nolimits (\psi {\bar{\lambda }}_{\beta }^\gamma )+\nabla _{\beta }V^\gamma )(g_{\alpha \gamma }-{{\tilde{g}}}_{\alpha \gamma })\\&\quad +\mathop {\mathrm{Im}}\nolimits (\partial ^A_\alpha \psi {{\tilde{g}}}_{{\beta }0}-i\lambda _{\alpha \gamma }V^\gamma {\bar{{{\tilde{g}}}}}_{{\beta }0})+\mathop {\mathrm{Im}}\nolimits (\partial ^A_{\beta }\psi {{\tilde{g}}}_{\alpha 0}-i\lambda _{{\beta }\gamma }V^\gamma {\bar{{{\tilde{g}}}}}_{\alpha 0}). \end{aligned} \end{aligned}$$

Viewed as a linear system of ode’s in time, these equations allow us to propagate (i) in time.

#### The frame associated to rough solutions

Here we use our approximation of rough solutions with smooth solutions for the $\psi$ equation in order to construct the frame in the rough case. Precisely, given a small initial data $\psi _0 \in H^s$, there exists a sequence $\{\psi _{0n}\}\in H^{s+2}$ such that $\Vert \psi _{0n}-\psi _0\Vert _{H^s}\rightarrow 0$. By Theorem 2.7, the Schrödinger system (2.35) coupled with (2.36) admits solutions $\psi _n$ with $\psi _n(0)=\psi _{0n}$ and

$$\begin{aligned} \Vert \psi _n\Vert _{H^{s+2}}\lesssim \Vert \psi _{0n}\Vert _{H^{s+2}},\quad \Vert \psi _n-\psi \Vert _{H^{s}}\lesssim \Vert \psi _{0n}-\psi _0\Vert _{H^{s}}\rightarrow 0. \end{aligned}$$

A-priori, we do not know whether the initial data $\psi _{0n}$ is associated to a frame at the initial time. Hence we first use (8.19) to construct the frame $(F_\alpha ^{(n)},m^{(n)})$ associated with $\psi _{0n}$ at $t=0$. At some point $x_0$, we choose $F^{(n)}_\alpha (x_0)$ and $m^{(n)}(x_0)$ so that they are orthogonal, and $\langle m^{(n)},m^{(n)}\rangle =2$, $\langle m^{(n)},{\bar{m}}^{(n)}\rangle =0$ and $\langle F^{(n)}_\alpha ,F^{(n)}_{\beta }\rangle =g^{(n)}_{\alpha {\beta }}$ hold. With this initial data, we view (8.19) as a linear ode with continuous coefficients. As above, the necessary and sufficient condition for solvability, as provided by Frobenius’ theorem, is a consequence of the relations $C^2=0$, $C^3 = 0$ and $C^7 = 0$, which are in turn a consequence of Theorem 4.1.

The above construction determines the frame $(F^{(n)}_\alpha ,m^{(n)})$ up to symmetries (rigid rotations and translations). Hence, the frame $(F_\alpha ^{(n)},m^{(n)})$ at $t=0$ is uniquely determined by the condition

$$\begin{aligned} \lim _{x\rightarrow \infty }(F_{\alpha }^{(n)},m^{(n)})(x,0)=\lim _{x\rightarrow \infty }(F_\alpha ,m)(x,0). \end{aligned}$$

In this construction, the properties (i)–(iii) above also extend to all *x*. The properties (ii) and (iii) follow directly from equation (8.19) once the orthogonality conditions in (i) are verified. For (i) we use (8.19) to compute

$$\begin{aligned}&\partial _\alpha {{\tilde{g}}}_{{\beta }0}=\Gamma ^\gamma _{\alpha {\beta }} {{\tilde{g}}}_{\gamma 0}+\frac{1}{2}\lambda _{\alpha {\beta }}\langle {\bar{m}},m\rangle +\frac{1}{2}{\bar{\lambda }}_{\alpha {\beta }}({{\tilde{g}}}_{00}-2)+{\bar{\lambda }}^\gamma _\alpha (g_{{\beta }\gamma }-{{\tilde{g}}}_{{\beta }\gamma })+iA_\alpha {{\tilde{g}}}_{{\beta }0},\\&\partial _{\alpha }({{\tilde{g}}}_{00}-2)=-2\mathop {\mathrm{Re}}\nolimits (\lambda ^\gamma _\alpha {{\tilde{g}}}_{\gamma 0}),\\&\partial _\alpha \langle m,{\bar{m}}\rangle =-2i A_\alpha \langle m,{\bar{m}}\rangle -2\mathop {\mathrm{Re}}\nolimits \lambda ^\gamma _\alpha {\bar{{{\tilde{g}}}}}_{\gamma 0},\\&\partial _\alpha (g_{{\beta }\gamma }- {{\tilde{g}}}_{{\beta }\gamma })=\Gamma ^\sigma _{\alpha {\beta }}(g_{\sigma \gamma }-{{\tilde{g}}}_{\sigma \gamma })+\Gamma ^\sigma _{\alpha \gamma }(g_{\sigma {\beta }}-{{\tilde{g}}}_{\sigma {\beta }})+\mathop {\mathrm{Re}}\nolimits ({\bar{\lambda }}_{{\beta }\alpha }{{\tilde{g}}}_{\gamma 0}+{\bar{\lambda }}_{\gamma \alpha }{{\tilde{g}}}_{{\beta }0}). \end{aligned}$$

By ode uniqueness and the choice of the initial data, the desired properties for the frame are propagated spatially.

Once we have the frames $(F_\alpha ^{(n)},m^{(n)})$ at $t=0$, we can invoke the smooth case analysis above, using (8.20) and $\psi _n\in H^{s+2}$ to extend the frame $(F_\alpha ^{(n)},m^{(n)})$ to $t>0$ with initial data $(F_\alpha ^{(n)},m^{(n)})(x,0)$.

In order to obtain a limiting frame $(F_\alpha ,m)$ we study the properties of the regular frames $(F_\alpha ^{(n)}, m^{(n)})$ in three steps:

*a) Uniform bounds.* By (8.19), (2.37) and Sobolev embeddings we have

$$\begin{aligned} \Vert \partial _x F^{(n)}_\alpha \Vert _{H^s}&\lesssim \Vert \Gamma ^{(n)} F^{(n)}_\gamma +\lambda ^{(n)} m^{(n)}\Vert _{H^s}\\&\lesssim \Vert \psi _n\Vert _{H^s}(|F_\alpha (\infty )|+| m(\infty )|+\Vert \partial _x( F^{(n)}_\alpha , m^{(n)})\Vert _{H^s}) \end{aligned}$$

and

$$\begin{aligned} \Vert \partial _x m^{(n)}\Vert _{H^s}&\lesssim \Vert A^{(n)} m^{(n)}+\lambda ^{(n)} F_\alpha ^{(n)}\Vert _{H^s}\\&\lesssim \Vert \psi _n\Vert _{H^s}(|F_\alpha (\infty )|+| m(\infty )|+\Vert \partial _x ( F^{(n)}_\alpha , m^{(n)})\Vert _{H^s}) \end{aligned}$$

Then, by the smallness of $\psi _n\in H^s$, we obtain

$$\begin{aligned} \Vert \partial _x( F^{(n)}_\alpha , m^{(n)})\Vert _{H^s}\lesssim \Vert \psi _n\Vert _{H^s}. \end{aligned}$$

*b) Sobolev and uniform convergence at* $t=0$. Using an argument similar to that in *a)*, by (8.19) and Theorem 4.1*b)* we have

$$\begin{aligned} \Vert \partial _x (F^{(n)}_\alpha -F_\alpha , m^{(n)}-m)\Vert _{H^s}&\lesssim \Vert \psi _{0n}-\psi _0\Vert _{H^s}+\Vert \psi _0\Vert _{H^s}\Vert \partial _x (F^{(n)}_\alpha -F_\alpha , m^{(n)}-m)\Vert _{H^s}. \end{aligned}$$

By the smallness of $\psi _0$, this implies the $H^s$ convergence. The uniform convergence at $t=0$ also follows by Sobolev embeddings.

*c) a.e. convergence for* $t > 0$. Here we use (8.20) as an ode in time. The coefficients converge in $L^2_t$ for a.e. *x*, so the frames $(F^{(n)}_\alpha ,m^{(n)})$ will also converge uniformly in time for a.e. *x*. This can be rectified to uniform convergence in view of the uniform Sobolev bounds in (i). This yields the desired limiting frames $(F_\alpha ,m)$.

By (8.19) we also have

$$\begin{aligned}&\Vert \partial _x (F^{(k)}_\alpha -F^{(l)}_\alpha , m^{(k)}-m^{(l)})\Vert _{L_t^\infty H^s} \lesssim \Vert \psi _{k}-\psi _l\Vert _{L_t^\infty H^s}\lesssim \Vert \psi _{0k}-\psi _{0l}\Vert _{H^s}. \end{aligned}$$

This shows that the limiting frame satisfies both equations (8.20) and (8.19), as well the as the uniform bounds in (a).

### The moving manifold $\Sigma _t$

Here we propagate the full map *F* by simply integrating (2.24), i.e.

$$\begin{aligned} F(t)=F(0)+\int _0^t -\mathop {\mathrm{Im}}\nolimits (\psi {\bar{m}})+V^\gamma F_\gamma ds. \end{aligned}$$

Then by (8.19), we have

$$\begin{aligned} \partial _\alpha F(t)=\partial _\alpha F(0)+\int _0^t -\mathop {\mathrm{Im}}\nolimits (\partial ^A_{\alpha } \psi {\bar{m}}-i\lambda _{\alpha \gamma }V^{\gamma } {\bar{m}})+[\mathop {\mathrm{Im}}\nolimits (\psi {\bar{\lambda }}^{\gamma }_{\alpha })+\nabla _{\alpha } V^{\gamma }]F_{\gamma } ds, \end{aligned}$$

which is consistent with above definition of $F_\alpha$.

### The (SMCF) equation for *F*

Here we establish that *F* solves (1.1). Using the relation $\lambda _{\alpha {\beta }}=\partial ^2_{\alpha {\beta }} F \cdot m$ we have

$$\begin{aligned} -\mathop {\mathrm{Im}}\nolimits (\psi {\bar{m}})&= -\mathop {\mathrm{Im}}\nolimits (g^{\alpha {\beta }}\partial ^2_{\alpha {\beta }} F\cdot (\nu _1+i\nu _2)\ (\nu _1-i\nu _2))\\&= (\Delta _g F\cdot \nu _1) \nu _2-(\Delta _g F\cdot \nu _2) \nu _1\\&= J (\Delta _g F)^{\perp }=J{\mathbf {H}}(F). \end{aligned}$$

This implies that the *F* solves (1.1).
